# Pandanus nutshell generates a palaeoprecipitation record for human occupation at Madjedbebe, northern Australia

**DOI:** 10.1038/s41559-020-01379-8

**Published:** 2021-01-25

**Authors:** S. Anna Florin, Patrick Roberts, Ben Marwick, Nicholas R. Patton, James Shulmeister, Catherine E. Lovelock, Linda A. Barry, Quan Hua, May Nango, Djaykuk Djandjomerr, Richard Fullagar, Lynley A. Wallis, Andrew S. Fairbairn, Chris Clarkson

**Affiliations:** 1 School of Social Science, The University of Queensland, Brisbane, Queensland Australia; 2 Australian Research Council Centre of Excellence for Australian Biodiversity and Heritage, University of Wollongong, Wollongong, New South Wales Australia; 3 Department of Archaeology, Max Planck Institute for the Science of Human History, Jena, Germany; 4 Department of Anthropology, University of Washington, Seattle, WA USA; 5 School of Earth and Environment, University of Canterbury, Christchurch, New Zealand; 6 School of Earth and Environmental Sciences, The University of Queensland, Brisbane, Queensland Australia; 7 School of Biological Sciences, The University of Queensland, Brisbane, Queensland Australia; 8 Australian Nuclear Science and Technology Organisation, Lucas Heights, New South Wales Australia; 9 Gundjeihmi Aboriginal Corporation, Jabiru, Northern Territory Australia; 10 Centre for Archaeological Science, School of Earth, Atmospheric and Life Sciences, University of Wollongong, Wollongong, New South Wales Australia; 11 Griffith Centre for Social and Cultural Research, Griffith University, Nathan, Queensland Australia

**Keywords:** Archaeology, Palaeoecology, Archaeology, Plant ecology

## Abstract

Little is known about the Pleistocene climatic context of northern Australia at the time of early human settlement. Here we generate a palaeoprecipitation proxy using stable carbon isotope analysis of modern and archaeological pandanus nutshell from Madjedbebe, Australia’s oldest known archaeological site. We document fluctuations in precipitation over the last 65,000 years and identify periods of lower precipitation during the penultimate and last glacial stages, Marine Isotope Stages 4 and 2. However, the lowest effective annual precipitation is recorded at the present time. Periods of lower precipitation, including the earliest phase of occupation, correspond with peaks in exotic stone raw materials and artefact discard at the site. This pattern is interpreted as suggesting increased group mobility and intensified use of the region during drier periods.

## Abstract (web summary)

Stable carbon isotope analysis of modern and archaeological pandanus nutshell from Australia’s oldest known archaeological site of Madjedbebe reveals precipitation fluctuations over the last 65,000 years.

## Main

**Fig. 1:**
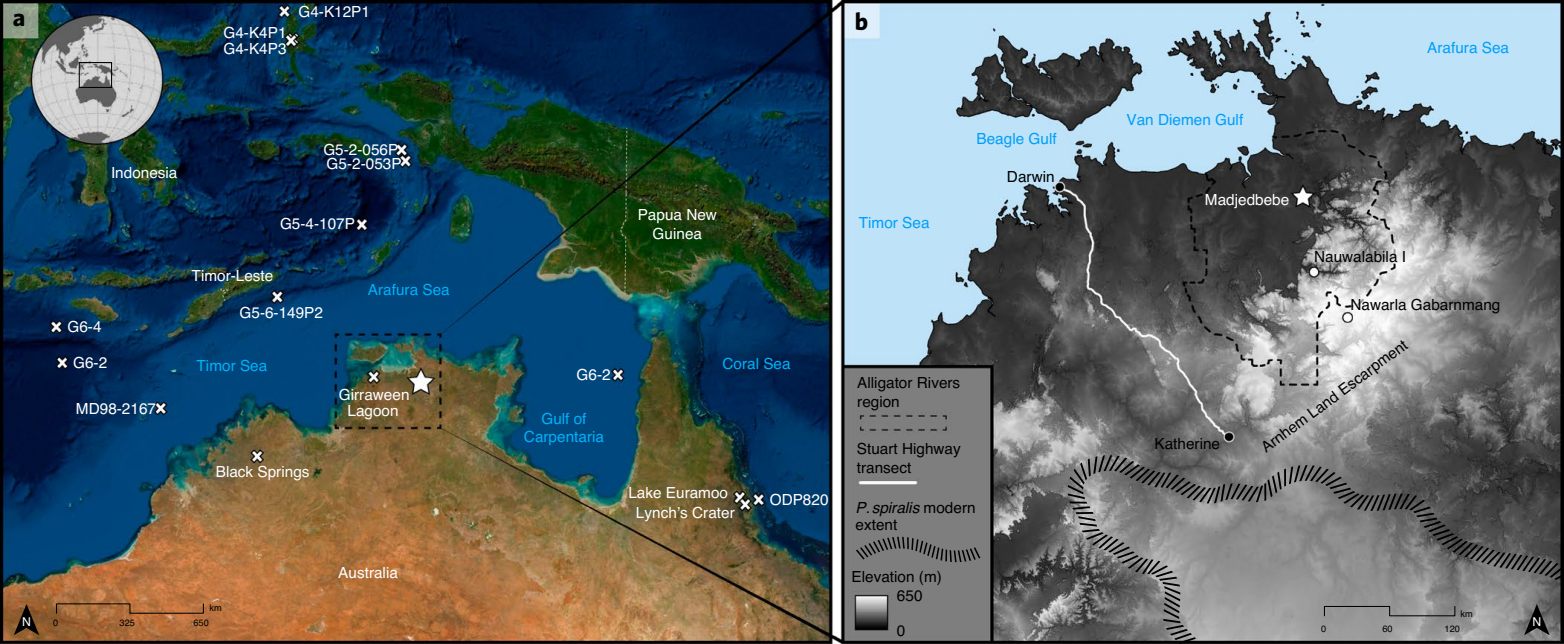
The geographical location of the study area. **a**, A map showing the location of Madjedbebe (star) and all Pleistocene-age environmental records for the monsoonal tropics of northern Sahul (crosses)^29,59–61^. **b**, The location of the modern and archaeological study sites, including Madjedbebe and other early (≥45ka) archaeological sites (white dots), within and near the Alligator Rivers region (dashed line)^1,6,7^; the Stuart Highway transect from Darwin to Katherine (white line); and the modern extent of the southern distribution of *P. spiralis* (slashed line)^62^. Esri, DigitalGlobe, GeoEye, Earthstar, Geographics, CNES/Airbus DS, USDA, USGS, AeroGRID, IGN, and the GIS User Community (**a**); panel **b** adapted with permission from ref. ^63^, Geoscience Australia

Madjedbebe is a large sandstone rockshelter located on Mirarr country in the Alligator Rivers region, northern Australia, with evidence for human occupation from at least 65 thousand years ago (ka) to the present (Fig. 1b)^1^. The site provides insights into the behaviour of the first modern humans to reach Sahul (the combined Pleistocene landmass of Australia, New Guinea and the Aru Islands), including evidence for the early use of hafted edge-ground axes, grinding stones, reflective pigments and a broad diet of plant foods^1,2^. Human use of the site spans several periods of substantial global climate change between Marine Isotope Stages (MIS) 4 and 1, including the last and penultimate glacial stages (MIS2 and MIS4, respectively)^3^, and the formation of the Kakadu wetlands in the late Holocene^4,5^. Documenting palaeoprecipitation and subsequent vegetation and resource changes in the Alligator Rivers region is fundamental to our understanding of the adaptive plasticity of populations entering this region for the first time. However, while there are several early (≥45 ka) archaeological sites from this region^1,6,7^, the best source of palaeoclimate data for this early occupation phase is located ~800 km to its east in the Gulf of Carpentaria (Fig. 1)^8,9^. This limits our ability to accurately study local human–environment interaction in this region.

**Fig. 2:**
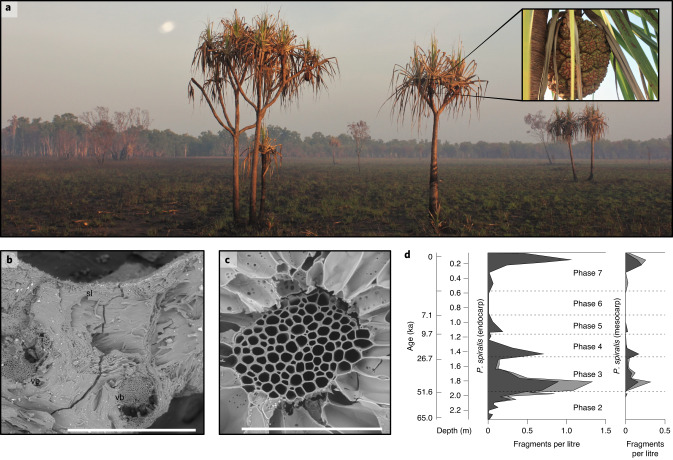
Modern and archaeological *P. spiralis*. **a**, *P. spiralis* trees on the Magela Creek floodplain near Madjedbebe in October 2017, with an inset displaying the cephalium, or aggregate fruit of the tree, comprising ~20 polydrupes (see Supplementary Section 1). **b**, A scanning electron micrograph of an archaeological fragment of *P. spiralis* endocarp from Phase 7 (C3/5). sl, seed locule; vb, vascular bundle. Scale bar, 1 mm. **c**, A scanning electron micrograph of an archaeological fragment of *P. spiralis* mesocarp from Phase 7 (C3/6). Scale bar, 200 µm. See Florin et al.^2^ for a detailed identification proof. **d**, The numbers of fragments of *P. spiralis* endocarp and *P. spiralis* mesocarp recovered per litre of soil floated from Madjedbebe, versus increasing depth (m) and decreasing archaeological phase. The dark grey area indicates the numbers of *P. spiralis* endocarp and mesocarp by litres floated, respectively, and the light grey area indicates the numbers of cf. *P. spiralis* endocarp and mesocarp by litres floated, respectively. The age estimates are based on the modelled mid-point value of the 95% confidence interval for the start date of each phase^1^.

**Extended Data Fig. 1:**
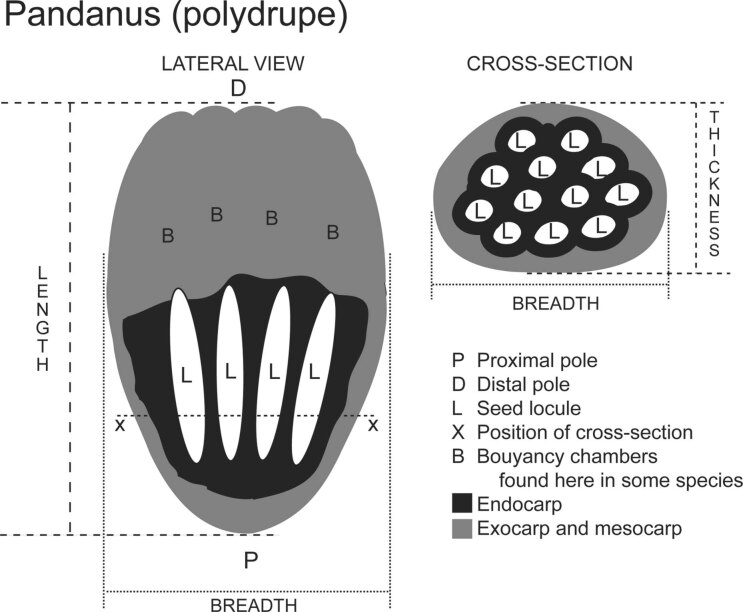
Schematic of a drupe from a polydrupe or syncarpous *Pandanus* spp. Note that portions of both the endocarp and the mesocarp are found archaeologically. Figure adapted from ref. ^2^, Springer Nature Ltd.

In other parts of the world, stable isotope analysis of archaeological plant material has emerged as a promising way of studying changes in past precipitation and temperature in direct association with records of human occupation and cultural change^10–13^. Such work has tended to focus on Eurasian crops and tree species, with limited applicability to other regions. Here we report on the results of stable carbon isotope analysis of modern and archaeological *Pandanus spiralis* endocarp (the tough ‘nutshell’ of the pandanus drupe, comprised of sclerenchyma tissue; Extended Data Fig. 1). Pandanus has been found in archaeological sites across the tropics, including as part of Melanesian and Pacific foraging and agricultural systems^14–17^, and *P. spiralis* was recovered from almost all occupation layers at Madjedbebe (Fig. 2)^2^. We demonstrate that stable carbon isotope analysis of this taxon can provide a reliable palaeoprecipitation proxy, and use it in concert with the analysis of soil stable carbon isotopes, and lithic artefact and exotic raw material discard rates from Madjedbebe, to investigate the relationship between environmental change and human mobility and settlement in the past.

**Fig. 3:**
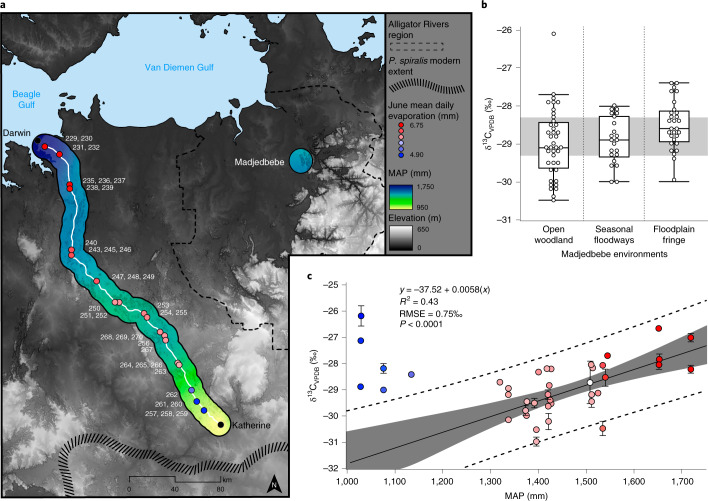
Results of the modern stable carbon isotope analysis. **a**, Samples of *P. spiralis* collected from different locations along the Stuart Highway, between Darwin and Katherine. MAP is calculated from 24 meteorological sites (see Supplementary Section 2) and displayed as a 10-km buffer surrounding the sampling transect, with blue and yellow indicating higher and lower precipitation, respectively. Additionally, we indicate the mean daily evaporation for June by the fill colour of each dot, which reflects the first month of fruiting of the *P. spiralis* drupe^23^. Individual *P. spiralis* trees are indicated by their specimen number (white numbers; Supplementary Table 3). **b**, δ^13^C values of *P. spiralis* collected from different growth environments (floodplain fringe, seasonal floodways, and open forest and woodland vegetation communities) with the same MAP (~1,510 mm yr^−1^), near Madjedbebe in the Alligator Rivers region. The grey horizontal band demarcates the interquartile range of the δ^13^C values of modern *P. spiralis* from the Alligator Rivers region. Boxes show the interquartile range, the midline shows the median value, and the whiskers extend vertically 1.5 times the interquartile range from the end of the box to the furthest datum within that distance. Data beyond that distance are represented individually as points (‘outliers’). **c**, A linear relationship is observed between precipitation and δ^13^C values of *P. spiralis* with 95% confidence intervals (grey area) and 95% prediction intervals (dashed lines) when removing all sites affected by microclimate effects (blue dots with mean evaporation <5 mm per day). Error bars on data points indicate one standard deviation. Note that the modern δ^13^C value for *P. spiralis* collected near Madjedbebe (white dot) falls directly on our best-fit line (sample not used to generate this regression). RMSE, root-mean-square error. The data and R code for this figure are available from ref. ^54^. Panel **a** adapted with permission from ref. ^63^, Geoscience Australia.

**Extended Data Fig. 2:**
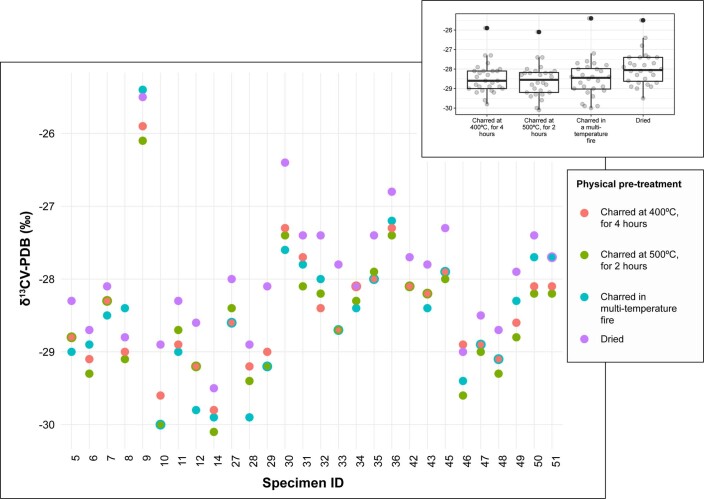
Results of the modern *Pandanus spiralis* charring experiment. Compares the δ^13^C values of endocarp from the same trees prepared with different physical pre-treatment methods. There is a significant difference between the dried and charred δ^13^C values (One-way ANOVA, F = 2.81, df = 111, p = 0.043). This may be caused by the variation in chemical pre-treatment methods used (soxhlet extraction pre-treatment, rather than ABA pre-treatment, was used for the dried specimens; see Methods) or by the process of charring itself. However, there is little difference between the δ^13^C values of the charred specimens (One-way ANOVA, F = 0.12, df = 83, p = 0.883). This is despite the fact that one charring method occurred on an open fire, which reached temperatures > 1300 °C. Therefore, as the archaeological specimens are also charred and have been chemically pre-treated using the same method as the modern specimens, any change in δ^13^C through charring is not expected to affect the results.

To test whether stable carbon isotope discrimination in *P. spiralis* endocarp is a useful proxy for the analysis of past fluctuations in mean annual precipitation (MAP), modern *P. spiralis* drupes were collected from a range of environments in the Northern Territory, and their charred endocarps were analysed for variation in [(^13^C/^12^C)_sample_/(^13^C/^12^C)_standard_] −1 (δ^13^C) values (Fig. 3; see Extended Data Fig. 2 for the results of charring experiments on the δ^13^C values of *P. spiralis* endocarp). Figure 3b depicts δ^13^C variation between different growth environments (floodplain fringe, seasonal floodways, and open forest and woodland environments) found within a 10-km radius from Madjedbebe rockshelter. Figure 3c documents variation in δ^13^C values with rainfall within the modern range of distribution of *P. spiralis*, following a >300-km transect along the Stuart Highway from Darwin to Katherine. Both show a significant correlation between increasing δ^13^C values and increased water availability (Fig. 3b: one-way analysis of variance, *F* = 3.572, d.f. = 2, *P* = 0.032; Fig. 3c: *R*^2^ = 0.43, root-mean-square error = 0.75‰, *P* = <0.001).

These results run counter to many of the observed relationships in C_3_ plants that link declining MAP and drought conditions to increasing δ^13^C values of plant tissues^18,19^. However, these results are in line with expectations of changed C_3_ carbon isotope discrimination in waterlogged environments^20,21^. Today, *P. spiralis* has a wide distribution across northern Australia, where it grows in poorly drained areas of *Eucalyptus*- and *Melaleuca*-dominated vegetation communities. This includes along the edges of swamps and billabongs, the fringes of floodplains, and in seasonally wet areas of open forest and woodland. With more than 90% of all precipitation in northern Australia occurring from November to April^22^, such environments undergo significant seasonal flooding. This means that *P. spiralis* trees are often waterlogged for several months of the year, including at the beginning of their fruit growth season (*P. spiralis* fruits mature from June to October)^23^. The period of waterlogging experienced by *P. spiralis* plants increases both in regions of higher MAP and in environments more likely to experience prolonged inundation (for example, the fringes of floodplains).

**Extended Data Fig. 3:**
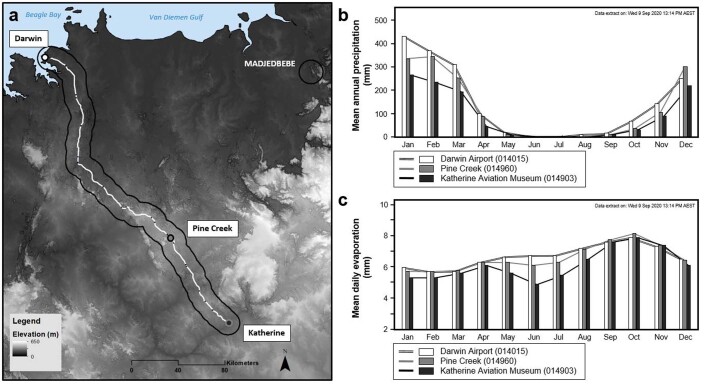
Modern precipitation and evaporation data from Darwin, Pine Creek and Katherine. a) Map of the Stuart Highway transect from Darwin to Katherine (white line), showing proximity to Madjedbebe; b) and c) Mean annual precipitation and daily evaporation data, respectively, from Darwin, Pine Creek and Katherine. Panel a adapted with permission from ref. ^63^, Geosciences Australia, and panel b and c adapted with permission from ref. ^30^, Bureau of Meteorology.

Like other environmental stresses (for example, aridity), waterlogging has been shown to have a negative effect on the photosynthetic rate and, subsequently, the water use efficiency (WUE) of C_3_ plants^20,21^. This is understood to be the result of decreased stomata conductance caused by several factors, including decreased root hydraulic conductance, increased oxygen deficiency and subsequent stomata closure. The increased δ^13^C values evidenced in *P. spiralis* endocarp growing in environments with higher groundwater availability in this study (for example, with higher MAP or on the fringes of floodplains) are, therefore, probably caused by the effect of prolonged wet season waterlogging experienced by these plants. The only exception to this positive correlation is towards the southern extent of the modern *P. spiralis* distribution, where δ^13^C values are higher (that is, WUE is lower; Fig. 3). In terms of precipitation, this is the driest habitat recorded in the transect (~1,150–1,000 mm rainfall per year). However, climate records from this area show significantly reduced evaporation in the early dry season^24^, which we attribute to cold-air drainage from adjacent high ground (that is, the Arnhem Land Escarpment; Fig. 1b). This reduced evaporation permits standing water to remain longer in this area in June, when *P. spiralis* drupes are growing despite the reduced precipitation (Extended Data Fig. 3). As such, the lower WUE evidenced near Katherine is also a factor of increased waterlogging during the growing season of *P. spiralis* drupes. While this means that variability in local topography can cause variability in the effects of MAP on δ^13^C values of *P. spiralis* drupes at a particular location, the fit of δ^13^C values from the direct vicinity of Madjedbebe to the linear relationship observed between precipitation and δ^13^C values along the Stuart Highway transect suggests that Madjedbebe is an ideal site at which to use this proxy (Fig. 3c).

**Fig. 4:**
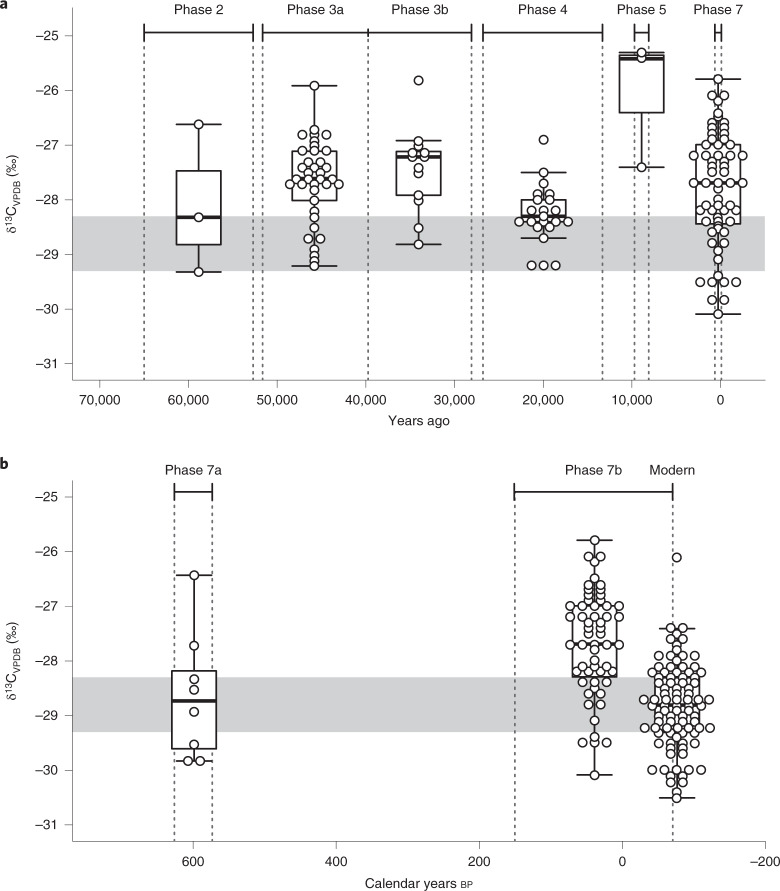
Results of the archaeological isotope analysis. **a**, δ^13^C values of archaeological *P. spiralis* endocarp, describing fluctuations in climate at Madjedbebe over the last 65,000 years. The vertical dotted lines demarcate the temporal boundaries of the different archaeological phases, based on the modelled mid-point value of the 95% confidence interval for the start and end date of each phase^1^. The exception is Phase 7, where AMS radiocarbon mid-point ages for the analysed contexts are used (Supplementary Table 10). The grey horizontal band demarcates the interquartile range of the δ^13^C values of modern *P. spiralis* from near Madjedbebe. **b**, A close-up of δ^13^C values of archaeological and modern *P. spiralis* from Madjedbebe and its close surrounds over the last 700 years. The vertical dotted lines demarcate the temporal boundaries of the archaeological *P. spiralis* samples. The grey horizontal band demarcates the interquartile range of the δ^13^C values of modern *P. spiralis* from near Madjedbebe. Boxes show the interquartile range, the midline shows the median value, and the whiskers extend vertically 1.5 times the interquartile range from the end of the box to the furthest datum within that distance. Data beyond that distance are represented individually as points (‘outliers’). The data and R code for this figure are available from ref. ^54^.

Figure 4 displays the δ^13^C values of the archaeological *P. spiralis* endocarp by archaeological phase from Madjedbebe. Following the Volker-2012a model^25^, these values have been corrected for changes in the isotopic composition of atmospheric CO_2_ (δ^13^CO_2ATM_) and the partial pressure of CO_2_ ($p_{{\mathrm{CO}}_{2}}$) over the past 65,000 years. Variations in δ^13^C values in Fig.4a suggest a fluctuating WUE for *P. spiralis* trees across this period, with decreased WUE during Phase 3 (51.6–28.1 ka), Phase 5 (9.7–8.1 ka) and Phase 7 (about 4 ka to present). Gaps occur in this record in the terminal Pleistocene through to the mid-Holocene owing to decreased recovery of *P. spiralis* endocarp from this period (Fig. 1c), probably due to reduced preservation. Charred plant remains were recovered at a significantly lower frequency from the alkali environment of the mid- to late Holocene midden (Phases 6 and 7) and the carbonate-enriched sediments directly below it (Phases 4 and 5; see Supplementary Fig. 2 in Florin et al.^2^). Figure 4b documents variation on a shorter timescale, depicting variations in the corrected δ^13^C values of the archaeological and modern *P. spiralis* endocarp over the past 700 years, indicating fluctuations in WUE within Phase 7. On the basis of the modern data, decreased WUE in *P. spiralis* is most likely a reflection of waterlogging and, therefore, higher precipitation during Phases 3, 5 and 7. In comparison, Phase 2 (65–52.7 ka) and Phase 4 (26.7–13.2 ka) represent periods of higher WUE and, therefore, decreased precipitation. Interestingly, the δ^13^C values of the modern *P. spiralis* endocarp are the lowest in the sequence (Fig. 4), suggesting that the lowest recorded precipitation is occurring at present.

**Fig. 5:**
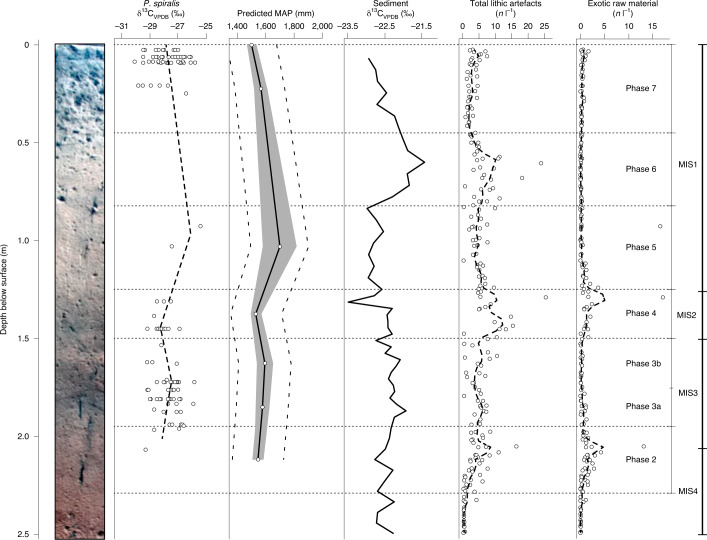
Results of the isotope analysis of *P. spiralis* endocarp, compared to isotope analysis of soil, and stone artefact and exotic raw material discard by depth. From left to right: soil profile; δ^13^C values of archaeological *P. spiralis* endocarp from Square C3 grouped by phase and displayed by mean depth of contexts; predicted MAP at Madjedbebe, based on the mean δ^13^C values of archaeological *P. spiralis* endocarp and the modern relationship between δ^13^C and MAP (Fig. 3c), with 95% confidence interval (grey area) and 95% prediction intervals (dashed lines); δ^13^C of archaeological soil organic matter samples from Square B2, taken at ~5-cm intervals and grouped by phase; number of lithic artefacts per litres floated for Squares E1 to B3 by depth; number of lithic artefacts produced from exotic raw materials per litres floated for Squares E1 to B3 by depth. Dashed lines on the second, fifth and sixth panels are locally estimated scatterplot smoothing (loess) curves. The data and R code for this figure are available from ref. ^54^. Soil profile in the left panel adapted with permission from ref. ^1^, Springer Nature Ltd.

Figure 5 compares the pandanus proxy for palaeoprecipitation, and predicted MAP, with the previously published soil δ^13^C record^1^, corrected for changing δ^13^CO_2ATM_ and $p_{{\mathrm{CO}}_{2}}$, and the discard of lithic artefacts and exotic raw materials at Madjedbebe. The soil δ^13^C record is probably a product of both environmental and anthropogenic influences, deriving from a combination of vegetation growing within and immediately outside the site, vegetation brought in by natural mechanisms (for example, wind and water) and vegetation brought in by humans for a range of reasons, including subsistence, bedding and fuel^26^. This makes its interpretation more complex. In northern Australia, an abundance of C_4_ plants in the environment correlates with seasonal water availability, with a higher abundance of C_4_ grasses occurring in locations where precipitation during the summer monsoon season is high^27,28^. Therefore, despite possible anthropogenic influences, the similar peaks (in Phase 3 and, albeit slightly later, in Phase 6) and troughs (in Phases 2 and 4 and at the end of Phase 7) in both records suggest a general agreement in variation in palaeoprecipitation at the site.

The *P. spiralis* record suggests decreased precipitation during the penultimate (MIS4) and last glacial (MIS2) stages, although the timing and intensity of the latter is in part masked by poor preservation of archaeological *P. spiralis* endocarp in the terminal Pleistocene/early Holocene. The δ^13^C values from the soil record suggest that peak aridity at Madjedbebe may have occurred at the height of MIS2 or the Last Glacial Maximum, about 22–18 ka, in the latter half of Phase 4 (Fig. 5). Precipitation then increased in the early and mid-Holocene, consistent with the early Holocene climatic optimum (about 10–6 ka)^29,30^. Late onset of aridity in tropical northern Australia is corroborated by other palaeoenvironmental records, which show river flows increasing after 30 ka and declining only from 17 to 10 ka (see Supplementary Section 8 for further discussion of the Madjedbebe proxy in comparison to other northern Australian palaeoenvironmental records)^31^.

As the lowest WUE of *P. spiralis* is recorded in the modern record, and there is a general correlation between the soil and pandanus δ^13^C records, rainfall in the vicinity of Madjedbebe may have rarely, if ever, fallen below present levels (~1,500 mm yr^−1^) in the past 65,000 years. This attests to moderate precipitation in this region, even throughout the drier stages of MIS4 and MIS2. Indeed, evidence for plant food exploitation and fuel use in Phase 2 at Madjedbebe demonstrates that people had access to a range of vegetation communities during early occupation (MIS4), including freshwater and monsoon vine forest patches^2,32^. The same is probably also true of the Last Glacial Maximum and terminal Pleistocene. Our results indicate the severity of glacial stages, and their effects on local vegetation during this period may be overestimated in this region. This is unsurprising as modelling studies (for example, Yan et al.^33^) demonstrate that increased seasonal contrasts during the Last Glacial Maximum enhanced the dynamic forcing of the monsoon in northern Australia. This is partially offset by the reduction in available moisture in the atmosphere, but overall a minor increase in monsoon strength is inferred. Further, this is supported archaeologically by the peaks in artefact discard at Madjedbebe during dry phases (Fig. 5c), which suggest increased use of the site during these phases, and are consistent with earlier arguments that human occupation intensified in the Alligator Rivers region during the Last Glacial Maximum owing to its relatively wet environment^34^. Peaks in exotic raw materials during dry phases (Fig. 5d) also indicate that people were probably more mobile at these times, with decreased abundance of resources requiring them to extend their foraging range and possibly their social networks^35,36^.

## Discussion

As there are little data for palaeoprecipitation levels during MIS4 across northern Australia, let alone in the Alligator Rivers region, this proxy provides valuable environmental context for early settlement of the continent. Modern human populations entering this region 65,000 years ago would have experienced a relatively dry, but not arid, environment, with small pockets of monsoon vine forest and freshwater vegetation available on the Alligator Rivers lowlands^2,32^. As climate ameliorated in Phase 3, monsoon vine forest environments probably increased in the vicinity of Madjedbebe^37^. At this time, people frequenting the rockshelter probably reduced their residential range, using fewer exotic raw materials for artefact manufacture. They also ceased the production of certain distinctive lithic artefact types, including thinning flakes, convergent flakes and discoidal core technology^1^. It is during this phase that human occupation is evidenced in other parts of Sahul^17,38–42^.

The efficacy of pandanus δ^13^C as a proxy for palaeoprecipitation is a potential game-changer for palaeoecological and archaeological work in Australia, Melanesia and the Pacific. As in other regions, palaeoprecipitation records from archaeological sites are often lacking. This has meant that the links between climatic changes, human modification of landscape and human behaviour often refer to off-site and distant lake or marine records that may be unreliable indicators of local conditions. Across the tropics, where pandanus and other tree nuts are ubiquitous and well preserved, the isotopic analysis of such food plant remains offers a method for the development of context-specific proxies for past environment and land management, and their comparison to cultural, social and technological changes.

## Methods

### Charring experiment for modern *P. spiralis*

To test the effect of carbonization on the δ^13^C value of *P. spiralis*, carbon isotope analysis was carried out on the nutshell of 108 drupes from 27 *P. spiralis* trees under different states of physical pre-treatment. Four drupes from each tree were analysed, each following a different method of physical pre-treatment: dried; charred for 4 h at 400 °C; charred for 2 h at 500 °C; and charred in a multi-temperature open fire. The controlled charring experiments were performed in a low-oxygen state in a kiln, following the protocol set out in Fraser et al.^43^. Open-fire conditions exceeded 1,300 °C, covering the maximum range of ethnographic and experimental hearths^44^.

In line with the archaeological specimens, samples also underwent chemical pre-treatment. The charred samples underwent ABA pre-treatment. This procedure consisted of an acid treatment (10 ml of 2 M HCl at 60 °C for 2 h), two base treatments (10 ml of 0.25% NaOH at room temperature for 30 min) and another acid treatment (10 ml of 2 M HCl at room temperature for 2 h). The sample was rinsed three times with Milli-Q water between treatments and six times following the final acid treatment, before it was dried in an oven at 60 °C overnight and powdered with a glass rod.

The dried samples were milled and then underwent α-cellulose pre-treatment^45^. This procedure consisted of a Soxhlet extraction treatment (2:1 cyclohexane–ethanol mixture for 6 h, ethanol for 6 h, Milli-Q water for 6 h), a lignin extraction treatment (NaClO_2_ oxidation (15 g l^−1^) under acidified conditions (pH of ~3) at 60 °C for 2 h), a cellulose extraction treatment (12% NaOH at 60 °C under nitrogen gas, and then 7% NaOH at 60 °C under nitrogen gas) and an acid treatment (2 M HCl at room temperature for 1 h). The sample was then dried in an oven at 60 °C overnight.

The results of this experiment are reported in Extended Data Fig. 2.

### Rainfall and environment for modern *P. spiralis*

Stable carbon isotope analysis was carried out on the endocarp of 201 modern *P. spiralis* fruits. These fruits came from two areas: Mirarr country near Madjedbebe, Alligator Rivers region; and the edge of the Stuart Highway, between Darwin and Katherine (Figs. 1 and 3). The former allowed for the analysis of variation in the δ^13^C values of *P. spiralis* nutshell from environments local to Madjedbebe, including floodplain fringe, seasonal floodways, and open forest and woodland environments. The latter allowed for the analysis of variation in the δ^13^C values of *P. spiralis* nutshell across different mean annual rainfall zones, ranging from ~1,700–1,600 mm yr^−1^ in Darwin to ~1,150–1,000 mm yr^−1^ in Katherine. Supplementary Table 3 gives details of the site locations, collection dates, MAP, mean daily evaporation and habitat of each of the *P. spiralis* analysed.

After the initial study examining the effect of carbonization on the δ^13^C value of *P. spiralis*, all modern samples were charred at 500 °C for 2 h before analysis and then cut into pieces with a scalpel. The temperature of 500 °C was chosen as it is a commonly prescribed combustion temperature for loss-on-ignition protocols for plant materials^46^. In line with the archaeological specimens, samples also underwent ABA pre-treatment.

### Modern climate

To test whether the ^13^C isotopic signature of *P. spiralis* drupes are directly related to water availability, we evaluated two major contributors to the regional water budget, precipitation and evaporation, and produced regional interpolations. All data from the Northern Territory were collected from the Bureau of Meteorology online data page (http://www.bom.gov.au/climate/averages/tables/ca_nt_names.shtml)^24^. MAP was determined from all collected measurements of daily total precipitation during the full observation time utilizing standard rain gauges. Mean daily evaporation was determined using all data collected for the month of June from a class A evaporation pan. June daily evaporation was selected because *P. spiralis* fruits from June to October^23^, with a large amount of their biomass laid down in June (authors’ own observation). Consequently, the ^13^C isotopic signature preserved within the drupe should reflect this interval.

To create regional precipitation and evaporation maps from meteorological sites, we utilized inverse-distance-weighted (IDW) interpolation. This was chosen as stations along our transect were sparsely available. IDW is a deterministic spatial interpolation technique that assumes spatially known data are strongly correlated to distance such that the local influences of neighbouring sites diminish with increasing distance^47^. This technique is particularly useful when datasets are too small to utilize more complex interpolation methods (that is, kriging, spline or multiple regression techniques)^48^. However, IDW is sensitive to site clustering and is optimized when samples are evenly spaced. Therefore, in locations with multiple meteorological stations we selected only one site for inclusion in our analyses. Sites were chosen on the basis of the proximity to the areas of interest and the completeness of climate records, specifically over the last decade.

In total, we used 24 precipitation stations and 12 evaporation stations for our analyses (Supplementary Figs. 4 and 5 and Supplementary Tables 1 and 2). Each meteorological station location was placed in ArcGIS (version 10.6) software and projected to the GDA-94 coordinate system. In the ArcGIS Geostatistical Analyst tools, we selected IDW. For both precipitation and evaporation, we elected to use simple interpolation parameters in our model to avoid bias outcomes and to ensure reproducible results. For this reason, we selected a standard neighbour with one sector and equal major and minor semi-axes such that all unknown sites had equally weighted predictions in all directions. The power value of two (*p* = *2*, scaling factor that determines the influence of distance) places a higher weight on closer locations because climate gradients are strong along our transect. We determined the predicted precipitation and evaporation values from all available meteorological stations at a 0.25-km^2^ resolution for the entire study area. Data were extracted for each sample location using the Spatial Analyst tool Extract Multi Values to Points.

### Archaeological *P. spiralis*

Charred plant macrofossils were recovered from all levels of occupation at Madjedbebe via flotation^1,2^. Archaeobotanical analysis was carried out on the flot and heavy residue of all excavated hearths, and 100% of the Pleistocene and 50% of the Holocene spits from a 1 × 1-m sediment column (Square C3/C2). Carbon isotope analysis was carried out on all identified specimens of polydrupe *Pandanus* sp. endocarp that were >2 mm in size (Supplementary Table 4). These specimens were determined to be fragments of *P. spiralis* both as they were found in conjunction with more fragile *P. spiralis* mesocarp fragments (Fig. 2b–d); and as Madjedbebe is a lowland site, approximately 10 km from the elevated sandstone plateaus where the other northern Australian polydrupe species, *P. basedowii*, occurs.

Many of the charred plant macrofossils recovered from Madjedbebe have been shown through previous pre-treatment for accelerated mass spectrometry (AMS) radiocarbon dating to be significantly degraded^49^, probably owing to the replacement of charcoal by humic substances over time via a process of oxidative degradation^50^. Therefore, following standard procedure, all specimens underwent ABA pre-treatment to remove any humic, fluvic or carbonate contaminants before analysis^51^. This treatment was the same as that performed on modern samples. However, as many of the archaeological samples were significantly degraded, they required repetitions of the base stage of pre-treatment to be carried out, in some cases up to 16 times. Between base treatments, the specimens were washed once with Milli-Q water, allowing for the examination of the clarity of the sample and, therefore, the quantity of contaminants still present. Once samples were found to be clear, they underwent a final base treatment before moving to the second stage of acid treatment. Of the 244 archaeological samples analysed, 104 dissolved during pre-treatment (Supplementary Table 4). As δ^13^C measurements are less sensitive to contamination than ^14^C measurements and many of the archaeological specimens dissolved during pre-treatment, we recommend that future studies test the necessity of ABA pre-treatment for stable isotope analyses.

### Stable carbon isotope analysis

All modern and archaeological samples were weighed (~30–60 µg) into tin capsules for analysis. They were then analysed across 37 runs on an Elementar VarioMICRO Elemental Analyser and an IsoPrime Continuous-Flow Isotope Ratio Mass Spectrometer at the Australian Nuclear Science and Technology Organisation. The δ^13^C results were normalized to an International Atomic Energy Agency reference material, IAEA C8, with a consensus value of δ^13^C_VPDB_ = −18.31‰ (refs. ^52,53^), where VPDB represents Vienna PeeDee Belemnite, and analysed with the commercial reference standards High Organic Sediment Standard OAS (Elemental Microanalysis, catalogue no. B2151) and Protein Standard OAS (Elemental Microanalysis, catalogue no. B2155) as quality control references. Supplementary Tables 5–9 list the results for the standards used. Unless otherwise stated (Supplementary Tables 3 and 4), all results listed are the mean of repeat measurements with the standard deviation of the replicate analyses less than or equal to ±0.3.

### Soil analysis

A 2-g sub-sample of sediment from selected contexts (see Supplementary Section 7) was ground to a fine powder and treated with 2 M HCl for 24 h to remove inorganic carbon. National Bureau of Standards NBS-19 was used to normalize the data to the VPDB scale. Analytical precision for replicate measurements of δ^13^C in NBS-19 was ±0.2‰ (2*σ*). The results of this analysis were previously reported in Clarkson et al.^1^.

### Adjustment of δ^13^C for changes in past δ^13^CO_2ATM_ and $p_{{\mathrm{CO}}_{2}}$

To compare the archaeological dataset with the modern isotopic analysis presented in this paper, all archaeological samples were corrected for changes in both δ^13^CO_2ATM_ and $p_{{\mathrm{CO}}_{2}}$. This was carried out using a series of offsets calculated for each phase following the Volker-2012a model^25^. Supplementary Table 4 lists both the original and adjusted δ^13^C values.

The soil δ^13^C, initially reported in Clarkson et al.^1^, was also corrected following the Volker-2012a model^25^. Both the original and adjusted δ^13^C values are available from ref. ^54^.

### Lithic analysis

All analysed stone artefacts from Squares E1 to B3 >3 mm in size were counted and identified to raw material type. The three rows closest to the back wall of the site were chosen as they incorporate and surround the flotation squares (C2 and C3). Raw materials not locally found include several varieties of exotic quartzite, silcrete, chert, Oenpelli dolerite, hornfels, basalt, non-local sandstone, a fine-grained vesicular silicate and rock crystal quartz. Although many of these raw materials are of unknown provenance, some are known to be from quite distant sources (such as silcrete and Oenpelli dolerite).

### AMS radiocarbon dating

Three charcoal samples from C3/4, C3/5 and C3/7 were identified and pre-treated using the ABA method. The pre-treated samples were combusted and then converted to graphite^55^. Radiocarbon analysis was carried out using the VEGA AMS Facility at ANSTO^56^. The radiocarbon results were converted to calendar ages using the SHCal13 data^57^ and the OxCal program^58^.

The AMS radiocarbon dates are reported in Supplementary Table 10.

### Reporting Summary

Further information on research design is available in the Nature Research Reporting Summary linked to this article.

## Supplementary information

Supplementary Information Supplementary Notes 1–8, Figs. 1–5, Tables 1–10 and references.

Reporting Summary

Peer review information

## Data Availability

All elements necessary to allow interpretation and replication of the results, including full datasets, are provided in the Supplementary Information. R code and additional data for Figs. 3–5 are available from ref. ^54^. Archaeobotanical material analysed in this study will be kept in the Archaeology Laboratories of The University of Queensland until 2021. It will then be deposited in a Gundjeihmi Aboriginal Corporation keeping place. The material will be accessible upon request from Gundjeihmi Aboriginal Corporation (gundjeihmi@mirarr.net). The language, images and information contained in this publication include reference to Indigenous knowledge including traditional knowledge, traditional cultural expression and references to biological resources (plants and animals) of the Mirarr people. The source Indigenous knowledge is considered “Confidential Information”; traditional law and custom applies to it and the Mirarr people assert copyright over it in addition to any copyright in the complete work. Any Mirarr-related language, images and information are published with the consent of Gundjeihmi Aboriginal Corporation as the representative of the Mirarr people for the purposes of general education purposes. No further use and absolutely no commercial use is authorized without the prior consent and agreement of the Mirarr people. Please contact Gundjeihmi Aboriginal Corporation to request permission to refer to any Indigenous knowledge in this publication.
