# Supplementary material for: Pandanus nutshell generates a palaeoprecipitation record for human occupation at Madjedbebe, northern Australia
Source: Nat Ecol Evol. 2021 Jan 25;5(3):295–303. doi: 10.1038/s41559-020-01379-8 (PMC7929916; doi:10.1038/s41559-020-01379-8)
Supplement: Supplementary file 1 — Supplementary Notes 1–8, Figs. 1–5, Tables 1–10 and references. [file 41559_2020_1379_MOESM1_ESM.pdf]

nature research

https://doi.org/10.1038/s41559-020-01379-8

Supplementary information

Pandanus nutshell generates a palaeoprecipitation record for human occupation at Madjedbebe, northern Australia

In the format provided by the authors and unedited

Supplementary Information

SI Section 1: *Pandanus spiralis*

Supplementary Figure 1: *P. spiralis* tree and S.A.F. on the edges of the Magela Creek floodplain near Madjedbebe

Supplementary Figure 2: Figure 2: *P. spiralis* trees on a seasonal floodway near Madjedbebe

Supplementary Figure 3: *P. spiralis* tree and S.A.F. in an open woodland environment near Madjedbebe

SI Section 2: Environmental data

Supplementary Figure 4: Location of the data collection stations used to compile modern precipitation data for this study

Supplementary Table 1: Meteorological stations used for modern precipitation

Supplementary Figure 5: Location of the data collection stations used to compile modern evaporation data for this study

Supplementary Table 2: Meteorological stations used for modern precipitation

SI Section 3: Modern *Pandanus spiralis* samples

Supplementary Table 3: Modern *P. spiralis* samples

SI Section 4: Archaeological *Pandanus spiralis* samples

Supplementary Table 4: Archaeological *P. spiralis* samples

SI Section 5: Isotope standards

Supplementary Table 5: Isotope standards: SI3790-SI3949

Supplementary Table 6: Isotope standards: SI4396-SI4443

Supplementary Table 7: Isotope standards: SI5263-SI5408

Supplementary Table 8: Isotope standards: SI 5616-SI5821

Supplementary Table 9: Isotope standards: SI5856-SI6124

SI Section 6: AMS radiocarbon dates

Supplementary Table 10: AMS radiocarbon dates

SI Section 7: Soil samples

SI Section 8: Comparison of the Madjedbebe palaeoprecipitation proxies to other Australian environmental records

Supplementary References

SI Section 1: *Pandanus spiralis*

There are over 600 species of *Pandanus* (Family: Pandanaceae) found across the tropics, from West Africa eastwards to the Pacific Islands[1]. They form a diverse genus of palm-like plants with a thickened wood-like stem, a crown of strap-shaped leaves, and, in most cases, prop roots. They are variable in form, with some species only reaching 2m in height (e.g. *P. herbaceous*, *P. toei*), and others, such as the tall arboreous *P. antarensis* of the New Guinea Highlands, reaching up to 30m in height. In the Northern Territory of Australia, there are three species of *Pandanus*, two lowland species, *P. spiralis* and *P. aquaticus*, and one species only found on sandstone escarpment, *P. basedowii*[2]. All of these species are used by Aboriginal groups in this region for a range of activities, including for food, fibrecraft and medicine, and as fire-carriers (MN, DjDj)[3].

P. spiralis, the species recovered archaeologically at Madjedbebe, are omnipresent in the Alligator Rivers region, growing in poorly drained areas. This includes along the margins of floodplains (Fig.1), the banks of swamps, billabongs and seasonal floodways (Fig. 2), and in seasonally inundated areas of open forest and woodland (Fig. 3). *P. spiralis* grow from 2m to >10m in height. Their woody stems are covered in tight, spirally arranged, leaves, armed with small spines[4,5]. Dead leaves may remain on the tree, creating a tough skirt. However, they are more often burnt off leaving a spiral pattern on the trunk, which the species is named after. Unlike *P. aquaticus* and *P. basedowii*, *P. spiralis* trees only sometimes grow prop roots[2]. Prop roots are hypothesised to be adventitious in pandanus, and other plants, for their structural integrity; ready supply of water and nutrients to above-ground stems; and ready supply of oxygen to below-ground roots, especially in inundated, anoxic soil environments[6].

P. spiralis are highly-prized for their leaves, which are dried, stripped and, often, died for basketry and other fibrecraft, and for their kernels, which are rich in fat (44-50%) and protein (20-34%; MN, DjDj)[7-9]. The basic fruiting unit in the *Pandanus* genera is a drupe, namely a fruit with a fleshy/soft outer layer (including the exocarp and mesocarp) and hard inner layer (the endocarp or ‘nutshell’) surrounding the seed(s) or kernel(s) (see Extended Data 1). Species in which the drupes are predominantly single-seeded or unicarpellated are referred to as monodrupes, and species in which the drupes consist of several fused seed locules are referred to as polydrupes. *P. spiralis* is a polydrupe species.

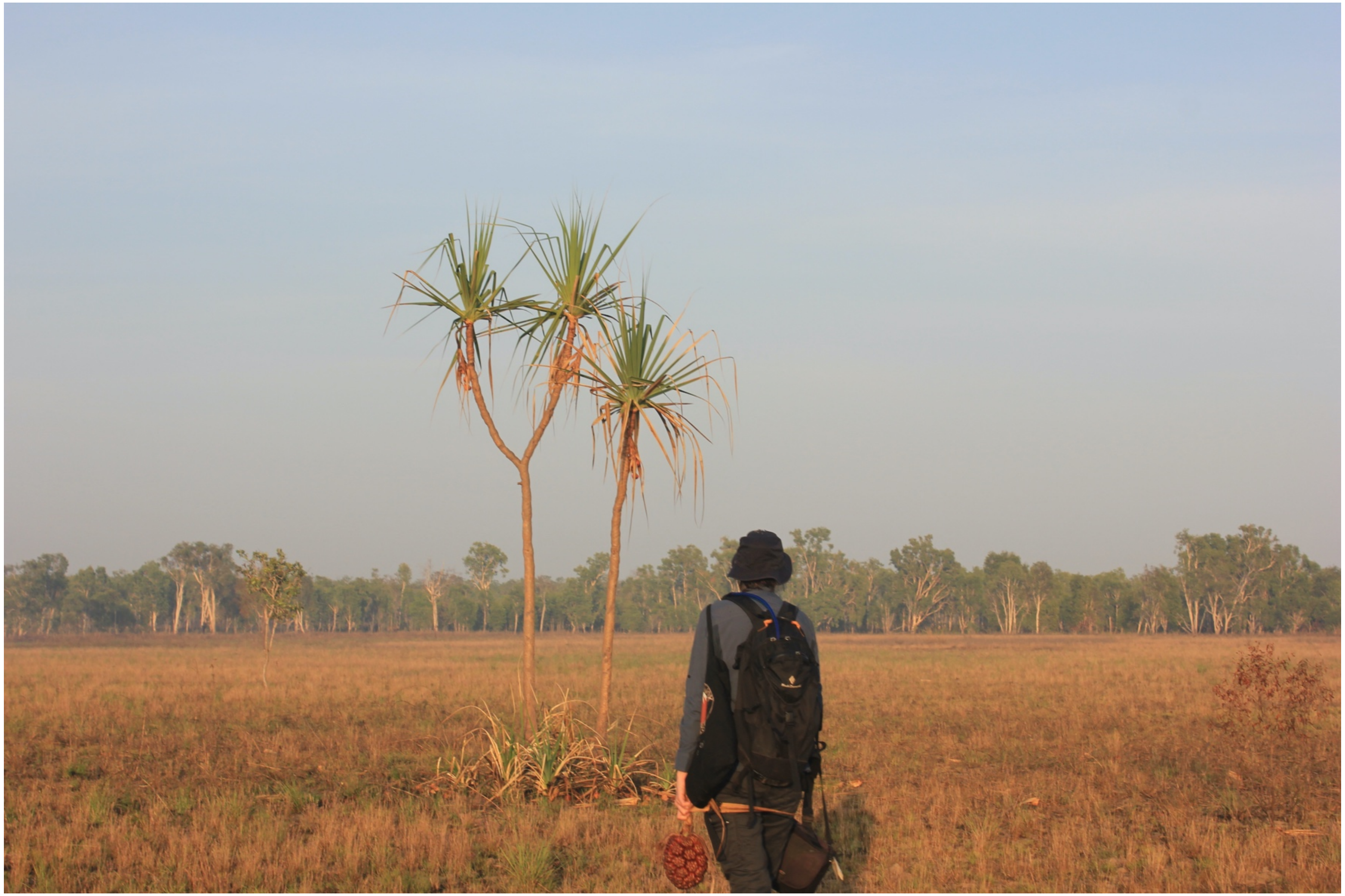

Supplementary Figure 1: *P. spiralis* tree and S.A.F. on the edges of the Magela Creek floodplain near Madjedbebe, October 2017. Note the band of *Melaleuca* species trees in the distance.

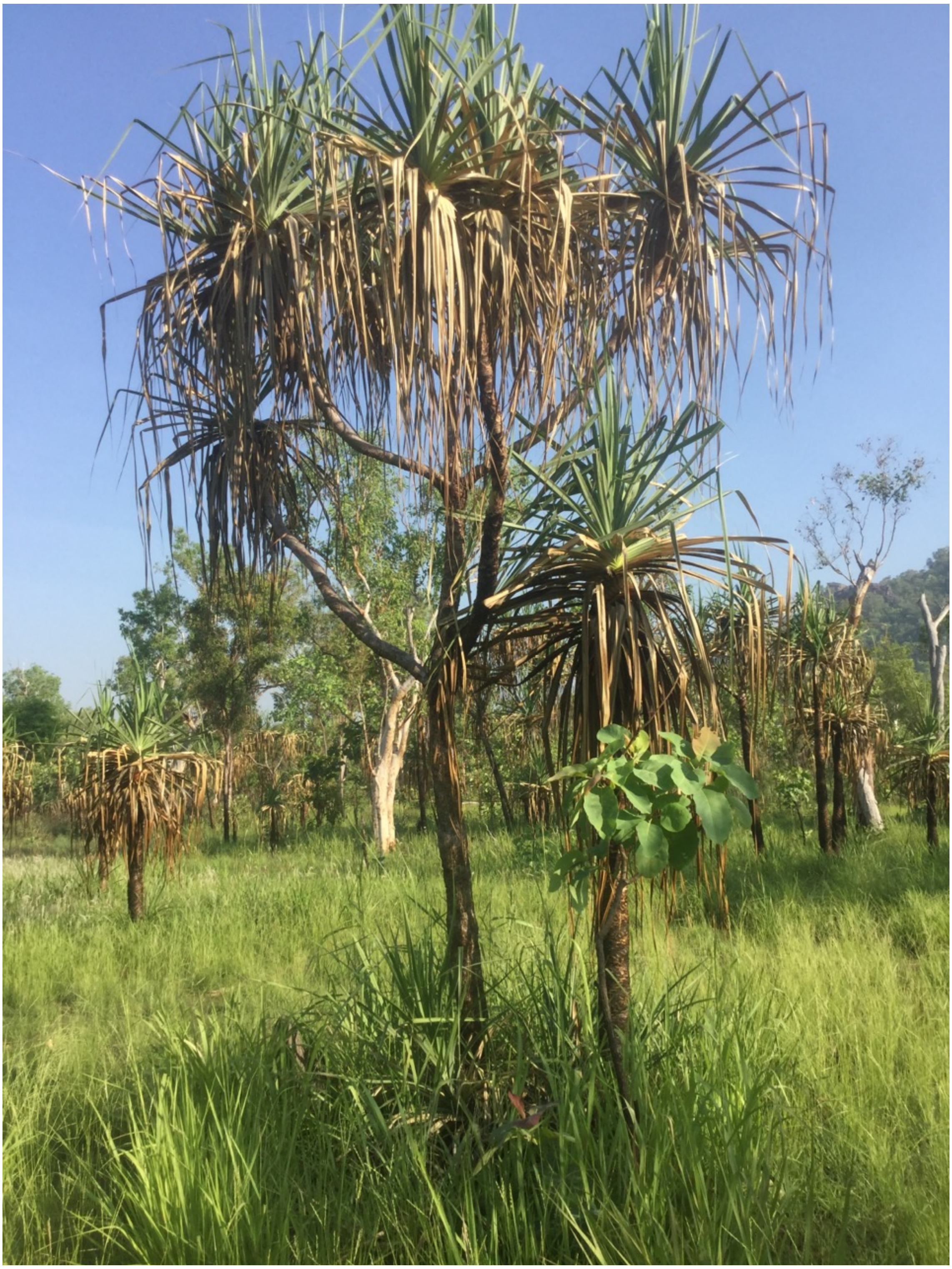

Supplementary Figure 2: ***P. spiralis*** **trees on a seasonal floodway near Madjedbebe**, following early rainfall in October 2016.

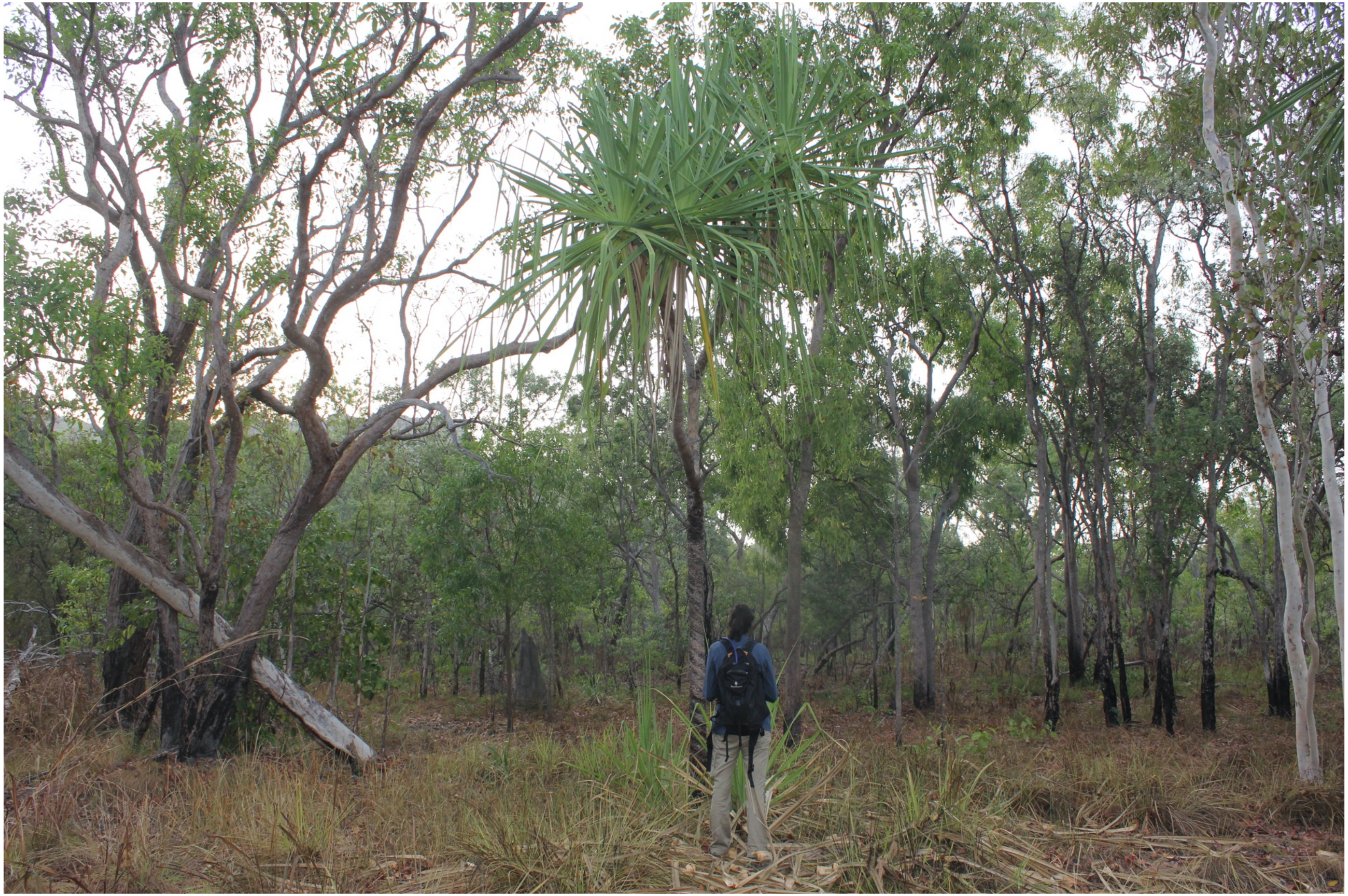

Supplementary Figure 3: *P. spiralis* tree and S.A.F. in an open woodland environment near Madjedbebe, October 2017.

SI Section 2: Environmental data

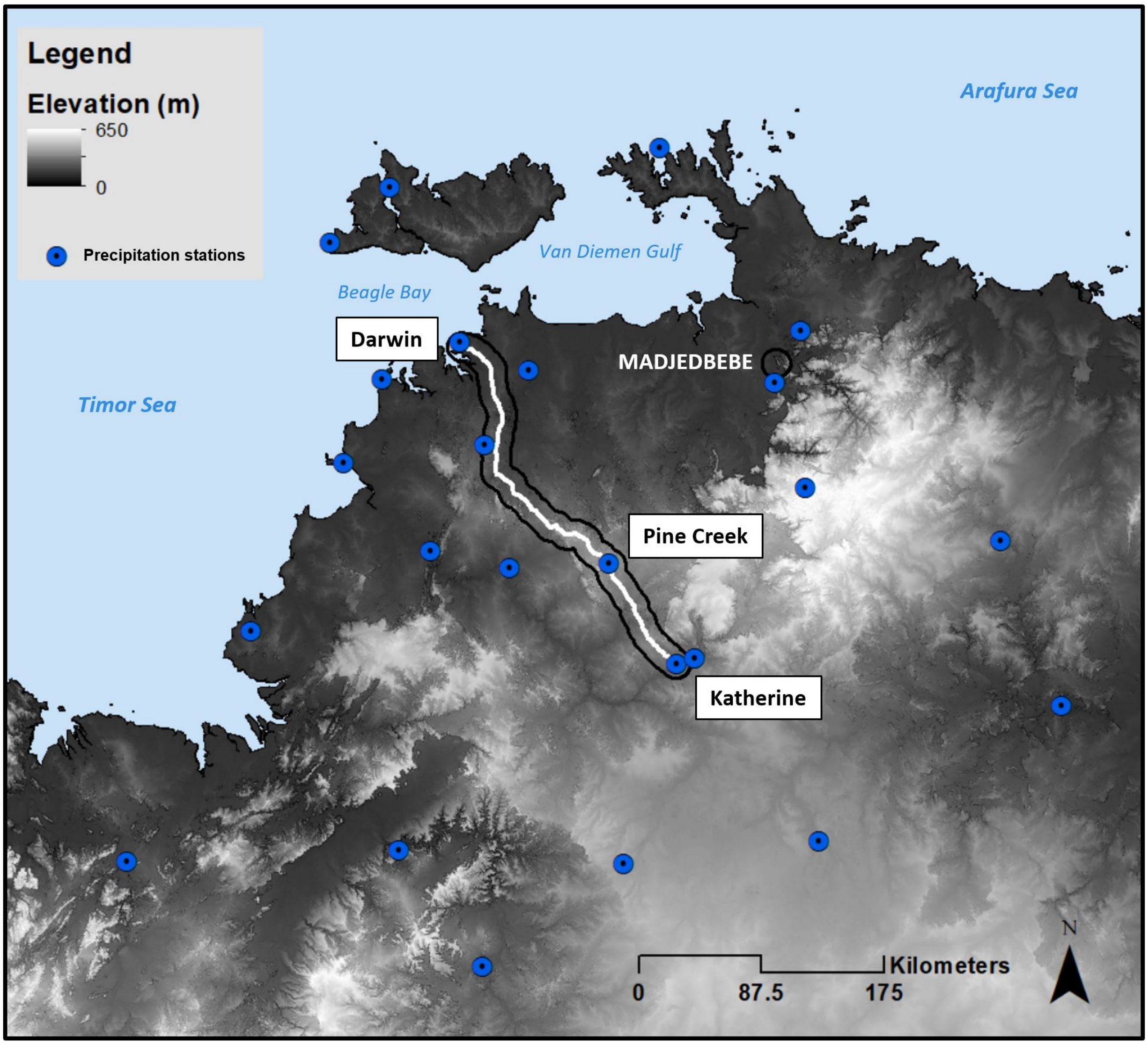

Supplementary Figure 4: Location of the data collection stations used to compile modern precipitation for this study, showing proximity to the Stuart Highway transect and Madjedbebe, elevation data from Hutchinson et al.[10] © Commonwealth of Australia (Geoscience Australia) 2008.

Supplementary Table 1: Meteorological stations used to determine modern mean annual precipitation for this study, and proximity to the Stuart Highway transect and Madjedbebe.

Weather Station	*Station Number*	*Latitude*	*Longitude*	*Observation Time*	*Mean Annual Precipitation (mm)*
Batchelor Airport	014272	13.05 °S	131.03 °E	1994-2020	1535.0
Black Point	014153	11.15 °S	132.14 °E	1965-2020	1263.9
Bulman	014627	13.67 °S	134.34 °E	2002-2020	1043.7
Central Arnhem Plateau	014909	13.33 °S	133.09 °E	2003-2020	1131.7
Channel Point	014253	13.17 °S	130.12 °E	1990-2020	1822.5
Darwin Airport	014015	12.42 °S	130.89 °E	1941-2020	1721.1
Delamere Weapons Range	014949	15.74 °S	131.92 °E	1997-2020	854.4
Douglas River Research Farm	014901	13.83 °S	131.19 °E	1968-2020	1245.8
Dum In Mirrie Airstrip	014277	12.64 °S	130.37 °E	1994-2020	1760.6
Jabiru Airport	014198	12.66 °S	132.89 °E	1971-2020	1553.7
Katherine Council	014903	14.46 °S	132.26 °E	1873-2020	967.8
Kimberly Res Station	002014	15.65 °S	128.71 °E	1944-2010	824.9
Larrimah	014612	15.57 °S	133.21 °E	1952-2020	851.4
Mango Farm	014938	13.74 °S	130.68 °E	1980-2015	1434.6
Middle Point	014090	12.58 °S	131.31 °E	1957-2019	1433.5
Ngukurr	014609	14.73 °S	134.73 °E	1910-2012	774.3
Oenpelli	014042	12.32 °S	133.06 °E	1910-2013	1406.2
Pine Creek Council	014960	13.82 °S	131.83 °E	2000-2016	1423.1
Pirlangimpi Airport	014142	11.40 °S	130.42 °E	1963-2020	1989.3
Point Fawcett	200731	11.76 °S	130.03 °E	1995-2020	1572.9
Port Keats Airports	014948	14.25 °S	129.53 °E	1997-2020	1289.0
Timber Creek	014850	15.66 °S	130.48 °E	1981-2020	959.0
Tindal RAAF	014272	14.52 °S	132.38 °E	1969-2020	1074.1
Victoria River Downs	014825	16.40 °S	131.01 °E	1885-2020	648.9

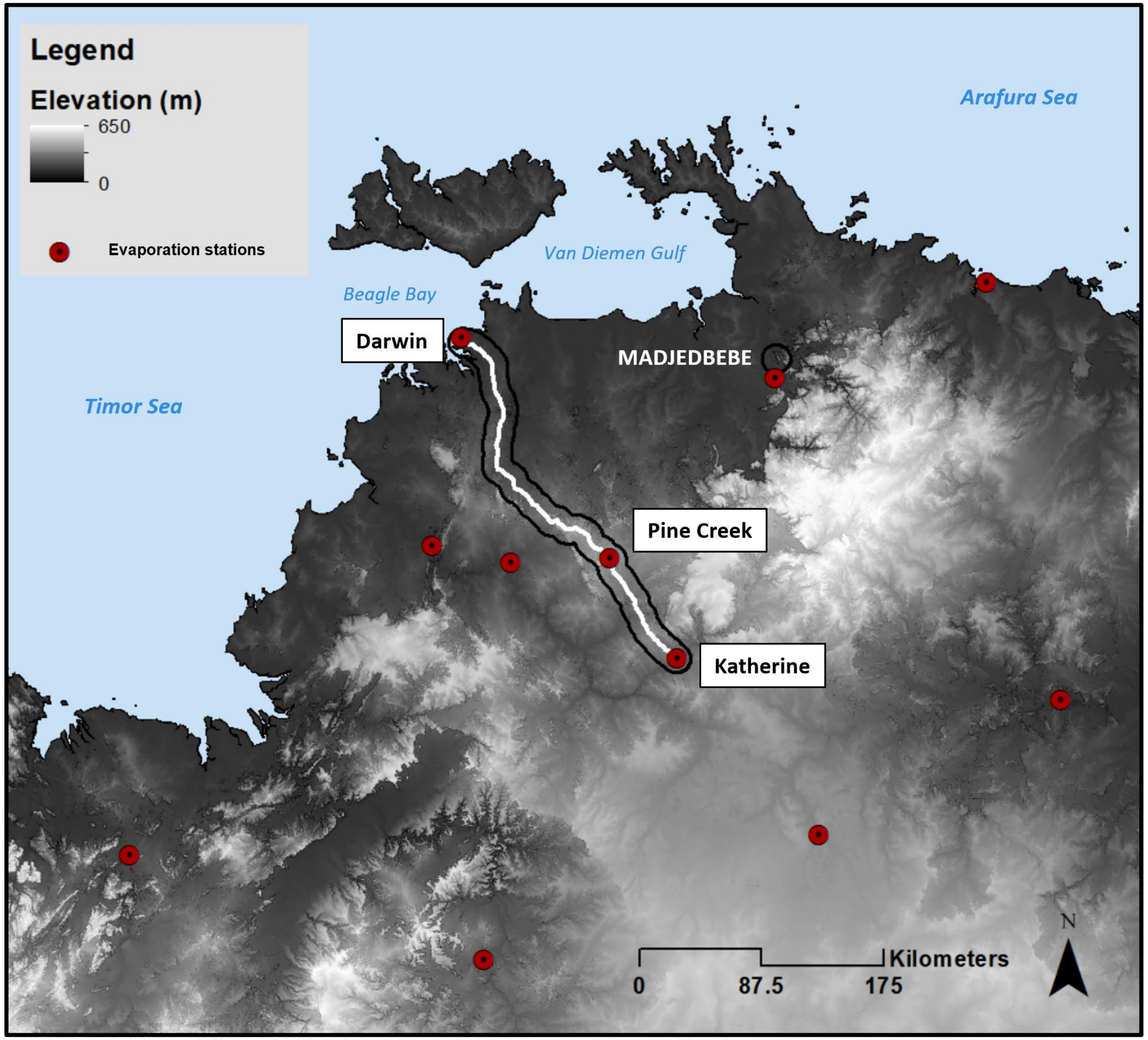

Supplementary Figure 5: Location of the data collection stations used to compile modern evaporation data for this study, showing proximity to the Stuart Highway transect and Madjedbebe, elevation data from Hutchinson et al.[10] © Commonwealth of Australia (Geoscience Australia) 2008.

Supplementary Table 2: Meteorological stations used to determine modern mean daily evaporation for this study, and proximity to the Stuart Highway transect and Madjedbebe.

Weather Station	*Station Number*	*Latitude*	*Longitude*	*Observation Time*	*Mean Daily Evaporation for June (mm)*
Darwin Airport	014015	12.42 °S	130.89 °E	1957-2020	6.8
Douglas River Research Farm	014901	13.83 °S	131.19 °E	1968-2016	6
Grove Airport	014508	12.27° S	136.82° E	1966-2017	5.2
Jabiru Airport	014198	12.66 °S	132.89 °E	1973-1990	6.7
Katherine Aviation Museum	014903	14.44 °S	132.27 °E	1999-2011	4.9
Kimberly Res Station	002014	15.65 °S	128.71 °E	1965-2010	6.1
Larrimah	014612	15.57 °S	133.21 °E	2001-2012	5.4
Mango Farm	014938	13.74 °S	130.68 °E	1980-2015	5.8
Maningrida	014400	12.05 °S	134.23 °E	1967-2007	4.3
Ngukurr	014609	14.73 °S	134.73 °E	1964-2012	5.3
Pine Creek Council	014960	13.82 °S	131.83 °E	2000-2011	6.1
Victoria River Downs	014825	16.40 °S	131.01 °E	1993-2020	5.3

SI Section 3: Modern *Pandanus spiralis* samples

Supplementary Table 3: Modern *P. spiralis* isotope samples; MAP: Mean annual precipitation, MDE: Mean daily evaporation.

ANSTO Code	Specimen Number	Date collected	Locality	Longitude	Latitude	Vegetation	MAP (mm)	MDE (mm)	Physical pre-treatment	Chemical pre-treatment	δ13C V-PDB (‰)
**SI3790**	5a	8/10/15	Arnhem Highway, ERA Mining Lease	12º28'51.23"S	132º54'02.57"E	Open forest and woodland	1479.67	6.63538	Charred in a multi-temperature fire	ABA	-29.0
**SI3791**	6a	8/10/15	Arnhem Highway, ERA Mining Lease	12º29'01.28"S	132º53'58.51"E	Open forest and woodland	1479.67	6.63538	Charred in a multi-temperature fire	ABA	-28.9
**SI3792**	7a	8/10/15	Arnhem Highway, ERA Mining Lease	12º29'00.86"S	132º53'49.45"E	Open forest and woodland	1479.67	6.63538	Charred in a multi-temperature fire	ABA	-28.5
**SI3793**	8a	8/10/15	Arnhem Highway, ERA Mining Lease	12º29'00.76"S	132º54'00.99"E	Open forest and woodland	1479.67	6.63538	Charred in a multi-temperature fire	ABA	-28.4
**SI3794**	9a	8/10/15	Arnhem Highway, ERA Mining Lease	12º29'01.28"S	132º54'03.24"E	Open forest and woodland	1479.67	6.63538	Charred in a multi-temperature fire	ABA	-25.4
**SI3795**	10a	8/10/15	Arnhem Highway, ERA Mining Lease	12º28'59.94"S	132º53'55.09"E	Open forest and woodland	1479.67	6.63538	Charred in a multi-temperature fire	ABA	-30.0
**SI3796**	11a	8/10/15	Arnhem Highway, ERA Mining Lease	12º29'01.00"S	132º53'53.78"E	Open forest and woodland	1479.67	6.63538	Charred in a multi-temperature fire	ABA	-29.0
**SI3797**	12a	8/10/15	Arnhem Highway, ERA Mining Lease	12º28'59.37"S	132º53'50.47"E	Open forest and woodland	1479.67	6.63538	Charred in a multi-temperature fire	ABA	-29.8
**SI3799**	14a	8/10/15	Arnhem Highway, ERA Mining Lease	12º29'02.88"S	132º53'51.55"E	Open forest and woodland	1479.67	6.63538	Charred in a multi-temperature fire	ABA	-29.9

**SI3810**	27a	9/10/15	Arnhem Highway, ERA Mining Lease	12º33'29.05"S	132º53'55.13"E	Floodplain fringe	1523.42	6.67575	Charred in a multi-temperature fire	ABA	-28.6
**SI3811**	28a	9/10/15	Arnhem Highway, ERA Mining Lease	12º33'29.64"S	132º53'53.07"E	Floodplain fringe	1523.42	6.67575	Charred in a multi-temperature fire	ABA	-29.9
**SI3812**	29a	9/10/15	Arnhem Highway, ERA Mining Lease	12º33'24.55"S	132º53'54.49"E	Floodplain fringe	1523.42	6.67575	Charred in a multi-temperature fire	ABA	-29.2
**SI3813**	30a	9/10/15	Arnhem Highway, ERA Mining Lease	12º33'21.09"S	132º53'53.22"E	Floodplain fringe	1523.42	6.67575	Charred in a multi-temperature fire	ABA	-27.6
**SI3814**	31a	9/10/15	Arnhem Highway, ERA Mining Lease	12º33'19.25"S	132º54'00.27"E	Floodplain fringe	1523.42	6.67575	Charred in a multi-temperature fire	ABA	-27.8
**SI3815**	32a	9/10/15	Arnhem Highway, ERA Mining Lease	12º33'20.13"S	132º54'01.65"E	Floodplain fringe	1523.42	6.67575	Charred in a multi-temperature fire	ABA	-28.0
**SI3816**	33a	9/10/15	Arnhem Highway, ERA Mining Lease	12º33'19.37"S	132º54'03.21"E	Floodplain fringe	1523.42	6.67575	Charred in a multi-temperature fire	ABA	-28.7
**SI3817**	34a	9/10/15	Arnhem Highway, ERA Mining Lease	12º33'21.60"S	132º54'03.28"E	Floodplain fringe	1523.42	6.67575	Charred in a multi-temperature fire	ABA	-28.4
**SI3818**	35a	9/10/15	Arnhem Highway, ERA Mining Lease	12º33'22.37"S	132º54'03.20"E	Floodplain fringe	1523.42	6.67575	Charred in a multi-temperature fire	ABA	-28.0
**SI3819**	36a	9/10/15	Arnhem Highway, ERA Mining Lease	12º33'23.31"S	132º54'04.54"E	Floodplain fringe	1523.42	6.67575	Charred in a multi-temperature fire	ABA	-27.2
**SI3820**	42a	10/10/15	Arnhem Highway, ERA Mining Lease	12º32'27.63"S	132º54'11.77"E	Open forest and woodland	1517.72	6.67106	Charred in a multi-	ABA	-28.1

									temperature fire		
**SI3821**	43a	10/10/15	Arnhem Highway, ERA Mining Lease	12º32'27.20"S	132º54'12.76"E	Open forest and woodland	1517.72	6.67106	Charred in a multi-temperature fire	ABA	-28.4
**SI3823**	45a	10/10/15	Arnhem Highway, ERA Mining Lease	12º32'04.37"S	132º54'09.40"E	Seasonal floodway	1517.71	6.66599	Charred in a multi-temperature fire	ABA	-27.9
**SI3824**	46a	10/10/15	Arnhem Highway, ERA Mining Lease	12º32'04.65"S	132º54'11.30"E	Seasonal floodway	1517.71	6.66599	Charred in a multi-temperature fire	ABA	-29.4
**SI3825**	47a	10/10/15	Arnhem Highway, ERA Mining Lease	12º32'04.39"S	132º54'13.33"E	Seasonal floodway	1517.71	6.66599	Charred in a multi-temperature fire	ABA	-28.9
**SI3826**	48a	10/10/15	Arnhem Highway, ERA Mining Lease	12º32'04.52"S	132º54'15.54"E	Seasonal floodway	1517.71	6.66599	Charred in a multi-temperature fire	ABA	-29.1
**SI3827**	49a	10/10/15	Arnhem Highway, ERA Mining Lease	12º32'03.71"S	132º54'15.65"E	Seasonal floodway	1517.71	6.66599	Charred in a multi-temperature fire	ABA	-28.3
**SI3828**	50a	10/10/15	Arnhem Highway, ERA Mining Lease	12º32'05.66"S	132º54'19.51"E	Seasonal floodway	1517.71	6.66599	Charred in a multi-temperature fire	ABA	-27.7
**SI3829**	51a	10/10/15	Arnhem Highway, ERA Mining Lease	12º32'05.66"S	132º54'20.43"E	Seasonal floodway	1517.72	6.66599	Charred in a multi-temperature fire	ABA	-27.7
**SI3830**	5b	8/10/15	Arnhem Highway, ERA Mining Lease	12º28'51.23"S	132º54'02.57"E	Open forest and woodland	1479.67	6.63538	Charred at 500ºC for 2 hours	ABA	-28.8
**SI3831**	6b	8/10/15	Arnhem Highway, ERA Mining Lease	12º29'01.28"S	132º53'58.51"E	Open forest and woodland	1479.67	6.63538	Charred at 500ºC for 2 hours	ABA	-29.3

**SI3832**	7b	8/10/15	Arnhem Highway, ERA Mining Lease	12º29'00.86"S	132º53'49.45"E	Open forest and woodland	1479.67	6.63538	Charred at 500ºC for 2 hours	ABA	-28.3
**SI3833**	8b	8/10/15	Arnhem Highway, ERA Mining Lease	12º29'00.76"S	132º54'00.99"E	Open forest and woodland	1479.67	6.63538	Charred at 500ºC for 2 hours	ABA	-29.1
**SI3834**	9b	8/10/15	Arnhem Highway, ERA Mining Lease	12º29'01.28"S	132º54'03.24"E	Open forest and woodland	1479.67	6.63538	Charred at 500ºC for 2 hours	ABA	-26.1
**SI3835**	10b	8/10/15	Arnhem Highway, ERA Mining Lease	12º28'59.94"S	132º53'55.09"E	Open forest and woodland	1479.67	6.63538	Charred at 500ºC for 2 hours	ABA	-30.0
**SI3836**	11b	8/10/15	Arnhem Highway, ERA Mining Lease	12º29'01.00"S	132º53'53.78"E	Open forest and woodland	1479.67	6.63538	Charred at 500ºC for 2 hours	ABA	-28.7
**SI3837**	12b	8/10/15	Arnhem Highway, ERA Mining Lease	12º28'59.37"S	132º53'50.47"E	Open forest and woodland	1479.67	6.63538	Charred at 500ºC for 2 hours	ABA	-29.2
**SI3839**	14b	8/10/15	Arnhem Highway, ERA Mining Lease	12º29'02.88"S	132º53'51.55"E	Open forest and woodland	1479.67	6.63538	Charred at 500ºC for 2 hours	ABA	-30.1
**SI3850**	27b	9/10/15	Arnhem Highway, ERA Mining Lease	12º33'29.05"S	132º53'55.13"E	Floodplain fringe	1523.42	6.67575	Charred at 500ºC for 2 hours	ABA	-28.4
**SI3851**	28b	9/10/15	Arnhem Highway, ERA Mining Lease	12º33'29.64"S	132º53'53.07"E	Floodplain fringe	1523.42	6.67575	Charred at 500ºC for 2 hours	ABA	-29.4
**SI3852**	29b	9/10/15	Arnhem Highway, ERA Mining Lease	12º33'24.55"S	132º53'54.49"E	Floodplain fringe	1523.42	6.67575	Charred at 500ºC for 2 hours	ABA	-29.2
**SI3853**	30b	9/10/15	Arnhem Highway, ERA Mining Lease	12º33'21.09"S	132º53'53.22"E	Floodplain fringe	1523.42	6.67575	Charred at 500ºC for 2 hours	ABA	-27.4
**SI3854**	31b	9/10/15	Arnhem Highway, ERA Mining Lease	12º33'19.25"S	132º54'00.27"E	Floodplain fringe	1523.42	6.67575	Charred at 500ºC for 2 hours	ABA	-28.1
**SI3855**	32b	9/10/15	Arnhem Highway, ERA Mining Lease	12º33'20.13"S	132º54'01.65"E	Floodplain fringe	1523.42	6.67575	Charred at 500ºC for 2 hours	ABA	-28.2
**SI3856**	33b	9/10/15	Arnhem Highway, ERA Mining Lease	12º33'19.37"S	132º54'03.21"E	Floodplain fringe	1523.42	6.67575	Charred at 500ºC for 2 hours	ABA	-28.7

**SI3857**	34b	9/10/15	Arnhem Highway, ERA Mining Lease	12º33'21.60"S	132º54'03.28"E	Floodplain fringe	1523.42	6.67575	Charred at 500ºC for 2 hours	ABA	-28.3
**SI3858**	35b	9/10/15	Arnhem Highway, ERA Mining Lease	12º33'22.37"S	132º54'03.20"E	Floodplain fringe	1523.42	6.67575	Charred at 500ºC for 2 hours	ABA	-27.9
**SI3859**	36b	9/10/15	Arnhem Highway, ERA Mining Lease	12º33'23.31"S	132º54'04.54"E	Floodplain fringe	1523.42	6.67575	Charred at 500ºC for 2 hours	ABA	-27.4
**SI3860**	42b	10/10/15	Arnhem Highway, ERA Mining Lease	12º32'27.63"S	132º54'11.77"E	Open forest and woodland	1517.72	6.67106	Charred at 500ºC for 2 hours	ABA	-28.1
**SI3861**	43b	10/10/15	Arnhem Highway, ERA Mining Lease	12º32'27.20"S	132º54'12.76"E	Open forest and woodland	1517.72	6.67106	Charred at 500ºC for 2 hours	ABA	-28.2
**SI3863**	45b	10/10/15	Arnhem Highway, ERA Mining Lease	12º32'04.37"S	132º54'09.40"E	Seasonal floodway	1517.71	6.66599	Charred at 500ºC for 2 hours	ABA	-28.0
**SI3864**	46b	10/10/15	Arnhem Highway, ERA Mining Lease	12º32'04.65"S	132º54'11.30"E	Seasonal floodway	1517.71	6.66599	Charred at 500ºC for 2 hours	ABA	-29.6
**SI3865**	47b	10/10/15	Arnhem Highway, ERA Mining Lease	12º32'04.39"S	132º54'13.33"E	Seasonal floodway	1517.71	6.66599	Charred at 500ºC for 2 hours	ABA	-29.0
**SI3866**	48b	10/10/15	Arnhem Highway, ERA Mining Lease	12º32'04.52"S	132º54'15.54"E	Seasonal floodway	1517.71	6.66599	Charred at 500ºC for 2 hours	ABA	-29.3
**SI3867**	49b	10/10/15	Arnhem Highway, ERA Mining Lease	12º32'03.71"S	132º54'15.65"E	Seasonal floodway	1517.71	6.66599	Charred at 500ºC for 2 hours	ABA	-28.8
**SI3868**	50b	10/10/15	Arnhem Highway, ERA Mining Lease	12º32'05.66"S	132º54'19.51"E	Seasonal floodway	1517.71	6.66599	Charred at 500ºC for 2 hours	ABA	-28.2
**SI3869**	51b	10/10/15	Arnhem Highway, ERA Mining Lease	12º32'05.66"S	132º54'20.43"E	Seasonal floodway	1517.71	6.66599	Charred at 500ºC for 2 hours	ABA	-28.2
**SI3830**	5c	8/10/15	Arnhem Highway, ERA Mining Lease	12º28'51.23"S	132º54'02.57"E	Open forest and woodland	1479.67	6.63538	Charred at 400ºC for 4 hours	ABA	-28.8
**SI3831**	6c	8/10/15	Arnhem Highway, ERA Mining Lease	12º29'01.28"S	132º53'58.51"E	Open forest and woodland	1479.67	6.63538	Charred at 400ºC for 4 hours	ABA	-29.1

**SI3832**	7c	8/10/15	Arnhem Highway, ERA Mining Lease	12º29'00.86"S	132º53'49.45"E	Open forest and woodland	1479.67	6.63538	Charred at 400ºC for 4 hours	ABA	-28.3
**SI3833**	8c	8/10/15	Arnhem Highway, ERA Mining Lease	12º29'00.76"S	132º54'00.99"E	Open forest and woodland	1479.67	6.63538	Charred at 400ºC for 4 hours	ABA	-29.0
**SI3834**	9c	8/10/15	Arnhem Highway, ERA Mining Lease	12º29'01.28"S	132º54'03.24"E	Open forest and woodland	1479.67	6.63538	Charred at 400ºC for 4 hours	ABA	-25.9
**SI3835**	10c	8/10/15	Arnhem Highway, ERA Mining Lease	12º28'59.94"S	132º53'55.09"E	Open forest and woodland	1479.67	6.63538	Charred at 400ºC for 4 hours	ABA	-29.6
**SI3836**	11c	8/10/15	Arnhem Highway, ERA Mining Lease	12º29'01.00"S	132º53'53.78"E	Open forest and woodland	1479.67	6.63538	Charred at 400ºC for 4 hours	ABA	-28.9
**SI3837**	12c	8/10/15	Arnhem Highway, ERA Mining Lease	12º28'59.37"S	132º53'50.47"E	Open forest and woodland	1479.67	6.63538	Charred at 400ºC for 4 hours	ABA	-29.2
**SI3839**	14c	8/10/15	Arnhem Highway, ERA Mining Lease	12º29'02.88"S	132º53'51.55"E	Open forest and woodland	1479.67	6.63538	Charred at 400ºC for 4 hours	ABA	-29.8
**SI3850**	27c	9/10/15	Arnhem Highway, ERA Mining Lease	12º33'29.05"S	132º53'55.13"E	Floodplain fringe	1523.42	6.67575	Charred at 400ºC for 4 hours	ABA	-28.6
**SI3851**	28c	9/10/15	Arnhem Highway, ERA Mining Lease	12º33'29.64"S	132º53'53.07"E	Floodplain fringe	1523.42	6.67575	Charred at 400ºC for 4 hours	ABA	-29.2
**SI3852**	29c	9/10/15	Arnhem Highway, ERA Mining Lease	12º33'24.55"S	132º53'54.49"E	Floodplain fringe	1523.42	6.67575	Charred at 400ºC for 4 hours	ABA	-29.0
**SI3853**	30c	9/10/15	Arnhem Highway, ERA Mining Lease	12º33'21.09"S	132º53'53.22"E	Floodplain fringe	1523.42	6.67575	Charred at 400ºC for 4 hours	ABA	-27.3
**SI3854**	31c	9/10/15	Arnhem Highway, ERA Mining Lease	12º33'19.25"S	132º54'00.27"E	Floodplain fringe	1523.42	6.67575	Charred at 400ºC for 4 hours	ABA	-27.7
**SI3855**	32c	9/10/15	Arnhem Highway, ERA Mining Lease	12º33'20.13"S	132º54'01.65"E	Floodplain fringe	1523.42	6.67575	Charred at 400ºC for 4 hours	ABA	-28.4
**SI3856**	33c	9/10/15	Arnhem Highway, ERA Mining Lease	12º33'19.37"S	132º54'03.21"E	Floodplain fringe	1523.42	6.67575	Charred at 400ºC for 4 hours	ABA	-28.7

**SI3857**	34c	9/10/15	Arnhem Highway, ERA Mining Lease	12º33'21.60"S	132º54'03.28"E	Floodplain fringe	1523.42	6.67575	Charred at 400ºC for 4 hours	ABA	-28.1
**SI3858**	35c	9/10/15	Arnhem Highway, ERA Mining Lease	12º33'22.37"S	132º54'03.20"E	Floodplain fringe	1523.42	6.67575	Charred at 400ºC for 4 hours	ABA	-28.0
**SI3859**	36c	9/10/15	Arnhem Highway, ERA Mining Lease	12º33'23.31"S	132º54'04.54"E	Floodplain fringe	1523.42	6.67575	Charred at 400ºC for 4 hours	ABA	-27.3
**SI3860**	42c	10/10/15	Arnhem Highway, ERA Mining Lease	12º32'27.63"S	132º54'11.77"E	Open forest and woodland	1517.72	6.67106	Charred at 400ºC for 4 hours	ABA	-28.1
**SI3861**	43c	10/10/15	Arnhem Highway, ERA Mining Lease	12º32'27.20"S	132º54'12.76"E	Open forest and woodland	1517.72	6.67106	Charred at 400ºC for 4 hours	ABA	-28.2
**SI3863**	45c	10/10/15	Arnhem Highway, ERA Mining Lease	12º32'04.37"S	132º54'09.40"E	Seasonal floodway	1517.71	6.66599	Charred at 400ºC for 4 hours	ABA	-27.9
**SI3864**	46c	10/10/15	Arnhem Highway, ERA Mining Lease	12º32'04.65"S	132º54'11.30"E	Seasonal floodway	1517.71	6.66599	Charred at 400ºC for 4 hours	ABA	-28.9
**SI3865**	47c	10/10/15	Arnhem Highway, ERA Mining Lease	12º32'04.39"S	132º54'13.33"E	Seasonal floodway	1517.71	6.66599	Charred at 400ºC for 4 hours	ABA	-28.9
**SI3866**	48c	10/10/15	Arnhem Highway, ERA Mining Lease	12º32'04.52"S	132º54'15.54"E	Seasonal floodway	1517.71	6.66599	Charred at 400ºC for 4 hours	ABA	-29.1
**SI3867**	49c	10/10/15	Arnhem Highway, ERA Mining Lease	12º32'03.71"S	132º54'15.65"E	Seasonal floodway	1517.71	6.66599	Charred at 400ºC for 4 hours	ABA	-28.6
**SI3868**	50c	10/10/15	Arnhem Highway, ERA Mining Lease	12º32'05.66"S	132º54'19.51"E	Seasonal floodway	1517.71	6.66599	Charred at 400ºC for 4 hours	ABA	-28.1
**SI3869**	51c	10/10/15	Arnhem Highway, ERA Mining Lease	12º32'05.66"S	132º54'20.43"E	Seasonal floodway	1517.71	6.66599	Charred at 400ºC for 4 hours	ABA	-28.1
**SI3830**	5d	8/10/15	Arnhem Highway, ERA Mining Lease	12º28'51.23"S	132º54'02.57"E	Open forest and woodland	1479.67	6.63538	Dried	Alpha-cellulose	-28.3
**SI3831**	6d	8/10/15	Arnhem Highway, ERA Mining Lease	12º29'01.28"S	132º53'58.51"E	Open forest and woodland	1479.67	6.63538	Dried	Alpha-cellulose	-28.7

**SI3832**	7d	8/10/15	Arnhem Highway, ERA Mining Lease	12º29'00.86"S	132º53'49.45"E	Open forest and woodland	1479.67	6.63538	Dried	Alpha-cellulose	-28.1
**SI3833**	8d	8/10/15	Arnhem Highway, ERA Mining Lease	12º29'00.76"S	132º54'00.99"E	Open forest and woodland	1479.67	6.63538	Dried	Alpha-cellulose	-28.8
**SI3834**	9d	8/10/15	Arnhem Highway, ERA Mining Lease	12º29'01.28"S	132º54'03.24"E	Open forest and woodland	1479.67	6.63538	Dried	Alpha-cellulose	-25.5
**SI3835**	10d	8/10/15	Arnhem Highway, ERA Mining Lease	12º28'59.94"S	132º53'55.09"E	Open forest and woodland	1479.67	6.63538	Dried	Alpha-cellulose	-28.9
**SI3836**	11d	8/10/15	Arnhem Highway, ERA Mining Lease	12º29'01.00"S	132º53'53.78"E	Open forest and woodland	1479.67	6.63538	Dried	Alpha-cellulose	-28.3
**SI3837**	12d	8/10/15	Arnhem Highway, ERA Mining Lease	12º28'59.37"S	132º53'50.47"E	Open forest and woodland	1479.67	6.63538	Dried	Alpha-cellulose	-28.6
**SI3839**	14d	8/10/15	Arnhem Highway, ERA Mining Lease	12º29'02.88"S	132º53'51.55"E	Open forest and woodland	1479.67	6.63538	Dried	Alpha-cellulose	-29.5
**SI3850**	27d	9/10/15	Arnhem Highway, ERA Mining Lease	12º33'29.05"S	132º53'55.13"E	Floodplain fringe	1523.42	6.67575	Dried	Alpha-cellulose	-28.0
**SI3851**	28d	9/10/15	Arnhem Highway, ERA Mining Lease	12º33'29.64"S	132º53'53.07"E	Floodplain fringe	1523.42	6.67575	Dried	Alpha-cellulose	-28.9
**SI3852**	29d	9/10/15	Arnhem Highway, ERA Mining Lease	12º33'24.55"S	132º53'54.49"E	Floodplain fringe	1523.42	6.67575	Dried	Alpha-cellulose	-28.1
**SI3853**	30d	9/10/15	Arnhem Highway, ERA Mining Lease	12º33'21.09"S	132º53'53.22"E	Floodplain fringe	1523.42	6.67575	Dried	Alpha-cellulose	-26.4
**SI3854**	31d	9/10/15	Arnhem Highway, ERA Mining Lease	12º33'19.25"S	132º54'00.27"E	Floodplain fringe	1523.42	6.67575	Dried	Alpha-cellulose	-27.4
**SI3855**	32d	9/10/15	Arnhem Highway, ERA Mining Lease	12º33'20.13"S	132º54'01.65"E	Floodplain fringe	1523.42	6.67575	Dried	Alpha-cellulose	-27.4
**SI3856**	33d	9/10/15	Arnhem Highway, ERA Mining Lease	12º33'19.37"S	132º54'03.21"E	Floodplain fringe	1523.42	6.67575	Dried	Alpha-cellulose	-27.8

**SI3857**	34d	9/10/15	Arnhem Highway, ERA Mining Lease	12º33'21.60"S	132º54'03.28"E	Floodplain fringe	1523.42	6.67575	Dried	Alpha-cellulose	-28.1
**SI3858**	35d	9/10/15	Arnhem Highway, ERA Mining Lease	12º33'22.37"S	132º54'03.20"E	Floodplain fringe	1523.42	6.67575	Dried	Alpha-cellulose	-27.4
**SI3859**	36d	9/10/15	Arnhem Highway, ERA Mining Lease	12º33'23.31"S	132º54'04.54"E	Floodplain fringe	1523.42	6.67575	Dried	Alpha-cellulose	-26.8
**SI3860**	42d	10/10/15	Arnhem Highway, ERA Mining Lease	12º32'27.63"S	132º54'11.77"E	Open forest and woodland	1517.72	6.67106	Dried	Alpha-cellulose	-27.7
**SI3861**	43d	10/10/15	Arnhem Highway, ERA Mining Lease	12º32'27.20"S	132º54'12.76"E	Open forest and woodland	1517.72	6.67106	Dried	Alpha-cellulose	-27.8
**SI3863**	45d	10/10/15	Arnhem Highway, ERA Mining Lease	12º32'04.37"S	132º54'09.40"E	Seasonal floodway	1517.71	6.66599	Dried	Alpha-cellulose	-27.3
**SI3864**	46d	10/10/15	Arnhem Highway, ERA Mining Lease	12º32'04.65"S	132º54'11.30"E	Seasonal floodway	1517.71	6.66599	Dried	Alpha-cellulose	-29.0
**SI3865**	47d	10/10/15	Arnhem Highway, ERA Mining Lease	12º32'04.39"S	132º54'13.33"E	Seasonal floodway	1517.71	6.66599	Dried	Alpha-cellulose	-28.5
**SI3866**	48d	10/10/15	Arnhem Highway, ERA Mining Lease	12º32'04.52"S	132º54'15.54"E	Seasonal floodway	1517.71	6.66599	Dried	Alpha-cellulose	-28.7
**SI3867**	49d	10/10/15	Arnhem Highway, ERA Mining Lease	12º32'03.71"S	132º54'15.65"E	Seasonal floodway	1517.71	6.66599	Dried	Alpha-cellulose	-27.9
**SI3868**	50d	10/10/15	Arnhem Highway, ERA Mining Lease	12º32'05.66"S	132º54'19.51"E	Seasonal floodway	1517.71	6.66599	Dried	Alpha-cellulose	-27.4
**SI3869**	51d	10/10/15	Arnhem Highway, ERA Mining Lease	12º32'05.66"S	132º54'20.43"E	Seasonal floodway	1517.71	6.66599	Dried	Alpha-cellulose	-27.7
**SI4396**	72	18/10/16	Arnhem Highway, ERA Mining Lease	12º28'51.20"S	132º54'02.60"E	Open forest and woodland	1479.67	6.63538	Charred at 500ºC for 2 hours	ABA	-29.5
**SI4397**	73	18/10/16	Arnhem Highway, ERA Mining Lease	12º29'01.16"S	132º54'03.26"E	Open forest and woodland	1479.67	6.63538	Charred at 500ºC for 2 hours	ABA	-27.7

**SI4398**	74	18/10/16	Arnhem Highway, ERA Mining Lease	12º29'00.77"S	132º54'00.99"E	Open forest and woodland	1479.67	6.63538	Charred at 500ºC for 2 hours	ABA	-29.1
**SI4399**	75	18/10/16	Arnhem Highway, ERA Mining Lease	12º29'00.90"S	132º53'59.22"E	Open forest and woodland	1479.67	6.63538	Charred at 500ºC for 2 hours	ABA	-28.8
**SI4400**	76	18/10/16	Arnhem Highway, ERA Mining Lease	12º29'01.29"S	132º53'58.58"E	Open forest and woodland	1479.67	6.63538	Charred at 500ºC for 2 hours	ABA	-29.5
**SI4401**	77	18/10/16	Arnhem Highway, ERA Mining Lease	12º28'60.00"S	132º53'54.87"E	Open forest and woodland	1479.67	6.63538	Charred at 500ºC for 2 hours	ABA	-30.2
**SI4402**	78	18/10/16	Arnhem Highway, ERA Mining Lease	12º29'00.97"S	132º53'53.65"E	Open forest and woodland	1479.67	6.63538	Charred at 500ºC for 2 hours	ABA	-28.5
**SI4403**	79	18/10/16	Arnhem Highway, ERA Mining Lease	12º28'59.12"S	132º53'50.41"E	Open forest and woodland	1479.67	6.63538	Charred at 500ºC for 2 hours	ABA	-29.0
**SI4404**	80	18/10/16	Arnhem Highway, ERA Mining Lease	12º29'04.24"S	132º53'48.91"E	Open forest and woodland	1479.67	6.63538	Charred at 500ºC for 2 hours	ABA	-29.2
**SI4405**	81	18/10/16	Arnhem Highway, ERA Mining Lease	12º29'02.90"S	132º53'51.55"E	Open forest and woodland	1479.67	6.63538	Charred at 500ºC for 2 hours	ABA	-30.0
**SI4406**	82	18/10/16	Arnhem Highway, ERA Mining Lease	12º33'24.28"S	132º53'54.51"E	Floodplain fringe	1523.42	6.67575	Charred at 500ºC for 2 hours	ABA	-28.7
**SI4407**	83	18/10/16	Arnhem Highway, ERA Mining Lease	12º33'20.91"S	132º53'53.10"E	Floodplain fringe	1523.42	6.67575	Charred at 500ºC for 2 hours	ABA	-27.5
**SI4408**	84	18/10/16	Arnhem Highway, ERA Mining Lease	12º33'19.10"S	132º54'00.34"E	Floodplain fringe	1523.42	6.67575	Charred at 500ºC for 2 hours	ABA	-28.3
**SI4409**	85	18/10/16	Arnhem Highway, ERA Mining Lease	12º33'18.04"S	132º54'00.17"E	Floodplain fringe	1523.42	6.67575	Charred at 500ºC for 2 hours	ABA	-28.7
**SI4410**	86	18/10/16	Arnhem Highway, ERA Mining Lease	12º33'19.99"S	132º54'01.55"E	Floodplain fringe	1523.42	6.67575	Charred at 500ºC for 2 hours	ABA	-28.8
**SI4411**	87	18/10/16	Arnhem Highway, ERA Mining Lease	12º33'19.20"S	132º54'03.11"E	Floodplain fringe	1523.42	6.67575	Charred at 500ºC for 2 hours	ABA	-28.6

**SI4412**	88	18/10/16	Arnhem Highway, ERA Mining Lease	12º33'21.30"S	132º54'03.11"E	Floodplain fringe	1523.42	6.67575	Charred at 500ºC for 2 hours	ABA	-29.0
**SI4413**	89	18/10/16	Arnhem Highway, ERA Mining Lease	12º33'22.49"S	132º54'03.62"E	Floodplain fringe	1523.42	6.67575	Charred at 500ºC for 2 hours	ABA	-30.0
**SI4414**	90	18/10/16	Arnhem Highway, ERA Mining Lease	12º33'23.10"S	132º54'04.72"E	Floodplain fringe	1523.42	6.67575	Charred at 500ºC for 2 hours	ABA	-28.4
**SI4415**	91	18/10/16	Arnhem Highway, ERA Mining Lease	12º33'19.02"S	132º54'07.24"E	Floodplain fringe	1523.42	6.67575	Charred at 500ºC for 2 hours	ABA	-29.3
**SI4416**	92	19/10/16	Arnhem Highway, ERA Mining Lease	12º32'27.53"S	132º54'11.70"E	Open forest and woodland	1517.72	6.67106	Charred at 500ºC for 2 hours	ABA	-29.2
**SI4417**	93	19/10/16	Arnhem Highway, ERA Mining Lease	12º32'27.15"S	132º54'12.46"E	Open forest and woodland	1517.72	6.67106	Charred at 500ºC for 2 hours	ABA	-28.5
**SI4418**	94	19/10/16	Arnhem Highway, ERA Mining Lease	12º32'25.30"S	132º54'09.64"E	Seasonal causeway	1517.72	6.67106	Charred at 500ºC for 2 hours	ABA	-28.1
**SI4419**	95	19/10/16	Arnhem Highway, ERA Mining Lease	12º32'04.35"S	132º54'09.37"E	Seasonal causeway	1511.71	6.66599	Charred at 500ºC for 2 hours	ABA	-28.3
**SI4420**	96	19/10/16	Arnhem Highway, ERA Mining Lease	12º32'04.54"S	132º54'11.11"E	Seasonal causeway	1511.71	6.66599	Charred at 500ºC for 2 hours	ABA	-28.9
**SI4421**	97	19/10/16	Arnhem Highway, ERA Mining Lease	12º32'04.41"S	132º54'13.21"E	Seasonal causeway	1511.71	6.66599	Charred at 500ºC for 2 hours	ABA	-29.3
**SI4422**	98	19/10/16	Arnhem Highway, ERA Mining Lease	12º32'04.45"S	132º54'15.47"E	Seasonal causeway	1511.71	6.66599	Charred at 500ºC for 2 hours	ABA	-29.2
**SI4423**	99	19/10/16	Arnhem Highway, ERA Mining Lease	12º32'03.51"S	132º54'15.32"E	Seasonal causeway	1511.71	6.66599	Charred at 500ºC for 2 hours	ABA	-28.9
**SI4424**	100	19/10/16	Arnhem Highway, ERA Mining Lease	12º32'05.51"S	132º54'19.41"E	Seasonal causeway	1511.71	6.66599	Charred at 500ºC for 2 hours	ABA	-28.8
**SI4425**	101	19/10/16	Arnhem Highway, ERA Mining Lease	12º32'05.51"S	132º54'19.41"E	Seasonal causeway	1511.71	6.66599	Charred at 500ºC for 2 hours	ABA	-28.1

**SI5263**	163	26/9/17	Arnhem Highway, ERA Mining Lease	12º33'23.01"S	132º54'04.71"E	Floodplain fringe	1523.42	6.67575	Charred at 500ºC for 2 hours	ABA	-28.4
**SI5264**	164	26/9/17	Arnhem Highway, ERA Mining Lease	12º33'22.42"S	132º54'03.48"E	Floodplain fringe	1523.42	6.67575	Charred at 500ºC for 2 hours	ABA	-29.0
**SI5265**	165	26/9/17	Arnhem Highway, ERA Mining Lease	12º33'21.20"S	132º54'02.88"E	Floodplain fringe	1523.42	6.67575	Charred at 500ºC for 2 hours	ABA	-28.2
**SI5266**	166	26/9/17	Arnhem Highway, ERA Mining Lease	12º33'19.14"S	132º54'03.17"E	Floodplain fringe	1523.42	6.67575	Charred at 500ºC for 2 hours	ABA	-27.6
**SI5267**	167	26/9/17	Arnhem Highway, ERA Mining Lease	12º33'20.97"S	132º54'01.48"E	Floodplain fringe	1523.42	6.67575	Charred at 500ºC for 2 hours	ABA	-28.6
**SI5268**	168	26/9/17	Arnhem Highway, ERA Mining Lease	12º33'19.03"S	132º54'00.29"E	Floodplain fringe	1523.42	6.67575	Charred at 500ºC for 2 hours	ABA	-28.9
**SI5269**	169	26/9/17	Arnhem Highway, ERA Mining Lease	12º33'20.78"S	132º53'53.08"E	Floodplain fringe	1523.42	6.67575	Charred at 500ºC for 2 hours	ABA	-27.6
**SI5270**	170	26/9/17	Arnhem Highway, ERA Mining Lease	12º33'24.33"S	132º53'54.48"E	Floodplain fringe	1523.42	6.67575	Charred at 500ºC for 2 hours	ABA	-29.2
**SI5271**	171	26/9/17	Arnhem Highway, ERA Mining Lease	12º33'18.94"S	132º54'07.04"E	Floodplain fringe	1523.42	6.67575	Charred at 500ºC for 2 hours	ABA	-28.8
**SI5272**	172	26/9/17	Arnhem Highway, ERA Mining Lease	12º32'27.57"S	132º54'11.64"E	Open forest and woodland	1517.72	6.67106	Charred at 500ºC for 2 hours	ABA	-28.4
**SI5273**	173	26/9/17	Arnhem Highway, ERA Mining Lease	12º32'27.05"S	132º54'12.56"E	Open forest and woodland	1517.72	6.67106	Charred at 500ºC for 2 hours	ABA	-28.7
**SI5274**	174	26/9/17	Arnhem Highway, ERA Mining Lease	12º32'25.34"S	132º54'09.66"E	Open forest and woodland	1517.72	6.67106	Charred at 500ºC for 2 hours	ABA	-28.7
**SI5275**	175	26/9/17	Arnhem Highway, ERA Mining Lease	12º32'04.29"S	132º54'09.41"E	Seasonal causeway	1511.71	6.66599	Charred at 500ºC for 2 hours	ABA	-28.7
**SI5276**	176	26/9/17	Arnhem Highway, ERA Mining Lease	12º32'04.54"S	132º54'11.13"E	Seasonal causeway	1511.71	6.66599	Charred at 500ºC for 2 hours	ABA	-29.6

**SI5277**	177	26/9/17	Arnhem Highway, ERA Mining Lease	12º33'04.35"S	132º54'13.31"E	Seasonal causeway	1511.71	6.66599	Charred at 500ºC for 2 hours	ABA	-30.0
**SI5278**	178	26/9/17	Arnhem Highway, ERA Mining Lease	12º32'04.43"S	132º54'15.52"E	Seasonal causeway	1511.71	6.66599	Charred at 500ºC for 2 hours	ABA	-29.2
**SI5279**	179	26/9/17	Arnhem Highway, ERA Mining Lease	12º32'03.48"S	132º54'15.39"E	Seasonal causeway	1511.71	6.66599	Charred at 500ºC for 2 hours	ABA	-30.0
**SI5280**	180	26/9/17	Arnhem Highway, ERA Mining Lease	12º32'05.60"S	132º54'19.51"E	Seasonal causeway	1511.71	6.66599	Charred at 500ºC for 2 hours	ABA	-28.5
**SI5282**	182	26/9/17	Arnhem Highway, ERA Mining Lease	12º29'01.27"S	132º54'03.16"E	Open forest and woodland	1479.67	6.63538	Charred at 500ºC for 2 hours	ABA	-27.9
**SI5283**	183	26/9/17	Arnhem Highway, ERA Mining Lease	12º29'00.72"S	132º54'01.98"E	Open forest and woodland	1479.67	6.63538	Charred at 500ºC for 2 hours	ABA	-30.2
**SI5284**	184	26/9/17	Arnhem Highway, ERA Mining Lease	12º29'00.86"S	132º53'59.37"E	Open forest and woodland	1479.67	6.63538	Charred at 500ºC for 2 hours	ABA	-27.9
**SI5285**	185	26/9/17	Arnhem Highway, ERA Mining Lease	12º28'59.94"S	132º53'55.96"E	Open forest and woodland	1479.67	6.63538	Charred at 500ºC for 2 hours	ABA	-27.8
**SI5286**	186	26/9/17	Arnhem Highway, ERA Mining Lease	12º29'02.78"S	132º53'51.61"E	Open forest and woodland	1479.67	6.63538	Charred at 500ºC for 2 hours	ABA	-29.5
**SI5287**	187	29/9/17	Arnhem Highway, ERA Mining Lease	12º32'37.92"S	132º54'16.52"E	Open forest and woodland	1517.72	6.67106	Charred at 500ºC for 2 hours	ABA	-29.1
**SI5288**	188	29/9/17	Arnhem Highway, ERA Mining Lease	12º32'37.52"S	132º54'17.14"E	Open forest and woodland	1517.72	6.67106	Charred at 500ºC for 2 hours	ABA	-29.1
**SI5289**	189	29/9/17	Arnhem Highway, ERA Mining Lease	12º32'37.67"S	132º54'18.24"E	Open forest and woodland	1517.72	6.67106	Charred at 500ºC for 2 hours	ABA	-30.5
**SI5290**	190	29/9/17	Arnhem Highway, ERA Mining Lease	12º32'40.76"S	132º54'20.28"E	Open forest and woodland	1517.72	6.67106	Charred at 500ºC for 2 hours	ABA	-27.9
**SI5291**	191	29/9/17	Arnhem Highway, ERA Mining Lease	12º32'41.92"S	132º54'22.80"E	Open forest and woodland	1517.72	6.67106	Charred at 500ºC for 2 hours	ABA	-29.7

**SI5292**	192	29/9/17	Arnhem Highway, ERA Mining Lease	12º31'39.99"S	132º53'57.26"E	Open forest and woodland	1505.45	6.66055	Charred at 500ºC for 2 hours	ABA	-30.1
**SI5293**	193	29/9/17	Arnhem Highway, ERA Mining Lease	12º31'39.97"S	132º53'59.89"E	Open forest and woodland	1505.45	6.66055	Charred at 500ºC for 2 hours	ABA	-30.4
**SI5294**	194	29/9/17	Arnhem Highway, ERA Mining Lease	12º31'38.14"S	132º54'03.33"E	Open forest and woodland	1505.45	6.66055	Charred at 500ºC for 2 hours	ABA	-28.6
**SI5295**	195	29/9/17	Arnhem Highway, ERA Mining Lease	12º31'36.03"S	132º54'04.66"E	Open forest and woodland	1505.45	6.66055	Charred at 500ºC for 2 hours	ABA	-29.7
**SI5296**	196	29/9/17	Arnhem Highway, ERA Mining Lease	12º31'35.16"S	132º54'08.79"E	Seasonal causeway	1505.45	6.66055	Charred at 500ºC for 2 hours	ABA	-29.5
**SI5616**	229a	11/7/18	Stuart Highway, Darwin	12º25'29.46"S	130º52'58.42"E	Open forest and woodland	1719.7	6.79898	Charred at 500ºC for 2 hours	ABA	-28.5
**SI5617**	229b	11/7/18	Stuart Highway, Darwin	12º25'29.46"S	130º52'58.42"E	Open forest and woodland	1719.7	6.79898	Charred at 500ºC for 2 hours	ABA	-28.2
**SI5618**	229c	11/7/18	Stuart Highway, Darwin	12º25'29.46"S	130º52'58.42"E	Open forest and woodland	1719.7	6.79898	Charred at 500ºC for 2 hours	ABA	-28.2
**SI5619**	230a	11/7/18	Stuart Highway, Darwin	12º25'29.51"S	130º53'06.23"E	Open forest and woodland	1719.7	6.79898	Charred at 500ºC for 2 hours	ABA	-27.3
**SI5620**	230b	11/7/18	Stuart Highway, Darwin	12º25'29.51"S	130º53'06.23"E	Open forest and woodland	1719.7	6.79898	Charred at 500ºC for 2 hours	ABA	-27.2
**SI5621**	230c	11/7/18	Stuart Highway, Darwin	12º25'29.51"S	130º53'06.23"E	Open forest and woodland	1719.7	6.79898	Charred at 500ºC for 2 hours	ABA	-26.9
**SI5622**	231a	11/7/18	Stuart Highway, Palmerston	12º29'01.39"S	131º00'30.40"E	Seasonal causeway	1652.36	6.75764	Charred at 500ºC for 2 hours	ABA	-26.8
**SI5623**	231b	11/7/18	Stuart Highway, Palmerston	12º29'01.39"S	131º00'30.40"E	Seasonal causeway	1652.36	6.75764	Charred at 500ºC for 2 hours	ABA	-26.8
**SI5624**	231c	11/7/18	Stuart Highway, Palmerston	12º29'01.39"S	131º00'30.40"E	Seasonal causeway	1652.36	6.75764	Charred at 500ºC for 2 hours	ABA	-26.8

**SI5625**	232a	11/7/18	Stuart Highway, Palmerston	12º29'00.32"S	131º00'29.63"E	Seasonal causeway	1652.36	6.75764	Charred at 500ºC for 2 hours	ABA	-28.1
**SI5626**	232b	11/7/18	Stuart Highway, Palmerston	12º29'00.32"S	131º00'29.63"E	Seasonal causeway	1652.36	6.75764	Charred at 500ºC for 2 hours	ABA	-27.7
**SI5627**	232c	11/7/18	Stuart Highway, Palmerston	12º29'00.32"S	131º00'29.63"E	Seasonal causeway	1652.36	6.75764	Charred at 500ºC for 2 hours	ABA	-28.0
**SI5628**	233a	11/7/18	Stuart Highway, Palmerston	12º28'59.89"S	131º00'28.91"E	Seasonal causeway	1652.36	6.75764	Charred at 500ºC for 2 hours	ABA	-27.9
**SI5629**	233b	11/7/18	Stuart Highway, Palmerston	12º28'59.89"S	131º00'28.91"E	Seasonal causeway	1652.36	6.75764	Charred at 500ºC for 2 hours	ABA	-28.4
**SI5630**	233c	11/7/18	Stuart Highway, Palmerston	12º28'59.89"S	131º00'28.91"E	Seasonal causeway	1652.36	6.75764	Charred at 500ºC for 2 hours	ABA	-28.1
**SI5631**	235a	11/7/18	Stuart Highway, Hughes	12º42'24.29"S	131º05'11.30"E	Open forest and woodland	1545	6.5737	Charred at 500ºC for 2 hours	ABA	-27.8
**SI5632**	235b	11/7/18	Stuart Highway, Hughes	12º42'24.29"S	131º05'11.30"E	Open forest and woodland	1545	6.5737	Charred at 500ºC for 2 hours	ABA	-27.9
**SI5633**	235c	11/7/18	Stuart Highway, Hughes	12º42'24.28"S	131º05'11.01"E	Open forest and woodland	1545	6.5737	Charred at 500ºC for 2 hours	ABA	-27.7
**SI5634**	236a	11/7/18	Stuart Highway, Hughes	12º43'46.47"S	131º05'19.52"E	Open forest and woodland	1540.55	6.53838	Charred at 500ºC for 2 hours	ABA	-28.5
**SI5635**	236b	11/7/18	Stuart Highway, Hughes	12º43'46.47"S	131º05'19.52"E	Open forest and woodland	1540.55	6.53838	Charred at 500ºC for 2 hours	ABA	-28.7
**SI5636**	236c	11/7/18	Stuart Highway, Hughes	12º43'46.47"S	131º05'19.52"E	Open forest and woodland	1540.55	6.53838	Charred at 500ºC for 2 hours	ABA	-28.6
**SI5637**	237a	11/7/18	Stuart Highway, Hughes	12º43'46.10"S	131º05'19.65"E	Open forest and woodland	1540.55	6.53838	Charred at 500ºC for 2 hours	ABA	-29.0
**SI5638**	237b	11/7/18	Stuart Highway, Hughes	12º43'46.10"S	131º05'19.65"E	Open forest and woodland	1540.55	6.53838	Charred at 500ºC for 2 hours	ABA	-28.2

**SI5639**	237c	11/7/18	Stuart Highway, Hughes	12º43'46.10"S	131º05'19.65"E	Open forest and woodland	1540.55	6.53838	Charred at 500ºC for 2 hours	ABA	-28.6
**SI5640**	238a	11/7/18	Stuart Highway, Lake Bennett	12º56'32.63"S	131º07'30.26"E	Seasonal causeway	1534.83	6.30063	Charred at 500ºC for 2 hours	ABA	-30.7
**SI5641**	238b	11/7/18	Stuart Highway, Lake Bennett	12º56'32.63"S	131º07'30.26"E	Seasonal causeway	1534.83	6.30063	Charred at 500ºC for 2 hours	ABA	-30.1
**SI5642**	238c	11/7/18	Stuart Highway, Lake Bennett	12º56'32.63"S	131º07'30.26"E	Seasonal causeway	1534.83	6.30063	Charred at 500ºC for 2 hours	ABA	-30.6
**SI5643**	239a	11/7/18	Stuart Highway, Lake Bennett	12º56'46.50"S	131º07'24.67"E	Seasonal causeway	1534.83	6.30063	Charred at 500ºC for 2 hours	ABA	-28.1
**SI5644**	239b	11/7/18	Stuart Highway, Lake Bennett	12º56'46.50"S	131º07'24.67"E	Seasonal causeway	1534.83	6.30063	Charred at 500ºC for 2 hours	ABA	-28.1
**SI5645**	239c	11/7/18	Stuart Highway, Lake Bennett	12º56'46.50"S	131º07'24.67"E	Seasonal causeway	1534.83	6.30063	Charred at 500ºC for 2 hours	ABA	-28.3
**SI5646**	240a	11/7/18	Stuart Highway, Stapleton	13º11'03.61"S	131º06'03.29"E	Seasonal causeway	1523.55	6.09692	Charred at 500ºC for 2 hours	ABA	-29.1
**SI5647**	240b	11/7/18	Stuart Highway, Stapleton	13º11'03.61"S	131º06'03.29"E	Seasonal causeway	1523.55	6.09692	Charred at 500ºC for 2 hours	ABA	-29.3
**SI5648**	240c	11/7/18	Stuart Highway, Stapleton	13º11'03.61"S	131º06'03.29"E	Seasonal causeway	1523.55	6.09692	Charred at 500ºC for 2 hours	ABA	-29.1
**SI5649**	243a	11/7/18	Stuart Highway, Adelaide River	13º13'34.70"S	131º06'01.68"E	Seasonal causeway	1514.27	6.07213	Charred at 500ºC for 2 hours	ABA	-28.4
**SI5650**	243b	11/7/18	Stuart Highway, Adelaide River	13º13'34.70"S	131º06'01.68"E	Seasonal causeway	1514.27	6.07213	Charred at 500ºC for 2 hours	ABA	-28.3
**SI5651**	243c	11/7/18	Stuart Highway, Adelaide River	13º13'34.70"S	131º06'01.68"E	Seasonal causeway	1514.27	6.07213	Charred at 500ºC for 2 hours	ABA	-28.1
**SI5652**	244a	11/7/18	Stuart Highway, Adelaide River	13º13'43.20"S	131º06'03.08"E	Seasonal causeway	1511.9	6.06639	Charred at 500ºC for 2 hours	ABA	-29.1

**SI5653**	244b	11/7/18	Stuart Highway, Adelaide River	13º13'43.20"S	131º06'03.08"E	Seasonal causeway	1511.9	6.06639	Charred at 500ºC for 2 hours	ABA	-29.3
**SI5654**	244c	11/7/18	Stuart Highway, Adelaide River	13º13'43.20"S	131º06'03.08"E	Seasonal causeway	1511.9	6.06639	Charred at 500ºC for 2 hours	ABA	-29.3
**SI5655**	245a	11/7/18	Stuart Highway, Adelaide River	13º13'43.86"S	131º06'02.98"E	Seasonal causeway	1511.9	6.06639	Charred at 500ºC for 2 hours	ABA	-28.3
**SI5656**	245b	11/7/18	Stuart Highway, Adelaide River	13º13'43.86"S	131º06'02.98"E	Seasonal causeway	1511.9	6.06639	Charred at 500ºC for 2 hours	ABA	-28.1
**SI5657**	245c	11/7/18	Stuart Highway, Adelaide River	13º13'43.86"S	131º06'02.98"E	Seasonal causeway	1511.9	6.06639	Charred at 500ºC for 2 hours	ABA	-28.1
**SI5658**	246a	11/7/18	Stuart Highway, Adelaide River	13º13'45.14"S	131º06'03.24"E	Seasonal causeway	1511.9	6.06639	Charred at 500ºC for 2 hours	ABA	-29.8
**SI5659**	246b	11/7/18	Stuart Highway, Adelaide River	13º13'45.14"S	131º06'03.24"E	Seasonal causeway	1511.9	6.06639	Charred at 500ºC for 2 hours	ABA	-29.4
**SI5660**	246c	11/7/18	Stuart Highway, Adelaide River	13º13'45.14"S	131º06'03.24"E	Seasonal causeway	1511.9	6.06639	Charred at 500ºC for 2 hours	ABA	-29.3
**SI5661**	247a	11/7/18	Stuart Highway, Grove Hill	13º24'47.89"S	131º17'43.32"E	Floodplain fringe	1425.7	6.00714	Charred at 500ºC for 2 hours	ABA	-29.5
**SI5662**	247b	11/7/18	Stuart Highway, Grove Hill	13º24'47.89"S	131º17'43.32"E	Floodplain fringe	1425.7	6.00714	Charred at 500ºC for 2 hours	ABA	-29.5
**SI5663**	247c	11/7/18	Stuart Highway, Grove Hill	13º24'47.89"S	131º17'43.32"E	Floodplain fringe	1425.7	6.00714	Charred at 500ºC for 2 hours	ABA	-29.3
**SI5664**	248a	11/7/18	Stuart Highway, Grove Hill	13º24'47.98"S	131º17'43.38"E	Floodplain fringe	1425.7	6.00714	Charred at 500ºC for 2 hours	ABA	-28.3
**SI5665**	248b	11/7/18	Stuart Highway, Grove Hill	13º24'47.98"S	131º17'43.38"E	Floodplain fringe	1425.7	6.00714	Charred at 500ºC for 2 hours	ABA	-28.4
**SI5666**	248c	11/7/18	Stuart Highway, Grove Hill	13º24'47.98"S	131º17'43.38"E	Floodplain fringe	1425.7	6.00714	Charred at 500ºC for 2 hours	ABA	-28.2

**SI5667**	249a	11/7/18	Stuart Highway, Grove Hill	13º24'48.32"S	131º17'46.67"E	Floodplain fringe	1425.7	6.00714	Charred at 500ºC for 2 hours	ABA	-29.7
**SI5668**	249b	11/7/18	Stuart Highway, Grove Hill	13º24'48.32"S	131º17'46.67"E	Floodplain fringe	1425.7	6.00714	Charred at 500ºC for 2 hours	ABA	-29.6
**SI5669**	249c	11/7/18	Stuart Highway, Grove Hill	13º24'48.32"S	131º17'46.67"E	Floodplain fringe	1425.7	6.00714	Charred at 500ºC for 2 hours	ABA	-29.3
**SI5670**	250a	11/7/18	Stuart Highway, Hayes Creek	13º34'13.00"S	131º26'26.68"E	Open forest and woodland	1373.36	5.99549	Charred at 500ºC for 2 hours	ABA	-30.0
**SI5671**	250b	11/7/18	Stuart Highway, Hayes Creek	13º34'13.00"S	131º26'26.68"E	Open forest and woodland	1373.36	5.99549	Charred at 500ºC for 2 hours	ABA	-29.7
**SI5672**	250c	11/7/18	Stuart Highway, Hayes Creek	13º34'13.00"S	131º26'26.68"E	Open forest and woodland	1373.36	5.99549	Charred at 500ºC for 2 hours	ABA	-29.8
**SI5673**	251a	11/7/18	Stuart Highway, Hayes Creek	13º34'11.60"S	131º28'23.29"E	Monsoon vine forest	1376.22	5.99877	Charred at 500ºC for 2 hours	ABA	-30.0
**SI5674**	251b	11/7/18	Stuart Highway, Hayes Creek	13º34'11.60"S	131º28'23.29"E	Monsoon vine forest	1376.22	5.99877	Charred at 500ºC for 2 hours	ABA	-30.0
**SI5675**	251c	11/7/18	Stuart Highway, Hayes Creek	13º34'11.60"S	131º28'23.29"E	Monsoon vine forest	1376.22	5.99877	Charred at 500ºC for 2 hours	ABA	-30.0
**SI5676**	252a	12/7/18	Stuart Highway, Hayes Creek	13º34'11.46"S	131º28'22.76"E	Monsoon vine forest	1376.22	5.99877	Charred at 500ºC for 2 hours	ABA	-29.5
**SI5677**	253a	12/7/18	Stuart Highway, Emerald Springs	13º39'04.27"S	131º40'01.77"E	Open forest and woodland	1391.78	6.04095	Charred at 500ºC for 2 hours	ABA	-29.6
**SI5678**	253b	12/7/18	Stuart Highway, Emerald Springs	13º39'04.27"S	131º40'01.77"E	Open forest and woodland	1391.78	6.04095	Charred at 500ºC for 2 hours	ABA	-29.3
**SI5679**	253c	12/7/18	Stuart Highway, Emerald Springs	13º39'04.27"S	131º40'01.77"E	Open forest and woodland	1391.78	6.04095	Charred at 500ºC for 2 hours	ABA	-29.7

**SI5680**	254a	12/7/18	Stuart Highway, Emerald Springs	13º41'14.25"S	131º41'38.73"E	Floodplain fringe	1396.15	6.05148	Charred at 500ºC for 2 hours	ABA	-30.6
**SI5681**	254b	12/7/18	Stuart Highway, Emerald Springs	13º41'14.25"S	131º41'38.73"E	Floodplain fringe	1396.15	6.05148	Charred at 500ºC for 2 hours	ABA	-30.4
**SI5682**	254c	12/7/18	Stuart Highway, Emerald Springs	13º41'14.25"S	131º41'38.73"E	Floodplain fringe	1396.15	6.05148	Charred at 500ºC for 2 hours	ABA	-30.6
**SI5683**	255a	12/7/18	Stuart Highway, Emerald Springs	13º41'12.07"S	131º41'41.04"E	Floodplain fringe	1396.15	6.05148	Charred at 500ºC for 2 hours	ABA	-30.9
**SI5684**	255b	12/7/18	Stuart Highway, Emerald Springs	13º41'12.07"S	131º41'41.04"E	Floodplain fringe	1396.15	6.05148	Charred at 500ºC for 2 hours	ABA	-31.2
**SI5685**	255c	12/7/18	Stuart Highway, Emerald Springs	13º41'12.07"S	131º41'41.04"E	Floodplain fringe	1396.15	6.05148	Charred at 500ºC for 2 hours	ABA	-30.8
**SI5686**	256a	12/7/18	Stuart Highway, Pine Creek	13º48'51.68"S	131º50'06.74"E	Seasonal causeway	1423.02	6.09988	Charred at 500ºC for 2 hours	ABA	-29.0
**SI5687**	256b	12/7/18	Stuart Highway, Pine Creek	13º48'51.68"S	131º50'06.74"E	Seasonal causeway	1423.02	6.09988	Charred at 500ºC for 2 hours	ABA	-28.9
**SI5688**	256c	12/7/18	Stuart Highway, Pine Creek	13º48'51.68"S	131º50'06.74"E	Seasonal causeway	1423.02	6.09988	Charred at 500ºC for 2 hours	ABA	-28.8
**SI5689**	257a	12/7/18	Stuart Highway, Katherine	14º21'42.47"S	132º08'25.65"E	Open forest and woodland	1029.25	5.02488	Charred at 500ºC for 2 hours	ABA	-25.9
**SI5690**	257b	12/7/18	Stuart Highway, Katherine	14º21'42.47"S	132º08'25.65"E	Open forest and woodland	1029.25	5.02488	Charred at 500ºC for 2 hours	ABA	-26.3
**SI5691**	257c	12/7/18	Stuart Highway, Katherine	14º21'42.47"S	132º08'25.65"E	Open forest and woodland	1029.25	5.02488	Charred at 500ºC for 2 hours	ABA	-26.8

**SI5692**	258a	12/7/18	Stuart Highway, Katherine	14º21'41.75"S	132º08'24.41"E	Open forest and woodland	1029.25	5.02488	Charred at 500ºC for 2 hours	ABA	-29.0
**SI5693**	258b	12/7/18	Stuart Highway, Katherine	14º21'41.75"S	132º08'24.41"E	Open forest and woodland	1029.25	5.02488	Charred at 500ºC for 2 hours	ABA	-28.9
**SI5694**	259a	12/7/18	Stuart Highway, Katherine	14º21'40.54"S	132º08'20.83"E	Open forest and woodland	1029.25	5.02488	Charred at 500ºC for 2 hours	ABA	-27.3
**SI5695**	259b	12/7/18	Stuart Highway, Katherine	14º21'40.54"S	132º08'20.83"E	Open forest and woodland	1029.25	5.02488	Charred at 500ºC for 2 hours	ABA	-27.2
**SI5696**	260a	12/7/18	Stuart Highway, Edith	14º17'46.17"S	132º04'57.14"E	Open forest and woodland	1077.33	5.2134	Charred at 500ºC for 2 hours	ABA	-28.2
**SI5697**	260b	12/7/18	Stuart Highway, Edith	14º17'46.17"S	132º04'57.14"E	Open forest and woodland	1077.33	5.2134	Charred at 500ºC for 2 hours	ABA	-28.1
**SI5698**	260c	12/7/18	Stuart Highway, Edith	14º17'46.17"S	132º04'57.14"E	Open forest and woodland	1077.33	5.2134	Charred at 500ºC for 2 hours	ABA	-28.5
**SI5699**	261a	12/7/18	Stuart Highway, Edith	14º17'46.51"S	132º04'58.13"E	Open forest and woodland	1077.33	5.2134	Charred at 500ºC for 2 hours	ABA	-29.0
**SI5700**	261b	12/7/18	Stuart Highway, Edith	14º17'46.51"S	132º04'58.13"E	Open forest and woodland	1077.33	5.2134	Charred at 500ºC for 2 hours	ABA	-29.0
**SI5701**	261c	12/7/18	Stuart Highway, Edith	14º17'46.51"S	132º04'58.13"E	Open forest and woodland	1077.33	5.2134	Charred at 500ºC for 2 hours	ABA	-29.2
**SI5702**	262a	12/7/18	Stuart Highway, Edith	14º12'51.99"S	132º02'20.28"E	Open forest and woodland	1135.29	5.43953	Charred at 500ºC for 2 hours	ABA	-28.5
**SI5703**	263a	12/7/18	Stuart Highway, Between Edith and Pine Creek	14º01'38.74"S	131º56'21.69"E	Open forest and woodland	1319.32	5.92887	Charred at 500ºC for 2 hours	ABA	-28.7
**SI5704**	263b	12/7/18	Stuart Highway, Between Edith	14º01'38.74"S	131º56'21.69"E	Open forest and woodland	1319.32	5.92887	Charred at 500ºC for 2 hours	ABA	-28.8

			and Pine Creek								
**SI5705**	263c	12/7/18	Stuart Highway, Between Edith and Pine Creek	14º01'38.74"S	131º56'21.69"E	Open forest and woodland	1319.32	5.92887	Charred at 500ºC for 2 hours	ABA	-28.8
**SI5706**	264a	12/7/18	Stuart Highway, Between Edith and Pine Creek	14º00'45.18"S	131º55'58.13"E	Open forest and woodland	1337.62	5.95009	Charred at 500ºC for 2 hours	ABA	-29.0
**SI5707**	264b	12/7/18	Stuart Highway, Between Edith and Pine Creek	14º00'45.18"S	131º55'58.13"E	Open forest and woodland	1337.62	5.95009	Charred at 500ºC for 2 hours	ABA	-29.0
**SI5708**	265a	12/7/18	Stuart Highway, Between Edith and Pine Creek	14º00'45.51"S	131º55'58.91"E	Open forest and woodland	1337.62	5.95009	Charred at 500ºC for 2 hours	ABA	-29.1
**SI5709**	265b	12/7/18	Stuart Highway, Between Edith and Pine Creek	14º00'45.51"S	131º55'58.91"E	Open forest and woodland	1337.62	5.95009	Charred at 500ºC for 2 hours	ABA	-29.3
**SI5710**	265c	12/7/18	Stuart Highway, Between Edith and Pine Creek	14º00'45.51"S	131º55'58.91"E	Open forest and woodland	1337.62	5.95009	Charred at 500ºC for 2 hours	ABA	-29.3
**SI5711**	266a	12/7/18	Stuart Highway, Between Edith and Pine Creek	14º00'47.37"S	131º56'00.19"E	Open forest and woodland	1337.62	5.95009	Charred at 500ºC for 2 hours	ABA	-30.3
**SI5712**	266b	12/7/18	Stuart Highway, Between Edith and Pine Creek	14º00'47.37"S	131º56'00.19"E	Open forest and woodland	1337.62	5.95009	Charred at 500ºC for 2 hours	ABA	-30.1

**SI5713**	266c	12/7/18	Stuart Highway, Between Edith and Pine Creek	14º00'47.37"S	131º56'00.19"E	Open forest and woodland	1337.62	5.95009	Charred at 500ºC for 2 hours	ABA	-30.1
**SI5714**	267a	12/7/18	Stuart Highway, Pine Creek	13º50'51.83"S	131º50'23.94"E	Open forest and woodland	1422.16	6.09845	Charred at 500ºC for 2 hours	ABA	-30.5
**SI5715**	267b	12/7/18	Stuart Highway, Pine Creek	13º50'51.83"S	131º50'23.94"E	Open forest and woodland	1422.16	6.09845	Charred at 500ºC for 2 hours	ABA	-30.4
**SI5716**	267c	12/7/18	Stuart Highway, Pine Creek	13º50'51.83"S	131º50'23.94"E	Open forest and woodland	1422.16	6.09845	Charred at 500ºC for 2 hours	ABA	-29.8
**SI5717**	268a	12/7/18	Stuart Highway, Pine Creek	13º47'01.14"S	131º47'27.17"E	Open forest and woodland	1419.62	6.09440	Charred at 500ºC for 2 hours	ABA	-28.3
**SI5718**	268b	12/7/18	Stuart Highway, Pine Creek	13º47'01.14"S	131º47'27.17"E	Open forest and woodland	1419.62	6.09440	Charred at 500ºC for 2 hours	ABA	-28.3
**SI5719**	268c	12/7/18	Stuart Highway, Pine Creek	13º47'01.14"S	131º47'27.17"E	Open forest and woodland	1419.62	6.09440	Charred at 500ºC for 2 hours	ABA	-28.2
**SI5720**	269a	12/7/18	Stuart Highway, Pine Creek	13º47'02.40"S	131º47'27.60"E	Open forest and woodland	1419.62	6.09440	Charred at 500ºC for 2 hours	ABA	-29.7
**SI5721**	269b	12/7/18	Stuart Highway, Pine Creek	13º47'02.40"S	131º47'27.60"E	Open forest and woodland	1419.62	6.09440	Charred at 500ºC for 2 hours	ABA	-29.8
**SI5722**	269c	12/7/18	Stuart Highway, Pine Creek	13º47'02.40"S	131º47'27.60"E	Open forest and woodland	1419.62	6.09440	Charred at 500ºC for 2 hours	ABA	-29.5
**SI5723**	270a	12/7/18	Stuart Highway, Pine Creek	13º46'49.60"S	131º47'22.94"E	Open forest and woodland	1419.62	6.09440	Charred at 500ºC for 2 hours	ABA	-29.0
**SI5724**	270b	12/7/18	Stuart Highway, Pine Creek	13º46'49.60"S	131º47'22.94"E	Open forest and woodland	1419.62	6.09440	Charred at 500ºC for 2 hours	ABA	-29.4
**SI5725**	270c	12/7/18	Stuart Highway, Pine Creek	13º46'49.60"S	131º47'22.94"E	Open forest and woodland	1419.62	6.09440	Charred at 500ºC for 2 hours	ABA	-29.3

SI Section 4: Archaeological *Pandanus spiralis* samples

Supplementary Table 4: Archaeological *P. spiralis* isotope samples. DNS: Sample did not survive pretreatment; IS: Insufficient sample survived for analysis following pre-treatment. *Due to small sample size the δ13C value is the result of one analysis only.

ANSTO ID	Sample ID	Phase	Context	δ13CV-PDB (‰)	Number of replicates analysed	Adjusted δ13CV-PDB (‰)
**SI5327**	MJB1	7b	C3/4	-27.9	2	-29.5
**SI5328**	MJB2	7b	C3/4	-26.6	2	-28.2
**SI5329**	MJB3	7b	C3/4	-26.5	2	-28.1
**SI5330**	MJB4	7b	C3/4	-25.8	2	-27.4
**SI5331**	MJB5	7b	C3/4	-24.5	2	-26.1
**SI5332**	MJB6	7b	C3/4	-25.2	2	-26.8
**SI5333**	MJB7	7b	C3/4B	-25.4	2	-27.0
**SI5334**	MJB8	7b	C3/4B	-28.5	2	-30.1
**SI5335**	MJB9	7b	C3/4B	-26.6	2	-28.2
**SI5336**	MJB10	7b	C3/4B	-26.5	2	-28.1
**SI5337**	MJB11	7b	C3/4B	-25.1	2	-26.7
**SI5338**	MJB12	7b	C3/4B	-26.8	2	-28.4
**SI5339**	MJB13	7b	C3/4B	-25.1	2	-26.7
**SI5340**	MJB14	7b	C3/4B	-27.2	2	-28.8
**SI5341**	MJB15	7b	C3/4B	-27.5	2	-29.1
**SI5342**	MJB16	7a	C3/7	-24.1	2	-26.4
**SI5343**	MJB17	5	C3/20	-23.0	2	-25.4
**SI5344**	MJB18	5	C3/20	-22.9	2	-25.3
**SI5345**	MJB19	5	C3/22	-25.0	2	-27.4
**SI5346**	MJB20	4	C2/27B	-24.7	6	-27.5
**SI5347**	MJB21	4	C2/27B	-25.7	2	-28.5
**SI5348**	MJB22	4	C2/27B	DNS	NA	NA
**SI5349**	MJB23	4	C2/27B	-25.2	2	-28.0
**SI5350**	MJB24	4	C2/27B	IS	NA	NA
**SI5351**	MJB25	4	C2/27B	DNS	NA	NA
**SI5352**	MJB26	4	C2/27B	DNS	NA	NA
**SI5353**	MJB27	4	C2/27B	IS	NA	NA
**SI5354**	MJB28	4	C2/27B	DNS	NA	NA
**SI5355**	MJB29	4	C2/28	DNS	NA	NA
**SI5356**	MJB30	4	C2/28	DNS	NA	NA
**SI5357**	MJB31	4	C2/28	IS	NA	NA
**SI5358**	MJB32	4	C2/28	DNS	NA	NA
**SI5359**	MJB33	4	C2/28A	-25.5	2	-28.3
**SI5360**	MJB34	4	C2/28A	-25.7	2	-28.5
**SI5361**	MJB35	4	C2/28A	-25.6	2	-28.4

**SI5362**	MJB36	4	C2/28A	DNS	NA	NA
**SI5363**	MJB37	4	C2/28A	IS	NA	NA
**SI5364**	MJB38	4	C2/29	DNS	NA	NA
**SI5365**	MJB39	4	C2/29	DNS	NA	NA
**SI5366**	MJB40	4	C2/29	DNS	NA	NA
**SI5367**	MJB41	4	C2/29	DNS	NA	NA
**SI5368**	MJB42	4	C2/29	DNS	NA	NA
**SI5369**	MJB43	4	C2/29	DNS	NA	NA
**SI5370**	MJB44	4	C2/29A	DNS	NA	NA
**SI5371**	MJB45	4	C2/29A	-25.4	2	-28.2
**SI5372**	MJB46	3b	C2/30	IS	NA	NA
**SI5373**	MJB47	3b	C2/32A	-23.0	6	-25.8
**SI5374**	MJB48	3b	C2/32A	-24.1	2	-26.9
**SI5375**	MJB49	3b	C2/32A	IS	NA	NA
**SI5376**	MJB50	3b	C2/32A	DNS	NA	NA
**SI5377**	MJB51	3b	C2/32A	-24.3	2	-27.1
**SI5378**	MJB52	3b	C2/32A	-25.7	2	-28.5
**SI5379**	MJB53	3b	C2/32A	DNS	NA	NA
**SI5380**	MJB54	3b	C2/32A	-25.1	2	-27.9
**SI5381**	MJB55	3a	C2/33	DNS	NA	NA
**SI5382**	MJB56	3a	C2/33	DNS	NA	NA
**SI5383**	MJB57	3a	C2/34	DNS	NA	NA
**SI5384**	MJB58	3a	C2/34	DNS	NA	NA
**SI5385**	MJB59	3a	C2/34	IS	NA	NA
**SI5386**	MJB60	3a	C2/34	DNS	NA	NA
**SI5387**	MJB61	3a	C2/34	IS	NA	NA
**SI5388**	MJB62	3a	C2/34	DNS	NA	NA
**SI5389**	MJB63	3a	C2/34	DNS	NA	NA
**SI5390**	MJB64	3a	C2/34	DNS	NA	NA
**SI5391**	MJB65	3a	C2/34	DNS	NA	NA
**SI5392**	MJB66	3a	C2/34	DNS	NA	NA
**SI5393**	MJB67	3a	C2/34A	-24.0	2	-26.8
**SI5394**	MJB68	3a	C2/34A	-24.8	2	-27.6
**SI5395**	MJB69	3a	C2/34A	DNS	NA	NA
**SI5396**	MJB70	3a	C2/34A	-26.2	2	-29.0
**SI5397**	MJB71	3a	C2/34A	-25.7	2	-28.5
**SI5398**	MJB72	3a	C2/34A	DNS	NA	NA
**SI5399**	MJB73	3a	C2/34A	-24.7	1*	-27.5
**SI5400**	MJB74	3a	C2/35	IS	NA	NA
**SI5401**	MJB75	3a	C2/35	-23.1	2	-25.9
**SI5402**	MJB76	3a	C2/35	-24.9	2	-27.7
**SI5403**	MJB77	3a	C2/35	DNS	NA	NA

**SI5404**	MJB78	3a	C2/35B	DNS	NA	NA
**SI5405**	MJB79	3a	C2/35B	DNS	NA	NA
**SI5406**	MJB80	3a	C2/36A	-23.9	2	-26.7
**SI5407**	MJB81	3a	C2/36A	-24.6	1*	-27.4
**SI5408**	MJB82	3a	C2/36A	DNS	NA	NA
**SI5726**	MJB83	2	C2/41	DNS	NA	NA
**SI5727**	MJB84	2	C2/40	DNS	NA	NA
**SI5728**	MJB85	2	C2/39A	-26.9	2	-29.3
**SI5729**	MJB86	2	C2/39A	DNS	NA	NA
**SI5730**	MJB87	2	C2/39A	DNS	NA	NA
**SI5731**	MJB88	2	C2/39A	DNS	NA	NA
**SI5732**	MJB89	2	C2/39A	IS	NA	NA
**SI5734**	MJB91	2	C2/38	-25.9	2	-28.3
**SI5735**	MJB92	2	C2/38	DNS	NA	NA
**SI5736**	MJB93	2	C2/38	DNS	NA	NA
**SI5737**	MJB94	2	C2/37	DNS	NA	NA
**SI5738**	MJB95	2	C2/37	-24.2	2	-26.6
**SI5739**	MJB96	3a	C2/36A	-24.0	3	-26.8
**SI5740**	MJB97	3a	C2/36A	-24.8	3	-27.6
**SI5741**	MJB98	3a	C2/36	-25.9	3	-28.7
**SI5742**	MJB99	3a	C2/36	DNS	NA	NA
**SI5745**	MJB102	3a	C2/35B	-25.9	3	-28.7
**SI5761**	MJB118	3a	C2/35A	-24.0	2	-26.8
**SI5762**	MJB119	3a	C2/35A	-24.3	2	-27.1
**SI5763**	MJB120	3a	C2/35A	-24.3	2	-27.1
**SI5764**	MJB121	3a	C2/35A	IS	NA	NA
**SI5765**	MJB122	3a	C2/35A	-25.2	2	-28.0
**SI5768**	MJB125	3a	C2/35	-24.3	2	-27.1
**SI5769**	MJB126	3a	C2/35	IS	NA	NA
**SI5771**	MJB128	3a	C2/34A	IS	NA	NA
**SI5799**	MJB156	3a	C2/34A	-24.9	3	-27.7
**SI5800**	MJB157	3a	C2/34A	-24.7	2	-27.5
**SI5801**	MJB158	3a	C2/34A	-24.1	2	-26.9
**SI5802**	MJB159	3a	C2/34A	-24.9	2	-27.7
**SI5803**	MJB160	3a	C2/34A	-25.4	1*	-28.2
**SI5804**	MJB161	3a	C2/34A	DNS	NA	NA
**SI5805**	MJB162	3a	C2/34A	-24.5	1*	-27.3
**SI5806**	MJB163	3a	C2/34A	DNS	NA	NA
**SI5807**	MJB164	3a	C2/34A	IS	NA	NA
**SI5808**	MJB165	3a	C2/34A	IS	NA	NA
**SI5809**	MJB166	3a	C2/34A	-26.1	1*	-28.9
**SI5810**	MJB167	3a	C2/34A	IS	NA	NA

**SI5811**	MJB168	3a	C2/34A	IS	NA	NA
**SI5812**	MJB169	3a	C2/34A	-25.5	4	-28.3
**SI5813**	MJB170	3a	C2/34A	IS	NA	NA
**SI5814**	MJB171	3a	C2/34A	-24.9	4	-27.7
**SI5815**	MJB172	3a	C2/34A	DNS	NA	NA
**SI5856**	MJB179	3a	C2/48	IS	NA	NA
**SI5870**	MJB193	3a	C2/34	IS	NA	NA
**SI5871**	MJB194	3a	C2/34	DNS	NA	NA
**SI5872**	MJB195	3a	C2/34	DNS	NA	NA
**SI5873**	MJB196	3a	C2/34	DNS	NA	NA
**SI5874**	MJB197	3a	C2/34	IS	NA	NA
**SI5875**	MJB198	3a	C2/34	IS	NA	NA
**SI5876**	MJB199	3a	C2/33A	IS	NA	NA
**SI5877**	MJB200	3a	C2/33A	-24.8	2	-27.6
**SI5878**	MJB201	3a	C2/33A	IS	NA	NA
**SI5879**	MJB202	3a	C2/33A	-24.6	2	-27.4
**SI5880**	MJB203	3a	C2/33A	-24.8	2	-27.6
**SI5881**	MJB204	3a	C2/33A	-24.5	2	-27.3
**SI5882**	MJB205	3a	C2/33A	-24.9	2	-27.7
**SI5883**	MJB206	3a	C2/33A	IS	NA	NA
**SI5884**	MJB207	3a	C2/33A	-24.6	2	-27.4
**SI5885**	MJB208	3a	C2/33A	-24.2	6	-27.0
**SI5886**	MJB209	3a	C2/33A	-26.3	2	-29.1
**SI5887**	MJB210	3a	C2/33A	-26.4	2	-29.2
**SI5907**	MJB230	3a	C2/33	IS	NA	NA
**SI5908**	MJB231	3a	C2/33	-25.0	2	-27.8
**SI5909**	MJB232	3a	C2/33	DNS	NA	NA
**SI5915**	MJB238	3b	C2/32A	-25.2	2	-28.0
**SI5916**	MJB239	3b	C2/32A	IS	NA	NA
**SI5917**	MJB240	3b	C2/32A	IS	NA	NA
**SI5939**	MJB262	3b	C2/32A	-24.7	2	-27.5
**SI5940**	MJB263	3b	C2/32A	-24.4	2	-27.2
**SI5941**	MJB264	3b	C2/32A	IS	NA	NA
**SI5942**	MJB265	3b	C2/32A	-24.6	2	-27.4
**SI5943**	MJB266	3b	C2/32A	IS	NA	NA
**SI5944**	MJB267	3b	C2/32A	-24.4	2	-27.2
**SI5945**	MJB268	3b	C2/32A	-24.2	2	-27.0
**SI5946**	MJB269	3b	C2/31A	-24.3	2	-27.1
**SI5971**	MJB294	3b	C2/31	-26.0	2	-28.8
**SI5972**	MJB295	3b	C2/31	DNS	NA	NA
**SI5995**	MJB318	3b	C2/30B	DNS	NA	NA
**SI6011**	MJB334	4	C2/29A	IS	NNA	NA

**SI6036**	MJB359	4	C2/29	DNS	NA	NA
**SI6038**	MJB361	4	C2/29	DNS	NA	NA
**SI6039**	MJB362	4	C2/29	DNS	NA	NA
**SI6040**	MJB363	4	C2/28A	-25.6	2	-28.4
**SI6041**	MJB364	4	C2/28A	-25.4	2	-28.2
**SI6042**	MJB365	4	C2/28A	-25.4	2	-28.2
**SI6043**	MJB366	4	C2/28A	-25.6	2	-28.4
**SI6044**	MJB367	4	C2/28A	-24.1	2	-26.9
**SI6045**	MJB368	4	C2/28A	-25.1	2	-27.9
**SI6046**	MJB369	4	C2/28A	-25.6	2	-28.4
**SI6047**	MJB370	4	C2/28A	-25.1	2	-27.9
**SI6048**	MJB371	4	C2/28A	IS	NA	NA
**SI6049**	MJB372	4	C2/28A	-24.9	1*	-27.7
**SI6050**	MJB373	4	C2/28A	-25.6	2	-28.4
**SI6051**	MJB374	4	C2/28A	DNS	NA	NA
**SI6052**	MJB375	4	C2/28A	-26.4	2	-29.2
**SI6053**	MJB376	4	C2/28A	DNS	NA	NA
**SI6054**	MJB377	4	C2/28A	-25.2	2	-28.0
**SI6055**	MJB378	4	C2/28A	IS	NA	NA
**SI6060**	MJB383	4	C2/28	DNS	NA	NA
**SI6061**	MJB384	4	C2/28	DNS	NA	NA
**SI6062**	MJB385	4	C2/28	DNS	NA	NA
**SI6063**	MJB386	4	C2/28	DNS	NA	NA
**SI6064**	MJB387	4	C2/28	-25.9	1*	-28.7
**SI6065**	MJB388	4	C2/28	DNS	NA	NA
**SI6066**	MJB389	4	C2/27A	-26.4	2	-29.2
**SI6067**	MJB390	4	C2/27A	DNS	NA	NA
**SI6068**	MJB391	4	C2/27A	DNS	NA	NA
**SI6069**	MJB392	4	C2/27A	DNS	NA	NA
**SI6070**	MJB393	4	C2/27	DNS	NA	NA
**SI6071**	MJB394	4	C2/25	IS	NA	NA
**SI6072**	MJB395	4	E4/22A	DNS	NA	NA
**SI6073**	MJB396	7a	C3/6	-27.2	2	-29.5
**SI6074**	MJB397	7a	C3/6	-27.5	4	-29.8
**SI6075**	MJB398	7a	C3/6	-26.6	2	-28.9
**SI6076**	MJB399	7a	C3/6	-26.0	2	-28.3
**SI6077**	MJB400	7a	C3/6	-27.5	2	-29.8
**SI6078**	MJB401	7a	C3/6	-25.4	2	-27.7
**SI6079**	MJB402	7b	C3/4	-25.6	2	-27.2
**SI6080**	MJB403	7b	C3/4	-24.6	2	-26.2
**SI6081**	MJB404	7b	C3/4	-25.6	2	-27.2
**SI6082**	MJB405	7b	C3/4	-25.0	6	-26.6

**SI6084**	MJB407	7b	C3/4	-25.6	2	-27.2
**SI6085**	MJB408	7b	C3/4	-25.4	2	-27.0
**SI6086**	MJB409	7b	C3/4	-26.9	2	-28.5
**SI6087**	MJB410	7b	C3/4	-27.2	2	-28.8
**SI6088**	MJB411	7b	C3/4	-25.6	2	-27.2
**SI6089**	MJB412	7b	C3/4	-27.0	2	-28.6
**SI6090**	MJB413	7b	C3/4	-25.8	2	-27.4
**SI6091**	MJB414	7b	C3/4	-24.9	2	-26.5
**SI6092**	MJB415	7b	C3/4	-26.6	2	-28.2
**SI6093**	MJB416	7b	C3/4	-26.1	8	-27.7
**SI6094**	MJB417	7b	C3/2	-27.9	2	-29.5
**SI6095**	MJB418	7b	C3/2	-26.4	2	-28.0
**SI6096**	MJB419	7b	C3/2	-25.7	2	-27.3
**SI6097**	MJB420	7b	C3/2	-26.8	3	-28.4
**SI6098**	MJB421	7b	C3/2	-27.0	2	-28.6
**SI6099**	MJB422	7b	C3/2	-25.7	2	-27.3
**SI6100**	MJB423	7b	C3/2	-26.5	2	-28.1
**SI6101**	MJB424	7b	C3/2	-25.4	2	-27.0
**SI6102**	MJB425	7b	C3/2	-27.8	2	-29.4
**SI6103**	MJB426	7b	C3/2	-25.6	2	-27.2
**SI6104**	MJB427	7a	B3/5A	-26.2	2	-28.5
**SI6105**	MJB428	7b	E4/6A	DNS	NA	NA
**SI6106**	MJB429	7b	C3/4A	-25.3	2	-26.9
**SI6107**	MJB430	7b	C3/4A	-26.1	2	-27.7
**SI6108**	MJB431	7b	C3/4A	-26.1	3	-27.7
**SI6109**	MJB432	7b	C3/4A	-24.5	2	-26.1
**SI6110**	MJB433	7b	C3/4A	-25.9	2	-27.5
**SI6111**	MJB434	7b	C3/4A	NS	NA	NA
**SI6112**	MJB435	7b	C3/4A	-25.0	2	-26.6
**SI6113**	MJB436	7b	C3/4A	DNS	NA	NA
**SI6114**	MJB437	7b	C3/4A	-26.6	3	-28.2
**SI6115**	MJB438	7b	C3/4A	-27.9	2	-29.5
**SI6116**	MJB439	7b	C3/4A	-25.3	4	-26.9
**SI6117**	MJB440	7b	C3/4A	-25.2	2	-26.8
**SI6118**	MJB441	7b	C3/4A	-25.9	2	-27.5
**SI6119**	MJB442	7b	C3/4A	IS	NA	NA
**SI6120**	MJB443	7b	C3/4A	-26.1	2	-27.7
**SI6121**	MJB444	7b	C3/4A	-26.1	2	-27.7
**SI6122**	MJB445	7b	C3/4A	-26.8	2	-28.4
**SI6123**	MJB446	7b	C3/4A	-24.2	2	-25.8
**SI6124**	MJB447	7b	C3/4A	-26.2	2	-27.8

SI Section 5: Isotope standards

The isotope samples reported on in this paper were analysed in 5 separate batches and 37 runs. The below tables provide the Isotope standards for each of these batches.

Supplementary Table 5: Isotope standards for SI3790-SI3949. Samples were analysed across 15 runs on an Elementar VarioMICRO Elemental Analyser (EA) and an IsoPrime Continuous-Flow Isotope Ratio Mass Spectrometer (CF-IRMS).

Standard	Mean δ13CV-PDB (‰)	Standard Deviation	Certified δ13CV-PDB (‰)	Difference	No. of Replicates
**EM B2155**	-26.92	0.23	-26.98	0.06	21
**EM B2151**	-25.86	0.23	-26.07	0.21	21

Supplementary Table 6: Isotope standards for SI4396-SI4443. Samples were analysed across 6 runs on an Elementar VarioMICRO EA and an IsoPrime CF-IRMS.

Standard	Mean δ13CV-PDB (‰)	Standard Deviation	Certified δ13CV-PDB (‰)	Difference	No. of Replicates
**EM B2155**	-26.92	0.14	-26.98	0.06	11
**EM B2151**	-26.07	0.12	-26.27	0.2	9

Supplementary Table 7: Isotope standards for SI5263-SI5408. Samples were analysed across 4 runs on an Elementar VarioMICRO EA and an IsoPrime CF-IRMS.

Standard	Mean δ13CV-PDB (‰)	Standard Deviation	Certified δ13CV-PDB (‰)	Difference	No. of Replicates
**EM B2155**	-27.02	0.18	-26.98	-0.04	10
**EM B2151**	-26.12	0.17	-26.27	0.15	10

Supplementary Table 8: Isotope standards for SI 5616-SI5821. Samples were analysed across 5 runs on an Elementar VarioMICRO EA and an IsoPrime CF-IRMS.

Standard	Mean δ13CV-PDB (‰)	Standard Deviation	Certified δ13CV-PDB (‰)	Difference	No. of Replicates
**EM B2155**	-27	0.21	-26.98	-0.02	22

**EM B2151**	-26.17	0.16	-26.27	0.1	22

Supplementary Table 9: Isotope standards for SI5856-SI6124. Samples were analysed across 7 runs on an Elementar VarioMICRO EA and an IsoPrime CF-IRMS.

Standard	Mean δ13CV-PDB (‰)	Standard Deviation	Certified δ13CV-PDB (‰)	Difference	No. of Replicates
**EM B2155**	-26.99	0.17	-26.98	-0.01	32
**EM B2151**	-26.07	0.13	-26.27	0.2	33

SI Section 6: AMS Radiocarbon dates

Supplementary Table 10: AMS radiocarbon dates. Note: Age calibration was performed using the SHCal13 data[11] and OxCal program[12].

Lab ID	Sample Type	Sample ID	^{14}C Age (BP)	Calibrated ^{14}C Age (cal. BP)	
				95% confidence level	Median
**OZQ464**	Charcoal	C14X2-C3/4	145 ± 20	259 - 0	94
**OZX661**	Charcoal	C3/5(HR1)	690 ± 20	660 - 559	599
**OZX662**	Charcoal	C3/7(HR1)	710 ± 20	665 - 564	602

SI Section 7: Soil samples

Soil isotope samples were taken approximately every 5cm below surface from the south wall of Square B2 from 0.07m to 3.2 m depth. Soil sample depths were estimated from a tape measure fixed to the wall and tied into the site datum at each metre mark using a total station. Correction for slope in the wall and tape measure, calculated from the total station data, means the sample depths used in this study differ slightly from those reported in Clarkson et al.[13]. This data is online at at https://osf.io/9tn4f/.

SI Section 8: Comparison of the Madjedbebe pandanus palaeoprecipitation proxy to other Australian environmental records

Modern moisture in northern Australia is closely related to the strength of the summer monsoon. This pattern is maintained through the whole of the last glacial cycle, with the monsoon waxing and waning in tandem with precessional forcing[14].

Phase 2: *65–52.7 kya.* The highest lake levels in Lake Eyre in the last glacial cycle occurred between 65 and 60 kya[15]. Since Lake Eyre is mostly fed by flows from tropical northern Australia this suggests an effective northern Australian monsoon at this time. However, this does not correspond to a period of strong summer insolation in northern Australia and more recent work on mega-lake Frome[16] now indicates that westerly derived (temperate) moisture may have reached Lake Eyre at this time. There is little direct evidence for rainfall in northern Australia at this time, but the suggestion is that the period is relatively dry.

Phase 3: 51.6–28.1 kya. The period between 50–40 kya (Phase 3a) is represented by strong river flows from the north into the Lake Eyre Basin[17] and along the tropical Queensland coast[18]. Both these records suggest that the tropical monsoon is strong at this time. 40 kya also represents a high stand at Lake Eyre[15] and in this case there is no suggestion of temperate waters from Lake Frome reaching Lake Eyre. Phase 3a appears to be relatively moist in the north. An interstadial moderately high sea-level occurred at c. 50 kya and then sea-levels declined. This would have favoured higher effective moisture during the 50–40 kya period with a declining trend thereafter. The evidence relating to moisture availability between 40 and 30kya is limited but at least some records indicate that the monsoon was active[19].

Phase 4: 26.7–13.2 kya. Orbital conditions favour a strong monsoon at this time but sea-levels at -125 m mean that the coastline is far removed across the northern Australia region, with a brackish lake occupying part of the Gulf of Carpentaria and the shoreline extended hundreds of kilometres to the NW in the Arafura Sea region. Long assumed to be a period of great aridity, recent climate modelling results[20] and speleothem data[21,22] suggest that the northern Australian monsoon was active during the Last Glacial Maximum, 24–20 kya. Recent work from Girraween Lagoon near Darwin indicates that vegetation transitioned to a more open grassy savannah during the Last Glacial Maximum and this was interpreted as reflecting 700-1000 mm of precipitation[23]. These authors interpret the monsoon as active at this time and attribute the changes to increased distance to the coast and reduced CO_2. River flows in the Australian tropics increased after 30 kya and declined after the LGM between 17 and 10 kya[24]. Waterfall plunge pools may have been active during the Last Glacial Maximum[25], although some of this data has been reinterpreted to be younger based on newer OSL ages[26]. Despite all this evidence for an active monsoon, because of the expanded Australian landmass, precipitation gradients were based on a coastline hundreds of kilometres seaward of the present position. This means that at many sites, precipitation was lower than during the Holocene during the Last Glacial Maximum period.

Phase 5: 9.7–8 kya. The modern Northern Australian monsoon is inferred to have restarted at about 14 kya[27,28]. Across most of Australia the early to mid-Holocene had higher effective precipitation than the present day with several records indicating

a maximum between c. 10 kya and 6 kya[29,30]. This pattern is confirmed from the Kimberley[27].

Phase 7: 602 cal BP to present. Overall, the late Holocene in northern Australia is a period of less reliable rainfall and a weaker monsoon than the preceding mid- to early Holocene[29]. The direst phase in the Kimberley precedes Phase 7 at between 2.6 and 1.3 kya[27]. The last few thousand years are not monotonically dry, however. For example, increased fluvial activity in arid central Australia has been recognised to co-eval with the early part of the medieval climatic anomaly in Europe about 900 ya[31]. Records are quite confusing. Yan et al.[20] reviewed monsoon paleo-records from northern Australia and suggested that conditions were arid during the 'Little Ice Age' (defined by them as 1400-1850 AD) and related this to a withdrawal of the Inter-tropical Convergence northward at this time. This is roughly co-eval with a dry phase interpreted from the Kimberley in NW Australia[27]. In contrast, Rouillard et al.[32] recorded evidence for extreme floods in NW Australia during the same period. These records are not incompatible with modern rainfall at high levels in NW Australia due to favourable Indian Ocean Dipole conditions, while NE Australia has suffered repeated poor rainfall years in the last few decades due to ENSO and Pacific Decadal Oscillation Effects. One record appears to align well with our findings, Hendy et al.[33] noted a sharp decrease in salinity at the end of the Little Ice Age in waters off the barrier reef. This is consistent with an uptick in the monsoon roughly coincident with our Phase 7b.

Our overall conclusion is that, given the relatively poor understanding of long-term moisture balances in tropical Australia, the δ13C record presented here is consistent with the current observations and provides a powerful new tool for palaeoprecipitation reconstruction.

Supplementary references

1 Stone, B. C. A guide to collecting Pandanaceae (Pandanus, Freycinetia, and Sararanga). *Ann. Mo. Bot. Gard.* **70**, 137-145 (1983).

2 Northern Territory Government *Flora NT: Northern Territory flora online*, <http://eflora.nt.gov.au/home> (2013).

3 Fox, G. & Garde, M. *An-me Arri-ngun, The Food We Eat: Traditional plant foods of the Kundjeyhmi people of Kakadu National Park*. (Gundjeihmi Aboriginal Corporation, 2018).

4 Lim, T. K. in *Edible Medicinal and Non-medicinal plants* Vol. 4 134-135 (Springer Netherlands, 2012).

5 Verbeek, N. A., Braithwaite, R. W. & Boasson, R. The importance of *Pandanus spiralis* to birds. *Emu* **93**, 53-58, doi:10.1071/MU9930053 (1993).

6 Rasmussen, A., Dobrijevic, D. P., Ola, A., Ishaya, F. D. & Lovelock, C. E. Aerial Root Physiology: Reaching for the Sky or Down to Earth? *Ann. Plant Rev.* **2**, 1-32, doi:10.1002/9781119312994.apr0668 (2019).

7 Hamby, L. *Containers of Power: Women with Clever Hands*. (Utber & Patullo, 2010).

8 Low, T. *Wild Food Plants of Australia*. (Angus & Robertson Publishing, 1991).

9 Meehan, B., Gaffey, P. & Jones, R. in *Readings in Material Culture* Vol. 9 *Occasional papers in anthropology* (ed Peter K. Lauer) 73-96 (Anthropology Museum, University of Queensland, 1978).

10 Hutchinson, M. F., Stein, J. L., Stein, J. A., Anderson, H. & Tickle, P. K. <https://ecat.ga.gov.au/geonetwork/srv/eng/catalog.search#/metadata/66006> (Geosciences Australia, Canberra, 2008).

11 Hogg, A. G. *et al.* SHCal13 southern hemisphere calibration, 0–50,000 years cal BP. *Radiocarbon* **55**, 1889-1903, doi:10.2458/azu_js_rc.55.16783 (2013).

12 Bronk Ramsey, C. Bayesian analysis of radiocarbon dates. *Radiocarbon* **51**, 337-360, doi:10.1017/s0033822200033865 (2009).

13 Clarkson, C. *et al.* Human occupation of northern Australia by 65,000 years ago. *Nature* **547**, 306-310, doi:10.1038/nature22968 (2017).

14 Beaufort, L., van der Kaars, S., Bassinot, F. C. & Moron, V. Past dynamics of the Australian monsoon: precession, phase and links to the global monsoon concept. *Clim. Past* **6**, 695-706, doi:10.5194/cp-6-695-2010 (2010).

15 Magee, J. W., Miller, G. H., Spooner, N. A. & Questiaux, D. Continuous 150 k.y. monsoon record from Lake Eyre, Australia: Insolation-forcing implications and unexpected Holocene failure. *Geol.* **32**, doi:10.1130/g20672.1 (2004).

16 Cohen, T. J. *et al.* Late Quaternary mega-lakes fed by the northern and southern river systems of central Australia: Varying moisture sources and increased continental aridity. *Palaeogeogr., Palaeoclim., Palaeoecol.* **356-357**, 89-108, doi:10.1016/j.palaeo.2011.06.023 (2012).

17 Maroulis, J. C., Nanson, G. C., Price, D. M. & Pietsch, T. Aeolian–fluvial interaction and climate change: source-bordering dune development over the past ~100ka on Cooper Creek, central Australia. *Quat. Sci. Rev.***26**, 386-404, doi:10.1016/j.quascirev.2006.08.010 (2007).

18 Croke, J., Jansen, J. D., Amos, K. & Pietsch, T. J. A 100 ka record of fluvial activity in the Fitzroy River Basin, tropical northeastern Australia. *Quat. Sci. Rev.* **30**, 1681-1695, doi:10.1016/j.quascirev.2011.03.012 (2011).

19 Ward, I. A. K. *et al.* Late Quaternary landscape evolution in the Keep River region, northwestern Australia. *Quat. Sci. Rev.* **24**, 1906-1922, doi:10.1016/j.quascirev.2004.11.004 (2005).
20 Yan, H. *et al.* Dynamics of the intertropical convergence zone over the western Pacific during the Little Ice Age. *Nat. Geosci.* **8**, 315-320, doi:10.1038/ngeo2375 (2015).
21 Denniston, R. F. *et al.* North Atlantic forcing of millennial-scale Indo-Australian monsoon dynamics during the Last Glacial period. *Quat. Sci. Rev.* **72**, 159-168, doi:10.1016/j.quascirev.2013.04.012 (2013).
22 Denniston, R. F. *et al.* A Stalagmite record of Holocene Indonesian–Australian summer monsoon variability from the Australian tropics. *Quat. Sci. Rev.* **78**, 155-168, doi:10.1016/j.quascirev.2013.08.004 (2013).
23 Rowe, C. *et al.* Vegetation over the last glacial maximum at Girraween Lagoon, monsoonal northern Australia. *Quat. Res.*, 1-14, doi:10.1017/qua.2020.50 (2020).
24 Reeves, J. M. *et al.* Palaeoenvironmental change in tropical Australasia over the last 30,000 years – a synthesis by the OZ-INTIMATE group. *Quat. Sci. Rev.* **74**, 97-114, doi:10.1016/j.quascirev.2012.11.027 (2013).
25 Nott, J. & Price, D. Waterfalls, floods and climate change: Evidence from tropical Australia. *Earth and Planet. Sci. Lett.***171**, 267-276 (1999).
26 May, J.-H., Preusser, F. & Gliganic, L. A. Refining late Quaternary plunge pool chronologies in Australia's monsoonal 'Top End'. *Quat. Geochron.* **30**, 328-333, doi:10.1016/j.quageo.2015.01.008 (2015).
27 Field, E., McGowan, H. A., Moss, P. T. & Marx, S. K. A late Quaternary record of monsoon variability in the northwest Kimberley, Australia. *Quat. Int.l* **449**, 119-135, doi:10.1016/j.quaint.2017.02.019 (2017).
28 Wyrwoll, K.-H. & Miller, G. H. Initiation of the Australian summer monsoon 14,000 years ago. *Quat. Int.* **83-85**, 119-128 (2001).
29 Shulmeister, J. & Lees, B. G. Pollen evidence from tropical Australia for the onset of an ENSO-dominated climate at c. 4000 BP. *Holocene* **5**, 10-18, doi:10.1177/095968369500500102 (1995).
30 Woodward, C. *et al.* A Holocene record of climate and hydrological changes from Little Llangothlin Lagoon, south eastern Australia. *Holocene* **24**, 1665-1674, doi:10.1177/0959683614551218 (2014).
31 Cohen, T. J. *et al.* A pluvial episode identified in arid Australia during the Medieval Climatic Anomaly. *Quat. Sci. Rev.* **56**, 167-171, doi:10.1016/j.quascirev.2012.09.021 (2012).
32 Rouillard, A. *et al.* Evidence for extreme floods in arid subtropical northwest Australia during the Little Ice Age chronozone (CE 1400–1850). *Quat. Sci. Rev.* **144**, 107-122, doi:10.1016/j.quascirev.2016.05.004 (2016).
33 Hendy, E. J. *et al.* Abrupt decrease in tropical Pacific sea surface salinity at end of Little Ice Age. *Science* **295**, 1511-1514 (2002).
