# Supplementary material for: Pandanus nutshell generates a palaeoprecipitation record for human occupation at Madjedbebe, northern Australia
Source: Nat Ecol Evol. 2021 Jan 25;5(3):295–303. doi: 10.1038/s41559-020-01379-8 (PMC7929916; doi:10.1038/s41559-020-01379-8)
Supplement: Supplementary file 2 — Reporting Summary [file 41559_2020_1379_MOESM2_ESM.pdf]

nature research

Corresponding author(s): S. Anna Florin

Last updated by author(s): Nov 27, 2020

Reporting Summary

Nature Research wishes to improve the reproducibility of the work that we publish. This form provides structure for consistency and transparency in reporting. For further information on Nature Research policies, see our Editorial Policies and the Editorial Policy Checklist.

Statistics

For all statistical analyses, confirm that the following items are present in the figure legend, table legend, main text, or Methods section.

n/a	Confirmed	
☐	☒	The exact sample size (n) for each experimental group/condition, given as a discrete number and unit of measurement
☐	☒	A statement on whether measurements were taken from distinct samples or whether the same sample was measured repeatedly
☐	☒	The statistical test(s) used AND whether they are one- or two-sided<br>*Only common tests should be described solely by name; describe more complex techniques in the Methods section.*
☐	☒	A description of all covariates tested
☐	☒	A description of any assumptions or corrections, such as tests of normality and adjustment for multiple comparisons
☐	☒	A full description of the statistical parameters including central tendency (e.g. means) or other basic estimates (e.g. regression coefficient) AND variation (e.g. standard deviation) or associated estimates of uncertainty (e.g. confidence intervals)
☐	☒	For null hypothesis testing, the test statistic (e.g. F, t, r) with confidence intervals, effect sizes, degrees of freedom and P value noted<br>*Give P values as exact values whenever suitable.*
☒	☐	For Bayesian analysis, information on the choice of priors and Markov chain Monte Carlo settings
☒	☐	For hierarchical and complex designs, identification of the appropriate level for tests and full reporting of outcomes
☒	☐	Estimates of effect sizes (e.g. Cohen's d, Pearson's r), indicating how they were calculated

Our web collection on statistics for biologists contains articles on many of the points above.

Software and code

Policy information about availability of computer code

Data collection	FileMaker, Garmin Basecamp
Data analysis	R, Excel, ArcGIS, JMP Statistical Software

For manuscripts utilizing custom algorithms or software that are central to the research but not yet described in published literature, software must be made available to editors and reviewers. We strongly encourage code deposition in a community repository (e.g. GitHub). See the Nature Research guidelines for submitting code & software for further information.

Data

Policy information about availability of data

All manuscripts must include a data availability statement. This statement should provide the following information, where applicable:

- Accession codes, unique identifiers, or web links for publicly available datasets
- A list of figures that have associated raw data
- A description of any restrictions on data availability

All elements necessary to allow interpretation and replication of results, including full datasets are provided in the supporting online information. R code and additional data for Fig. 2-4 is online at https://osf.io/9tn4f/. Archaeobotanical material analysed in this study will be kept in the Archaeology Laboratories of The University of Queensland until 2021. It will then be deposited in a Gundjeihmi Aboriginal Corporation keeping place. The material will be publicly accessible upon request from Gundjeihmi Aboriginal Corporation (gundjeihmi@mirarr.net). The language, images and information contained in this publication includes reference to Indigenous knowledge including traditional knowledge, traditional cultural expression and references to biological resources (plants and animals) of the Mirarr people. The source Indigenous knowledge is considered "Confidential Information"; traditional law and custom applies to it and the Mirarr people assert copyright over it in addition to any copyright in the complete work. Any Mirarr related language, images and information are published with the consent of Gundjeihmi Aboriginal Corporation as the representative of the Mirarr people for the purposes of general education purposes. No further use and absolutely no commercial use

is authorised without the prior consent and agreement of the Mirarr people. Please contact Gundjeihmi Aboriginal Corporation to request permission to refer to any Indigenous knowledge in this publication.

Field-specific reporting

Please select the one below that is the best fit for your research. If you are not sure, read the appropriate sections before making your selection.

☐ Life sciences ☐ Behavioural & social sciences ☒ Ecological, evolutionary & environmental sciences

For a reference copy of the document with all sections, see nature.com/documents/nr-reporting-summary-flat.pdf

Ecological, evolutionary & environmental sciences study design

All studies must disclose on these points even when the disclosure is negative.

Study description	This research was designed to test the capacity of archaeological Pandanus spiralis endocarps to investigate past fluctuations in MAP at the site of Madjedbebe, northern Australia.
Research sample	P. spiralis was chosen for this research as it is both identifiable and abundant in the plant macrofossil assemblage at Madjedbebe, and because other species of pandanus are found in archaeological sites across the tropics, including as part of Melanesian and Pacific foraging and agricultural systems, making results from this study of use to future archaeological and palaeoenvironmental work in this region.
Sampling strategy	Modern sample size was determined using a power calculation. For power 0.80 and alpha 0.05 two-tailed test of significance, if mean δ13C value is -27 for high WUE and -30 for low WUE and assuming SD 2.5 (15), then the sample size for each group should be 11. This sample size was reached (and often surpassed) in all cases but two. Archaeological sample size was 100% of all Pandanus spiralis endocarp >2mm in size, that had not already been mounted on an Scanning Electron Microscopy stubs for identification.
Data collection	Modern nutshells were from different environments in the ALligator RIvers region and from a transect along the Stuart Highway from Darwin to Katherine. Archaeological samples were recovered using flotation. All analysed samples were used.
Timing and spatial scale	Modern samples were collected in the Alligator Rivers region across three seasons (years) to make sure environmental data was not skewed by a particularly wet or dry season. They were only collected in one season across the transect as this portion of the study tested changes in mean annual precipitation across regions, rather than change across seasons. Archaeological samples were the product of the 2012 excavation at Madjedbebe and cover the past 65ky.
Data exclusions	Data from other plant species, including Canarium australianum and Pandanus basedowii, were excluded as the former was not found to be a particularly useful candidate for a precipitation proxy and we were able to exclude the latter as one of the species recovered from Madjedbebe.
Reproducibility	Unless otherwise stated, all results listed are the mean of repeat measurements with the standard deviation of the replicate analyses less than or equal to ±0.3.
Randomization	Modern samples were grouped via analytical units (e.g. vegetation communities and mean annual precipitation). Archaeological samples were grouped by excavation unit and by phase, a dated period of occupation at Madjedbebe.
Blinding	No blinding was used.

Did the study involve field work? ☒ Yes ☐ No

Field work, collection and transport

Field conditions	Fieldwork was completed across several seasons in the Northern Territory. Temperatures were above 30ºC and humidity was often high.
Location	Exact coordinates for each modern sample is found in the online supporting Information. The samples were collected from Madjedbebe, the Alligator Rivers region and the edge of the Stuart Highway from Darwin to Katherine.
Access & import/export	All relevant permits were obtained and samples were imported into Queensland in a state (dried) deemed acceptable by customs.
Disturbance	Minimal disturbance was caused by the study. Pandanus spiralis is a very common tree in the Northern Territory savanna and taking its fruit did not cause any damage to any of the trees.

Reporting for specific materials, systems and methods

We require information from authors about some types of materials, experimental systems and methods used in many studies. Here, indicate whether each material, system or method listed is relevant to your study. If you are not sure if a list item applies to your research, read the appropriate section before selecting a response.

Materials & experimental systems

n/a	Involved in the study
☒	☐ Antibodies
☒	☐ Eukaryotic cell lines
☐	☒ Palaeontology and archaeology
☒	☐ Animals and other organisms
☒	☐ Human research participants
☒	☐ Clinical data
☒	☐ Dual use research of concern

Methods

n/a	Involved in the study
☒	☐ ChIP-seq
☒	☐ Flow cytometry
☒	☐ MRI-based neuroimaging

Palaeontology and Archaeology

Specimen provenance

Madjedbebe archaeological site, northern Australia

Specimen deposition

Archaeobotanical material analysed in this study will be kept in the Archaeology Laboratories of The University of Queensland until 2021. It will then be deposited in a Gundjeihmi Aboriginal Corporation keeping place. The material will be publicly accessible upon request from Gundjeihmi Aboriginal Corporation (gundjeihmi@mirarr.net).

Dating methods

Three charcoal samples from C3/4, C3/5 and C3/7 were pre-treated using the ABA method. The pre-treated samples were combusted and then converted to graphite46. Radiocarbon analysis was carried out using the VEGA AMS Facility at ANSTO47. The radiocarbon results were converted to calendar ages using the SHCal13 data48 and OxCal program49.

☒ Tick this box to confirm that the raw and calibrated dates are available in the paper or in Supplementary Information.

Ethics oversight

Gundjeihmi Aboriginal Corporation, Australian Commonwealth Government, University of Queensland

Note that full information on the approval of the study protocol must also be provided in the manuscript.
