# Supplementary material for: Pandanus nutshell generates a palaeoprecipitation record for human occupation at Madjedbebe, northern Australia
Source: Nat Ecol Evol. 2021 Jan 25;5(3):295–303. doi: 10.1038/s41559-020-01379-8 (PMC7929916; doi:10.1038/s41559-020-01379-8)
Supplement: Supplementary file 3 — Peer review information [file 41559_2020_1379_MOESM3_ESM.pdf]

natureresearch

Peer Review Information

Journal: Nature Ecology & Evolution
Manuscript Title: Pandanus nutshell generates a palaeoprecipitation record for human occupation at Madjedbebe, northern Australia
Corresponding author name(s): S. Anna Florin, Andrew S. Fairbairn, Chris Clarkson

Reviewer Comments & Decisions:

Decision Letter, initial version:

4th August 2020

*Please ensure you delete the link to your author homepage in this e-mail if you wish to forward it to your co-authors.

Dear Ms Florin,

Your Article, "New palaeoprecipitation record associated with first human settlement of Australia" has now been seen by three reviewers. You will see from their comments copied below that while they find your work of considerable potential interest, they have raised quite substantial concerns that must be addressed. In light of these comments, we cannot accept the manuscript for publication, but would be very interested in considering a revised version that addresses these serious concerns.

We hope you will find the reviewers' comments useful as you decide how to proceed. If you wish to submit a substantially revised manuscript, please bear in mind that we will be reluctant to approach the reviewers again in the absence of major revisions.

In particular, we are concerned by reviewer 2's comments on the validity of pandanus endocarp d13C values as a proxy for water availability and will need to see these thoroughly addressed.

If you choose to revise your manuscript taking into account all reviewer and editor comments, please highlight all changes in the manuscript text file [OPTIONAL: in Microsoft Word format].

We are committed to providing a fair and constructive peer-review process. Please do not hesitate to contact us if there are specific requests from the reviewers that you believe are technically impossible or unlikely to yield a meaningful outcome.

If revising your manuscript:

* Include a "Response to reviewers" document detailing, point-by-point, how you addressed each referee comment. If no action was taken to address a point, you must provide a compelling argument. This response will be sent back to the referees along with the revised manuscript.

* If you have not done so already we suggest that you begin to revise your manuscript so that it conforms to our Article format instructions at http://www.nature.com/natecolevol/info/final-submission. Refer also to any guidelines provided in this letter.

* Include a revised version of any required reporting checklist. It will be available to referees (and, potentially, statisticians) to aid in their evaluation if the manuscript goes back for peer review. A revised checklist is essential for re-review of the paper.

Please use the link below to submit a revised paper:

[REDACTED]

Note: This URL links to your confidential home page and associated information about manuscripts you may have submitted, or that you are reviewing for us. If you wish to forward this email to co-authors, please delete the link to your homepage.

If you wish to submit a suitably revised manuscript we would hope to receive it within 6 months. If you cannot send it within this time, please let us know. We will be happy to consider your revision so long as nothing similar has been accepted for publication at Nature Ecology & Evolution or published elsewhere.

Nature Ecology & Evolution is committed to improving transparency in authorship. As part of our efforts in this direction, we are now requesting that all authors identified as 'corresponding author' on published papers create and link their Open Researcher and Contributor Identifier (ORCID) with their account on the Manuscript Tracking System (MTS), prior to acceptance. This applies to primary research papers only. ORCID helps the scientific community achieve unambiguous attribution of all scholarly contributions. You can create and link your ORCID from the home page of the MTS by clicking on 'Modify my Springer Nature account'. For more information please visit please visit www.springernature.com/orcid.

Please do not hesitate to contact me if you have any questions or would like to discuss the required revisions further.

Thank you for the opportunity to review your work.

Yours sincerely,

[REDACTED]

Reviewer expertise:

Reviewer #1: Australian Pleistocene archaeology

Reviewer #2: stable isotope proxy development

Reviewer #3: general Australian palaeoecology

Reviewers' comments:

Reviewer #1 (Remarks to the Author):

This is a timely and impactful paper which makes a number of major contributions of relevance to Australian archaeology and climate modelling and more widely in the tropics in the Indo-Pacific region. Perhaps of greatest importance is the ability to generate a proximal proxy record of palaeoprecipitation from the site under study rather than remote lake or marine locales which by definition will be out of the optimal comparative climatic regime. Questions of climate change, human responses in economy and society, and downstream symbolic behaviours will be far more accessible and plausible with multiple data sets coupled from the same deposits with soil and pandanus isotopic records. The methodology for establishing variability in isotopic signatures, precipitation and 'water-logging' from modern samples seems appropriate to the archaeological specimens on the assumption the distributions have always sat in areas experiencing above 1200 mm rainfall.This is a well written and structured paper with few typographic errors. See Page 1. l 46 "This 'pattern' is" - insert word, and P 8 l 22 remove "severity of". The conclusions that earlier, LGM and late Holocene aridity has not led to paucity is persuasive. A restructuring of lithic resource procurement towards 'exotic' categories as a proxy of greater mobility and permeable boundaries works well here. The three peaks in exotics shows a restructuring rather than resource depression during these phases. This is a game-changing paper that deserves to be published by Nature.

Reviewer #2 (Remarks to the Author):

This paper presents a promising novel proxy for past precipitation in the form of d13C values of P. spiralis endocarp fragments and applies this proxy to samples from the archaeological site of Madjedbebe in northern Australia. The ground-truthing of this proxy with modern samples is well thought out and the number of samples is appropriate. However, I don't agree that in all cases the soil d13C data support the archaeobotanical d13C data and therefore that the authors can be sure that the endocarp d13C values reflect water availability during all periods. I also think there are a number of things that could be clarified further in the manuscript.

I will outline these issues in more detail by commenting on the manuscript in line order:

L69: The paper by Fiorentino et al. does not mention the use of archaeobotanical isotope values as a way reconstructing past temperature. It is also quite a generic reference to choose as it summarises a wide range of applications. Another reference focussing on the application of archaeobotanical isotope analysis to infer past precipitation might be better here.

L70: Replace "woods" with tree species

L87: Litre should not be capitalised here or elsewhere

L88: I think this should be rephrased to make it clearer that grey refers to cf. whereas black does not.

L97: Make it clear that modern P. spiralis drupes were collected here.

Figure 2a: How were the rainfall ranges determined? I don't understand why some have a 200 mm range whereas others are marked as ~x mm.

L148-167: Is there evidence for P spiralis further south than the study site throughout the archaeological period studied? This would demonstrate nicely that it was not at the geographical limit of its distribution at any point in the past.

Are there statistically significant differences in endocarp d13C values between the phases? The results of statistical test should be quoted here. Phase 7 is confusing as the median shown in Fig 3a is relatively high, but looking at Fig 3b this may be caused by the samples from c.60 BP. It is therefore a bit misleading to state that samples from Phase 7 have relatively high d13C values in general.

L179: There are no data available at https://osf.io/9tn4f/. Will the material be made available after acceptance?

Figure 4: I don't agree with the discussion of the soil d13C value data here. To me it looks like soil d13C values are higher in Phase 3, 4a and 6 and quite high in Phase 2. This doesn't agree with the text in L188.

L181: I think you should refer here to the fact that the soil d13C values have been published previously. It would also be useful to state that they have been acid treated to remove carbonates. This is important and at the moment can only be found in the SI of the original article.

L195: It isn't clear exactly where the soil samples were taken. From the SI, I think at 5 cm intervals vertically, but then why group them by Phase? What does the graph look like if they are plotted directly against depth, which is already on the x axis?

L207: It isn't possible from Fig 4 to date peak aridity because there is no date on the x axis. You need to clarify here how you dated these samples to 22-18 kya.

L217-222: I don't agree that there is a close correlation between the soil and pandanus d13C values. High soil d13C values do not always coincide with high Pandanus d13C values in Fig. 4. Therefore, I don't agree that you can be sure that there was always moderate precipitation in the region. In fact, in Phase 5, high Pandanus d13C values coincide with low soil d13C values, contrary to what is stated here. Evidence for Pandanus at this period at sites south of Madjedbebe could be used as evidence that it wasn't at the limit of required precipitation.

L244: I'm unsure whether something should follow "and," or whether it should be deleted.

L292: Was stable isotope analysis carried out on the alpha-cellulose extracted from the endocarp?

L302-303: Why was charring at 500oC chosen? The results demonstrate no significant difference in

d13C between the treatments so you should justify why one was chosen over the others. A good way to decide would be to compare the physical appearance (under SEM) of archaeological Pandanus with experimentally charred.

L317-328: It would have been useful to check whether ABA treatment had an effect on the d13C values of endocarp fragments. Remember that d13C measurement is much less sensitive to contamination that 14C measurements and therefore a pre-treatment protocol that is used in radiocarbon dating may not be necessary for stable isotope analyses. Given the high loss rate due to ABA, it would be worth investigating this further (or at least stating that this would be a useful study) if you want to recommend this proxy to be used at sites in the future.

L330-340: How many replicate measurements of the archaeological samples were carried out?

L348: Again, it would be useful to give a few more details about the soil samples – at least that they were acid pre-treated. It would also be useful to provide details of the standards used, uncertainty and replicates of the stable isotope analyses, if they are available, as they are not in the original Clarkson et al. 2017 publication.

Supplementary Information:
SI 1: How was the rainfall value determined? It would be useful to present the %C and C:N ratios of these samples as well, to allow researchers to assess the reliability of the measurements and the variability to be expected for these samples.

SI 2: I am very surprised that the ANOVA yields a non-significant result since by looking at the figure, uncharred (dried) endocarp consistently has a higher d13C value.

SI 3: Again, it would be useful to present the %C and C:N ratios of these samples. When not a result of one analysis, how many replicates were carried out for each sample?

SI 4: Why were the standards separated like this? Do the number of runs correspond to periods when the samples were run, and they were carried out in 5 batches, with time in between? It isn't clear here.
In Table 3, the difference in EM B2155 is 0.06, not 0.02.

Reviewer #3 (Remarks to the Author):

This manuscript presents a palaeoenvironmental 65,000 year precipitation study, making use of the archaeological site Madjebebe. It provides an interesting and valuable addition to our understanding terminal Pleistocene-Holocene climatic fluctuations, of the response of regional inhabitants as well as tropical vegetation, both for the savanna tropics in general, and for northern Australia in particular. The emphasis on Pandanus nutshell as a data test-case adds to the value and interest of the research.

I disclose that I am not a geochemist and not skilled to evaluate the robustness of the isotopic methodological aspect of the study. I have formulated my review as a series of overall comments and questions, rather than a line-by-line analysis.

Well written with few apparent typos (see repeat wording lines 227-228).

1. Title and keywords.

While the title is technically true, the time-period of first human arrival into the site and region is only one part of the picture provided and I suggest the wording be changed to reflect this. The current title, reads like a 'headline', and for example essentially excludes interests (and search-ability) toward anything younger than c.65, 000 years. Given the consistencies (acknowledging that certain clarifications are made, e.g. LGM) with pre-existing data, I see this manuscript more as a methodological study, and the title (along with keywords) should also represent this more.

Keyword 'Early Modern Human' is somewhat misleading and a more vegetation orientated term should replace this, 'Pandanus' as a minimum.

2. Figures.

Suggestions here of additional references for your Figures extends to your text.

Figure 1a. Your map looks very sparse. In undertaking general online searches based on terms in the caption (i.e. Pleistocene, monsoonal tropics, northern Sahul and the like) – what of studies such as,
• Field, E., McGowan, H.A., Moss, P.T. and Marx, S.K., 2017. A late Quaternary record of monsoon variability in the northwest Kimberley, Australia. Quaternary International, 449: 119-135.

And there seems to be a growing body of late Quaternary work from the nearby Darwin area, un-referenced in general with one paper in particular appearing relevant?
• Rowe, C., Wurster, C.M., Zwart, C., Brand, M., Hutley, L.B., Levchenko, V. and Bird, M.I., Vegetation over the last glacial maximum at Girraween Lagoon, monsoonal northern Australia. Quaternary Research, pp.1-14.

Given your mapping marine core MD98-2167, why not then other marine core sites (e.g. to the north) discussing both climate and vegetation?

As presented in,
• Van der Kaars, W.A., 1991. Palynology of eastern Indonesian marine piston-cores: a Late Quaternary vegetational and climatic record for Australasia. Palaeogeography, Palaeoclimatology, Palaeoecology, 85: 239-302.

Or summarised in,
• Kershaw, P. and van der Kaars, S., 2012. Australia and the southwest Pacific. Quaternary environmental change in the tropics, pg. 236-262.

Figure 2 (a & b). Why are the sample dots distributed horizontally across each box plot? If you only documented rainfall in the given brackets of 200mm (as indicated by your X axis key and via SI-1 Table 1) shouldn't the sample dots present themselves in one vertical line? This current presentation

suggests a finer-scale recording of rainfall, in which case this should be corrected within the supplementary table and mentioned in the methods.

3. Pandanus spiralis.

Check italics for Pandanus.

The supplementary section would benefit from a detailed plant and plant habitat description. Incorporating photos from your sample sites would also be insightful.

What are the human uses and/or reliance on Pandanus species, including P.spiralis? What is its status within human diets and/or as a practical material? If it is a food staple of last-resort and therefore predominantly collected in times of less choice, does that influence its presence/absence in the archaeological record? Does this have implications for how far people may travel to obtain it? In addition to sediment properties, does this influence low numbers mid Holocene, when vegetation may be considered richer in resources?

Please clarify the water inundation tolerance of P.spiralis. This aspect is confusing. You're suggesting 'waterlogging' is stressing a plant which prefers wetter habitats. Are you discussing differences between 'seasonal waterlogging' and 'prolonged waterlogging? Are you inferring surface water (and/or high water table) present across both wet-dry seasons as 'prolonged' and that somewhere in here sits the 'tipping point' water threshold for the species? Is this therefore more a measure of seasonality; the delivery of rainfall rather than rainfall (precipitation) per se? This depending; for lines 131-132, should the word 'seasonal' be removed?

I feel one of the main questions to ask of this manuscript is the accuracy with which a wetter-habitat plant reflects fluctuations (specifically the timing) in precipitation. To quote Brock (2001, Native plants of northern Australia, Reed New Holland Press) P.spiralis is 'commonly associated with swamps, lagoons, billabongs and fringes of floodplains, where it often forms dense stands, and beside freshwater streams; open forests and woodlands' (pg 276). Not dissimilar to the manuscript, but I would expect a lagged, buffered response by such habitats to declines in precipitation as well as the opposite, a quicker accumulation of moisture once precipitation begins to rise. How representative is this of a wider sclerophyll landscape? One example here (lines 114+) is the modern data result – where you state lowest recorded precipitation, and yet samples were collected within wet-lands?

Similar to the above query is the manuscript's concept of refugia and the use of refugia as representative of conditions across a region?

Sample collection: Any additional stress imposed by the highway itself?

Line 251. Give these questions, is 'efficiency' too strong a term?

4. Archaeological soil δ13C records as a control measure.

I query the degree to which archaeological-rockshelter deposits can be considered a full independent reflection of (palaeo)environments and I don't feel these deposits should be called 'a palaeoenvironmental record' as per line 183. I would suggest this is predominantly a human derived cultural deposit, with minor secondary material input from the surrounding vegetation environment.

As a true base from which to compare your Pandanus analyses, can you address as to why a sediment core (for example, equally a soil profile) wasn't collected off-site, at even a short distance from the rockshelter?

5. Supplementary section notes.

S7: A good summary, although perhaps the references here could be better reflected in Figure 1's map. Vice-versa for the feedback made for Figure 1.

Please note the original Nott and Prince (1999) Northern Territory plunge pool study had its chronology re-examined in, May, J.H., Preusser, F. and Gliganic, L.A., 2015. Refining late Quaternary plunge pool chronologies in Australia's monsoonal 'Top End'. Quaternary Geochronology, 30: 328-333.

The authors may have missed an opportunity to introduce a series of thoughts on plant habitats through the 65,000 year period (both in the SI and main text). This SI-7 section would benefit from palaeo-vegetation/ecological content where available, most particular indications of Pandanus suited habitat, in order to strengthen arguments regarding the presence of P.spiralis through time. Such expansion of text may serve to lessen the hesitant tone within lines such as 226-227 'The same may also be true of the LGM and Terminal Pleistocene'.

Author Rebuttal to Initial comments

We thank the editor and reviewers for their thoughtful critiques. Following Reviewer #2 and #3's comments, we re-examined both the modern and archaeological data and made several significant changes to the manuscript:

- **we have used more robust measurements, including an extrapolated model from 24 meteorological stations detailing mean annual precipitation (MAP) and mean daily evaporation, to describe the modern relationship between *Pandanus spiralis* $\delta^{13}C$ and MAP;**
- **we have, subsequently, re-evaluated the archaeological data, using this modern relationship to interpret the archaeological patterns; and**
- **we have reconsidered the use of soil $\delta^{13}C$ as an environmental proxy, acknowledging the anthropogenic, as well as, environmental processes involved in its production.**

As such, our interpretation and several of our figures have changed. These changes have, however, confirmed our original conclusions about the nature of human-environment interaction in this region.

The following paragraphs respond to the critique seriatim. Changes and additions are shown in both a tracked and a clean version of the revised text. The line and page numbers used correspond to the tracked version.

Reviewer #1:

1. Page 2, line 55. "This 'pattern' is" - insert word.

The word ‘pattern’ has been inserted.

2. Page 14, line 322. Remove "severity of".

Thank you for pointing this out. These duplicate words have been removed.

Reviewer #2:

<u>Main text</u>

1. Page 3, line 94. The paper by Fiorentino et al. does not mention the use of archaeobotanical isotope values as a way reconstructing past temperature. It is also quite a generic reference to choose as it summarises a wide range of applications. Another reference focussing on the application of archaeobotanical isotope analysis to infer past precipitation might be better here.

Thank you. We take your point and have replaced Fiorentino et al. (2015) with several other papers that demonstrate the use of stable isotope analysis of archaeological plant material to study changes in precipitation and temperature in direct association with records of human occupation and cultural change: Aguilera et al. (2012); Heaton (1999); Wallace et al. (2013, 2015).

2. Page 3, line 89. Replace “woods” with tree species.

‘Woods’ has been replaced with ‘tree species’.

3. Page 3, line 107. Litre should not be capitalised here or elsewhere.

All mentions of the word ‘litre’ (Page 4, line 123, 125, 126; Page 13, lines 288, 289) have been changed to lower case.

4. Page 3, lines 108, 109. I think this should be rephrased to make it clearer that grey refers to cf. whereas black does not.

Agreed. This has been rephrased. It now reads, "*The black area indicates numbers of P. spiralis endocarp and mesocarp by litres floated, respectively, and the grey area indicates numbers of cf. P. spiralis endocarp and mesocarp by litres floated, respectively*," (page 4, lines 124-126).

5. Page 4, line 131. Make it clear that modern P. spiralis drupes were collected here.

The word modern has been added to this sentence to make it clear that we are discussing the collection of modern, rather than archaeological *P. spiralis*.

6. Figure 2a (now Fig. 3b): How were the rainfall ranges determined? I don't understand why some have a 200 mm range whereas others are marked as ~x mm.

Thank you for drawing our attention to this. Initially we utilised MAP based on contours from previously published research in the Northern Territory (Bureau of Meteorology, 2020, Russell-Smith and Yates, 2007). However, under further review we acknowledge that a more robust measurement is needed to address our claims. Using 24 meteorological stations, we have interpolated MAP for our study area in a systematic way. Rather than grouping sites based on MAP ranges, we can now provide MAP for each site and place it in context with the $\delta^{13}C$ isotopic signature of the *P. spiralis* drupes. This $\delta^{13}C$-MAP relationship has been used to generate palaeoprecipitation for Madjedbebe. We have detailed this method under the heading 'Modern climate' in the Methods section and in SI Section 2. We have also used the results of this analysis to make changes to Fig. 3 (previously Fig. 2) and Fig. 5 (previously Fig. 4). Further to this, elements of the results have also been rewritten (pages 4-5, lines 129-146; page 6, lines 193-204; pages 10-11, lines 255-271).

7. Page 7-8, lines 217-238: Is there evidence for P spiralis further south than the study site throughout the archaeological period studied? This would demonstrate nicely that it was not at the geographical limit of its distribution at any point in the past.

Unfortunately, this evidence does not exist. To date, pandanus plant macrofossils have only been recovered from sites in the Alligator Rivers region – Madjedbebe and Anbangbang I. No research into past plant macro- or microfossils have occurred to the south of this region. However, we hope this work will stimulate further macrobotanical investigations in the region.

8. Page 10, line 253. There are no data available at https://osf.io/9tn4f/. Will the material be made available after acceptance?

Thank you for pointing this out. This data has now been made available.

9. Figure 4 (now Fig. 5): I don't agree with the discussion of the soil d13C value data here. To me it looks like soil d13C values are higher in Phase 3, 4a and 6 and quite high in Phase 2. This doesn't agree with the text in Page 11, Line 271.

We agree with your assessment and following from changes made to the interpretation of the modern data, and to a subsequent reconsideration of the data produced in Fig. 5 (previously Fig. 4) we have rewritten this section. It now reads:

> "*Fig. 5 compares the pandanus proxy for palaeoprecipitation with the previously published soil δ^{13}C record*[1]*, corrected for changing $\delta^{13}CO_{2ATM}$ and pCO_2, and the discard of stone artefacts and exotic raw materials at Madjedbebe. The soil δ^{13}C record is likely a product of both environmental and anthropogenic influences, deriving from a combination of vegetation growing within and immediately outside the site, vegetation brought in by natural mechanisms (e.g. wind, water), and vegetation brought in by humans for a range of reasons, including subsistence, bedding and fuel*[30]*. This makes its interpretation more complex. In northern Australia, an abundance of C_4 plants in the environment correlates with seasonal water availability, with a higher abundance of C_4 grasses occurring in locations where precipitation during the summer monsoon season is high*[31, 32]*. Therefore, the similar peaks (in Phase 3 and, albeit slightly later, in Phase 6) and troughs (in Phase 2, and at the end of Phase 4 and 7) in both records suggest a general agreement in variation in palaeoprecipitation at the site*," (Page 10-11, lines 255-271).

10. Page 10, line 255. I think you should refer here to the fact that the soil d13C values have been published previously. It would also be useful to state that they have been acid

treated to remove carbonates. This is important and at the moment can only be found in the SI of the original article.

We have included reference to the previous publication of the soil δ13C values here, "*Fig. 5 compares the pandanus proxy for palaeoprecipitation with the previously published soil $\delta^{13}C$ record[1]…*" And we have added an additional section to the methods, titled 'Soil Analysis', detailing the pre-treatment of the soil samples (Page 10, lines 255-256).

11. Page 13, line 27: It isn't clear exactly where the soil samples were taken. From the SI, I think at 5 cm intervals vertically, but then why group them by Phase? What does the graph look like if they are plotted directly against depth, which is already on the x axis?

This is a great suggestion. We have changed the graph, so that the soil and pandanus isotope values are plotted directly against depth in Fig. 5 (previously Fig. 4).

12. Page 13, lines 296-299: It isn't possible from Fig 4 to date peak aridity because there is no date on the x axis. You need to clarify here how you dated these samples to 22-18 kya.

We agree and have changed the wording of this section to:

> "*The $\delta^{13}C$ values from the soil record suggests peak aridity at Madjedbebe may have occurred at the height of MIS2, c.22–18 kya (see Fig. 4). Whilst the LGM is thought to have caused a period of heightened aridity across Australia after c. 30 kya, the Madjedbebe records suggest that precipitation may not have declined dramatically until the latter half of Phase 4*," (Page 13, lines 296-299).

The dates, c.22–18kya, refer to the height of MIS 2, which broadly corresponds to the latter half of Phase 4. Although, as the reviewer has rightly stated an exact set of dates cannot be inferred from this figure.

13. Page 14, lines 309-313: I don't agree that there is a close correlation between the soil and pandanus d13C values. High soil d13C values do not always coincide with high Pandanus d13C values in Fig. 4. Therefore, I don't agree that you can be sure that there was always moderate precipitation in the region. In fact, in Phase 5, high Pandanus

d13C values coincide with low soil d13C values, contrary to what is stated here. Evidence for Pandanus at this period at sites south of Madjedbebe could be used as evidence that it wasn't at the limit of required precipitation.

Thank you for pointing this out. We have changed the wording of this to state that the two proxies show a "general", rather than "close" correlation. Further, following from the changes made to the interpretation of the modern *P. spiralis* data, and in response to Reviewer #3, we have reframed our interpretation of the soil $\delta^{13}C$ values, understanding it to be an independent proxy affected by both environmental and anthropogenic processes (see above response to 9.).

14. Page 14, line 345. I'm unsure whether something should follow "and," or whether it should be deleted.

Our mistake! 'And' has been removed.

15. Page 16, line 394: Was stable isotope analysis carried out on the alpha-cellulose extracted from the endocarp?

Yes, for modern dried endocarp samples, stable isotope analysis was carried out on the extracted alpha-cellulose. However, for charred endocarps, the analysis was performed on ABA pre-treated samples (see Methods and Table S3).

16. Page 16, lines 405-406: Why was charring at 500oC chosen? The results demonstrate no significant difference in d13C between the treatments so you should justify why one was chosen over the others. A good way to decide would be to compare the physical appearance (under SEM) of archaeological Pandanus with experimentally charred.

We take your point. However, unlike wheat grains and other seeds, which are highly malleable with charring, the nature of sclerenchyma means that endocarp does not show obvious variability in physical appearance after charring at different temperatures. As no significant difference is shown between different physical pre-treatments, we chose 500ºC as it is a commonly prescribed combustion temperature for loss-on-ignition (LOI) protocols on plant material and soils (i.e.,Nelson and Sommers, 1996). This line of reasoning has been added to the methods:

"*500ºC was chosen as it is a commonly prescribed combustion temperature for loss-on-ignition protocols for plant materials*[51]," (page 16, lines 407-408).

17. Page 17, lines 458-459: It would have been useful to check whether ABA treatment had an effect on the d13C values of endocarp fragments. Remember that d13C measurement is much less sensitive to contamination that 14C measurements and therefore a pre-treatment protocol that is used in radiocarbon dating may not be necessary for stable isotope analyses. Given the high loss rate due to ABA, it would be worth investigating this further (or at least stating that this would be a useful study) if you want to recommend this proxy to be used at sites in the future.

We agree and have added the following to the methods:

"*As $\delta^{13}C$ measurements are less sensitive to contamination than ^{14}C measurements and many of the archaeological specimens dissolved during pre-treatment, we recommend that future studies test the necessity of ABA pre-treatment for stable isotope analyses*," (page 17, lines 469-472).

For the present study, however, this does not affect the results as the pre-treatment methods are the same for the archaeological and the modern charred samples.

18. Page 17, lines 474-484: How many replicate measurements of the archaeological samples were carried out?

A minimum of two measurements were performed on each sample, unless there was not enough sample material available (see Table S4). If the standard deviation between these replicates was greater than 0.3499 the analyses was repeated until the standard deviation was below this. The number of replicates performed has been added to Table S4.

19. Page 18, line 500: Again, it would be useful to give a few more details about the soil samples – at least that they were acid pre-treated. It would also be useful to provide details of the standards used, uncertainty and replicates of the stable isotope analyses, if they are available, as they are not in the original Clarkson et al. 2017 publication.

Agreed. We have added an additional section into the methods, titled ‘Soil Analysis’, clarifying this:

“*A 2 g sub-sample of sediment from selected contexts (see SI Section 8) was ground to a fine powder and treated with 2M HCl for 24 h to remove inorganic carbon. National Bureau of Standards NBS-19 was used to normalise the data to the VPDB scale. Analytical precision for replicate measurements of δ13C in NBS-19 was ±0.2‰ (2SD). The results of this analysis were previously reported in Clarkson et al.*[1],” (Page 18, lines 488-492).

Supplementary Information

1. SI 1 (now SI 3): How was the rainfall value determined?

Please see our above response to this (6.). We have changed the MAP values in this table (Table S3) and have added the values for mean daily evaporation.

2. SI 1 (now SI 3): It would be useful to present the %C and C:N ratios of these samples as well, to allow researchers to assess the reliability of the measurements and the variability to be expected for these samples.

We cannot provide this data. Due to the extremely small size of the archaeological samples it was decided to analyse them undiluted with the IRMS tuned to the highest possible sensitivity. For consistency all samples were analysed with the same IRMS method, so the sample size for all analyses was ~30-90ug. At this sample size all nitrogen peaks were below the EA detection limit and we did not measure the samples for d15N values. The carbon peaks were also below the calibrated range for the EA. However, they were within the calibrated range for the IRMS. All sample peak heights were within the range of standard peak heights (ie., the standards in each run were loaded with a variety of masses to cover a broader range of peak heights than expected for the samples). If a sample peak height was outside the range of standard peak heights the result was rejected and, where possible, the analysis was repeated.

3. SI 2 (now SI 4): I am very surprised that the ANOVA yields a non-significant result since by looking at the figure, uncharred (dried) endocarp consistently has a higher d13C value.

We have checked this result and it is correct. The raw data is published in Table S3 allowing for its further analysis if anyone so wishes.

4. SI 3 (now SI 5): Again, it would be useful to present the %C and C:N ratios of these samples. When not a result of one analysis, how many replicates were carried out for each sample?

Again, it is not possible to present the %C and C:N ratios for these samples. However, added the number of replicates analysed to this section (see Table S4).

5. SI 4 (no SI 6): Why were the standards separated like this? Do the number of runs correspond to periods when the samples were run, and they were carried out in 5 batches, with time in between? It isn't clear here. In Table 3, the difference in EM B2155 is 0.06, not 0.02.

Yes. This is correct. A lot of samples were run across the course of this study and they did not all occur in the same batch. The title of each table reads 'Isotope standards for …' with the corresponding ANSTO IDs for the samples run in that batch. We have added the following at the beginning of this section to clarify this:

> "*The isotope samples reported on in this paper were analysed in 5 separate batches and 37 runs. The below tables provide the Isotope standards for each of these batches.*"

And thank you, we have fixed that typo.

Reviewer #3:

1. While the title is technically true, the time-period of first human arrival into the site and region is only one part of the picture provided and I suggest the wording be changed to reflect this. The current title reads like a 'headline', and for example essentially excludes interests (and search-ability) toward anything younger than c.65,000 years. Given the consistencies (acknowledging that certain clarifications are made, e.g. LGM) with pre-existing data, I see this manuscript more as a methodological study, and the title (along with keywords) should also represent this more.

We agree and have changed the title to better reflect the methodological nature of the study. It now reads, '*Palaeoprecipitation data from Madjedbebe, northern Australia: A novel proxy from ancient pandanus*.'

2. Keyword 'Early Modern Human' is somewhat misleading and a more vegetation orientated term should replace this, 'Pandanus' as a minimum.

As pandanus is now in the title, we have not used it in the key words. However, we agree and have replaced early modern humans with human ecology, as we feel this reflects the conclusions of the study in regards to changing settlement patterns and Veth's (1989) refugium model.

3. Figure 1a. Your map looks very sparse. In undertaking general online searches based on terms in the caption (i.e. Pleistocene, monsoonal tropics, northern Sahul and the like) – what of studies such as,
   • Field, E., McGowan, H.A., Moss, P.T. and Marx, S.K., 2017. A late Quaternary record of monsoon variability in the northwest Kimberley, Australia. Quaternary International, 449: 119-135.
   And there seems to be a growing body of late Quaternary work from the nearby Darwin area, un-referenced in general with one paper in particular appearing relevant?
   • Rowe, C., Wurster, C.M., Zwart, C., Brand, M., Hutley, L.B., Levchenko, V. and Bird, M.I., Vegetation over the last glacial maximum at Girraween Lagoon, monsoonal northern Australia. Quaternary Research, pp.1-14.
   Given your mapping marine core MD98-2167, why not then other marine core sites (e.g. to the north) discussing both climate and vegetation?
   As presented in,
   • Van der Kaars, W.A., 1991. Palynology of eastern Indonesian marine piston-cores: a Late Quaternary vegetational and climatic record for Australasia. Palaeogeography, Palaeoclimatology, Palaeoecology, 85: 239-302.
   Or summarised in,
   • Kershaw, P. and van der Kaars, S., 2012. Australia and the southwest Pacific. Quaternary environmental change in the tropics, pg. 236-262.

Thank you for these suggestions. We have incorporated these, and a number of other records into Fig. 1a.

4. Figure 2 (a & b). Why are the sample dots distributed horizontally across each box plot? If you only documented rainfall in the given brackets of 200mm (as indicated by your X axis key and via SI-1 Table 1) shouldn't the sample dots present themselves in one vertical line? This current presentation suggests a finer-scale recording of rainfall, in which case this should be corrected within the supplementary table and mentioned in the methods.

Fig. 3 (previously Fig. 2) has changed significantly. It no longer includes this manner of displaying data (jittering), and now shows the relationship between MAP and $\delta^{13}C$ as a linear transgression.

5. Check italics for Pandanus.

We use italics when the Latin name *Pandanus spiralis* is used at any point in the text. However, the common name for this species, and the genera more broadly, is also pandanus. Therefore, there are times in text where we refer to the common name not the Latin name, and do not use italics. Whilst this may have caused confusion, it is correct.

6. The supplementary section would benefit from a detailed plant and plant habitat description. Incorporating photos from your sample sites would also be insightful.

We agree and have included a new figure in text (Fig. 2), which incorporates some more pictures and information about *P. spiralis*. We have also included a supplementary section (SI Section 1), which discusses *P. spiralis* and includes example pictures of the sample sites we collected from near Madjedbebe.

7. What are the human uses and/or reliance on *Pandanus* species, including *P. spiralis*? What is its status within human diets and/or as a practical material? If it is a food staple of last-resort and therefore predominantly collected in times of less choice, does that influence its presence/absence in the archaeological record? Does this have implications for how far people may travel to obtain it? In addition to sediment properties, does this influence low numbers mid Holocene, when vegetation may be considered richer in resources?

P. spiralis formed a substantial element of the early colonial diet of Indigenous Australians in the Alligator Rivers region and is also a valuable material in weaving and fibrecraft. Whilst extracting the edible kernels from the drupes is difficult, especially without metal, it was used

from first occupation at the site 65,000 years ago (Florin et al., 2020). This is likely because it is rich in fat, a nutrient that was likely not readily accessible from the lean meat available in the Alligator Rivers region. It would have always been present in the immediate vicinity of the site, although the formation of the freshwater wetlands near the site in Phase 7 would have resulted in large stands of the taxa on the Magela Creek floodplain and in close proximity to Madjedbebe for the first time (Cowie et al., 2000). The reduction of pandanus in the midden phases is consistent with the broader reduction in charred plant remains during these phases, and is, therefore, likely caused by the alkali pH of the sediments from these phases. As fat would have always been a necessity for human survival in this region, and no new sources were presented by the changing sea-level and new estuarine environments (shellfish is a lean meat), we feel this taphonomic change is the most likely reason for the reduction in pandanus at the site. We discuss this in-text at Page 7, lines 224-229.

8. Please clarify the water inundation tolerance of *P. spiralis*. This aspect is confusing. You're suggesting 'waterlogging' is stressing a plant which prefers wetter habitats. Are you discussing differences between 'seasonal waterlogging' and 'prolonged waterlogging? Are you inferring surface water (and/or high water table) present across both wet-dry seasons as 'prolonged' and that somewhere in here sits the 'tipping point' water threshold for the species? Is this therefore more a measure of seasonality; the delivery of rainfall rather than rainfall (precipitation) per se? This depending; for lines 131-132, should the word 'seasonal' be removed?

Unfortunately there are no experimental assessments of tolerance of inundation of *P. spiralis*. However, in the revised text (page 6, line 177) and figures (Fig. 3) we have provided further information to support our interpretation, including a comparison that shows how $\delta^{13}C$ varies among habitats with different inundation regimes, being significantly lower in open woodlands than in fringes of floodplains (see Fig 3b). We indicate that in areas with high MAP seasonal waterlogging is extended (page 6, lines 182-205), which removes confusion around permanent vs seasonal inundation raised by the reviewer. The drupes develop in the late wet season/early dry season, when plants are likely to be waterlogged, which influences their $\delta^{13}C$ values, becoming mature in June to October.

9. I feel one of the main questions to ask of this manuscript is the accuracy with which a wetter-habitat plant reflects fluctuations (specifically the timing) in precipitation. To quote Brock (2001, Native plants of northern Australia, Reed New Holland Press) *P. spiralis* is 'commonly associated with swamps, lagoons, billabongs and fringes of floodplains, where it often forms dense stands, and beside freshwater streams; open forests and woodlands' (pg 276). Not dissimilar to the manuscript, but I would expect a lagged, buffered response by such habitats to declines in precipitation as well as the opposite, a

quicker accumulation of moisture once precipitation begins to rise. How representative is this of a wider sclerophyll landscape? One example here (lines 114+) is the modern data result – where you state lowest recorded precipitation, and yet samples were collected within wet-lands?

Fig. 3b provides the range of isotope variation associated with local environmental settings. It highlights that while this local variation in habitat does have an effect on the water use efficiency of *P. spiralis*, it does not confound the overall precipitation/water balance pattern observed. Further to this, we expect any 'buffer' effect would occur at a timescale which is not applicable to the dataset.

10. Similar to the above query is the manuscript's concept of refugia and the use of refugia as representative of conditions across a region?

We agree that we must be cautious in interpolating the data from Madjedbebe to the whole of the Alligator Rivers region, especially as the different topography of this region (including the Arnhem Land Escarpment) may have affected local conditions. However, our data is the only data available during the LGM for this region and it is in keeping with recent modelling studies for northern Australia (Yan et al., 2018). We have added the following in-text:

"*Our results indicate the severity of glacial maxima and its effects on local vegetation during this period may be overestimated in this region. This is unsurprising as modelling studies (e.g.,* [36]*) demonstrate that increased seasonal contrasts during the LGM enhance the dynamic forcing of the monsoon in northern Australia. This is partially offset by the reduction in available moisture in the atmosphere, but overall a minor increase in monsoon strength is inferred*," (page 14, lines 323-329).

11. Sample collection: Any additional stress imposed by the highway itself?

The largest expected issue from sampling along the highway would be if drainage from the highway affected the pandanus sample sites. This was carefully avoided during the sampling process.

12. Page 14, line 354. Given these questions, is 'efficiency' too strong a term?

The only time 'efficiency' is used in the manuscript is as part of the scientific phrase, 'water use efficiency'. This is a concept in botanical and ecological literature and, as such, should not be changed. We believe, especially with the improvements made in this version of the manuscript, that the word 'efficacy' used in this line is not too strong a term to describe the proxy.

13. Archaeological soil δ13C records as a control measure. I query the degree to which archaeological-rockshelter deposits can be considered a full independent reflection of (palaeo)environments and I don't feel these deposits should be called 'a palaeoenvironmental record' as per line 183. I would suggest this is predominantly a human derived cultural deposit, with minor secondary material input from the surrounding vegetation environment. As a true base from which to compare your Pandanus analyses, can you address as to why a sediment core (for example, equally a soil profile) wasn't collected off-site, at even a short distance from the rockshelter?

We agree with you and have incorporated the following into our discussion of the results:

> "*The soil $\delta^{13}C$ record is likely a product of both environmental and anthropogenic influences, deriving from a combination of vegetation growing within and immediately outside the site, vegetation brought in by natural mechanisms (e.g. wind, water), and vegetation brought in by humans for a range of reasons, including subsistence, bedding and fuel[30]. This makes its interpretation more complex*," (page 11, lines 259-264).

14. S7 (now SI 9): A good summary, although perhaps the references here could be better reflected in Figure 1's map. Vice-versa for the feedback made for Figure 1.

We have added several additional proxies to Figure 1a and SI 9. We have also now explained the monsoon drivers during the LGM briefly in text:

> "*This is unsurprising as modelling studies (e.g., [36]) demonstrate that increased seasonal contrasts during the LGM enhance the dynamic forcing of the monsoon in northern Australia. This is partially offset by the reduction in available moisture in the atmosphere, but overall a minor increase in monsoon strength is inferred*," (page 14, lines 323-329).

15. Please note the original Nott and Prince (1999) Northern Territory plunge pool study had its chronology re-examined in, May, J.H., Preusser, F. and Gliganic, L.A., 2015. Refining late Quaternary plunge pool chronologies in Australia's monsoonal 'Top End'. Quaternary Geochronology, 30: 328-333.

Thank you. We have updated SI Section 9 (previously 7) to acknowledge this paper. It now reads, "*Waterfall plunge pools may have been active during the LGM*[16]*, although this data has been reinterpreted to be younger based on newer OSL ages*[17]."

16. The authors may have missed an opportunity to introduce a series of thoughts on plant habitats through the 65,000 year period (both in the SI and main text). This SI-7 section would benefit from palaeo-vegetation/ecological content where available, most particular indications of Pandanus suited habitat, in order to strengthen arguments regarding the presence of *P. spiralis* through time. Such expansion of text may serve to lessen the hesitant tone within lines such as 226-227 'The same may also be true of the LGM and Terminal Pleistocene'.

Thanks for this suggestion. However, we think this would be a step too far. There is a real paucity of good vegetation data from this region prior to the Holocene. We are aware of limited studies, such as Cassie Rowe's LGM paper from the Darwin region, and that there is work that is as yet unpublished. However, the marine cores give such averaged vegetation and are so prone to the effects of shoreline positions that they provide little help for a study at this scale.

References

AGUILERA, M., FERRIO, J. P., PÉREZ, G., ARAUS, J. L. & VOLTAS, J. 2012. Holocene changes in precipitation seasonality in the western Mediterranean Basin: a multi-species approach using δ13C of archaeobotanical remains. *Journal of Quaternary Science,* 27**,** 192-202.

CLARKSON, C., JACOBS, Z., MARWICK, B., FULLAGAR, R., WALLIS, L., SMITH, M., ROBERTS, R. G., HAYES, E., LOWE, K., CARAH, X., FLORIN, S. A., MCNEIL, J., COX, D., ARNOLD, L. J., HUA, Q., HUNTLEY, J., BRAND, H. E. A., MANNE, T., FAIRBAIRN, A., SHULMEISTER, J., LYLE, L., SALINAS, M., PAGE, M., CONNELL, K., PARK, G., NORMAN, K., MURPHY, T. & PARDOE, C. 2017. Human occupation of northern Australia by 65,000 years ago. *Nature,* 547**,** 306-310.

COWIE, I. D., SHORT, P. S. & OSTERKAMP MADSEN, M. 2000. *Floodplain Flora: A flora of the coastal floodplains of the Northern Territory, Australia,* Canberra, Australian Biological Resources Study.

FIORENTINO, G., FERRIO, J. P., BOGAARD, A., ARAUS, J. L. & RIEHL, S. 2015. Stable isotopes in archaeobotanical research. *Vegetation History and Archaeobotany,* 24**,** 215-227.
FLORIN, S. A., FAIRBAIRN, A. S. & CLARKSON, C. 2020. 65,000-year-old plant remains show the earliest Australians spent plenty of time cooking. *In:* HOPKIN, M. (ed.) *The Conversation.*
HEATON, T. H. E. 1999. Spatial, species, and temporal variations in the 13C/12C ratios of C3 plants: Implications for palaeodiet studies.
BUREAU OF METEOROLOGY 2020. *Climate Data Online* [Online]. Australian Government. Available: http://www.bom.gov.au/climate/data/ [Accessed].
NELSON, D. W. & SOMMERS, L. E. 1996. Loss-on-ignition method. *In:* SPARKS, D. L., PAGEE, A. L., HELMKE, P. A. & LOEPPERT, R. H. (eds.) *Methods of Soil Analysis, Part 3: Chemical methods.* Madison: John Wiley & Sons.
RUSSELL-SMITH, J. & YATES, C. P. 2007. Australian Savanna Fire Regimes: Context, Scales, Patchiness. *Fire Ecology,* 3**,** 48-63.
VETH, P. 1989. Islands in the interior: A model for the colonization of Australia's arid zone. *Archaeology of Oceania,* 24**,** 81-92.
WALLACE, M., JONES, G., CHARLES, M., FRASER, R., HALSTEAD, P., HEATON, T. H. E. & BOGAARD, A. 2013. Stable carbon isotope analysis as a direct means of inferring crop water status and water management practices. *World Archaeology,* 45**,** 388-409.
WALLACE, M. P., JONES, G., CHARLES, M., FRASER, R., HEATON, T. H. & BOGAARD, A. 2015. Stable carbon isotope evidence for Neolithic and Bronze Age crop water management in the Eastern Mediterranean and Southwest Asia. *PLoS One,* 10**,** e0127085.
YAN, M., WANG, B., LIU, J., ZHU, A. & NING, L. 2018. Understanding the Australian Monsoon change during the Last Glacial Maximum with a multi-model ensemble. *Climate of the Past,* 14**,** 2037-2052.

Decision Letter, first revision:

5th November 2020

*Please ensure you delete the link to your author homepage in this e-mail if you wish to forward it to your co-authors.

Dear Ms Florin,

Your manuscript entitled "Palaeoprecipitation data from Madjedbebe, northern Australia: A novel proxy

from ancient pandanus" has now been seen by our reviewers, and in the light of their advice I am delighted to say that we can in principle offer to publish it. First, however, we would like you to revise your paper to address the final points made by the reviewers, and to ensure that it is as brief as possible and complies with our Guide to Authors at http://www.nature.com/natecolevol/info/final-submission.

TRANSPARENT PEER REVIEW
Nature Ecology & Evolution offers a transparent peer review option for new original research manuscripts submitted from 1st December 2019. We encourage increased transparency in peer review by publishing the reviewer comments, author rebuttal letters and editorial decision letters if the authors agree. Such peer review material is made available as a supplementary peer review file. Please state in the cover letter 'I wish to participate in transparent peer review' if you want to opt in, or 'I do not wish to participate in transparent peer review' if you don't. Failure to state your preference will result in delays in accepting your manuscript for publication.
Please note: we allow redactions to authors' rebuttal and reviewer comments in the interest of confidentiality. If you are concerned about the release of confidential data, please let us know specifically what information you would like to have removed. Please note that we cannot incorporate redactions for any other reasons. Reviewer names will be published in the peer review files if the reviewer signed the comments to authors, or if reviewers explicitly agree to release their name. For more information, please refer to our FAQ page.

SPECIFIC POINTS:
In particular, while checking through the manuscript and associated files, we noticed the following specific points which we will need you to address:

1. A brief editorial summary of the paper will appear on the journal homepage with the link to the paper. This is our proposed summary: 'stable carbon isotope analysis of modern and archaeological pandanus nutshell from Australia's currently oldest known archaeological site of Madjedbebe reveals precipitation fluctuations over the last 65000 years". Please let us know of any factual inaccuracies.
2. We'll need a more declarative title that conforms to our style guide (no subtitles or punctuation, avoidance of priority claims like 'novel' and 'first'). How about something like "Pandanus nutshell generates a palaeoprecipitation record for the Pleistocene human occupation of Madjedbebe, northern Australia" (Strictly speaking we shouldn't have a comma in the title, but I think specifying location is desirable)
3. Please upload all main text figures as separate files, and ensure that each is cited within the main text
4. Please follow instructions below and in attached to create up to 10 extended data figures. The remaining supplementary information, figures and tables should be collated into a single pdf
5. Please ensure the main text is no longer than 3500 words long, excluding title, abstract and list of references
6. Please double check all code/data respository stable URLs are working prior to final submission
7. As in the title, novelty/priority claims in the text should be limited to the bare minimum—we recognise that with palaeoecological content this may be impossible to limit completely, but please use discretion.

GENERAL POINTS:
We will also need you to check through all of the following general points when preparing the final

version of your manuscript:

The main manuscript file should include the abstract, main text, methods, author contribution, data availability, code availability and competing interests statements, acknowledgements, references, and figure legends. Figures should be submitted separately as individual files. For details on other supporting material, please see below.

Title & Abstract:
Titles should give an idea of the main finding of the paper and ideally not exceed 90 characters (including spaces). We discourage the use of active verbs and do not allow punctuation.

The paper's abstract (about 150-200 words; no references) should serve both as a general introduction to the topic, and as a brief, non-technical summary of your main results and their implications. It should start by outlining the background to your work (why the topic is important) and the main question you have addressed (the specific problem that initiated your research), before going on to describe your new observations, main conclusions and their general implications. Because we hope that scientists across the wider ecology and evolution community will be interested in your work, the abstract should be as accessible as possible, explaining essential but specialised terms concisely. We suggest you show your abstract to colleagues in other fields to uncover any problematic concepts.

Figures:
Choosing the right electronic format for your figures at this stage will speed up the processing of your paper. We would like the figures to be supplied as vector files - EPS, PDF, AI or postscript (PS) file formats (not raster or bitmap files), preferably generated with vector-graphics software (Adobe Illustrator for example). Please try to ensure that all figures are non-flattened and fully editable. All images should be at least 300 dpi resolution (when figures are scaled to approximately the size that they are to be printed at) and in RGB colour format. Please do not submit Jpeg or flattened TIFF files. Please see our guidelines https://www.nature.com/documents/NRJs-guide-to-preparing-final-artwork.pdf for more details, and also our image policies http://www.nature.com/authors/editorial_policies/image.html.

We will edit your figures/tables electronically so they conform to Nature Ecology & Evolution style. If necessary, we will re-size figures to fit single or double column width. If your figures contain several parts, the parts should be labelled lower case a, b, and so on, and form a neat rectangle when assembled.

Figure legends must provide a brief description of the figure and the symbols used, within 350 words. This must include definitions of any error bars employed in the figures.

Should your Article contain any items (figures, tables, images, videos or text boxes) that are the same as (or are adaptations of) items that have previously been published elsewhere and/or are owned by a third party, please note that it is your responsibility to obtain the right to use such items and to give proper attribution to the copyright holder. This includes pictures taken by professional photographers and images downloaded from the internet. If you do not hold the copyright for any such item (in whole or part) that is included in your paper, please complete and return this Third Party Rights Table, and attach any grant of rights that you have collected.

Please check the PDF of the whole paper and figures (on our manuscript tracking system) VERY CAREFULLY when you submit the revised manuscript. This will be used as the 'reference copy' to make sure no details (such as Greek letters or symbols) have gone missing during file-transfer/conversion and re-drawing.

Supporting Information:
All Supporting Information must be submitted in accordance with the instructions in the attached Inventory of Supporting Information, and should fit into one of two categories:

1. EXTENDED DATA: Extended Data are an integral part of the paper and only data that directly contribute to the main message should be presented. These figures will be integrated into the full-text HTML version of your paper and will be appended to the online PDF. There is a limit of 10 Extended Data figures, and each must be referred to in the main text, cited as Extended Data 1, Extended Data 2, etc. Each Extended Data figure should be of the same quality as the main figures, and should be supplied at a size that will allow both the figure and legend to be presented on a single A4 page. Each figure should be submitted as an individual .jpg, .tif or .eps file with a maximum size of 10 MB each. All Extended Data figure legends must be provided in the attached Inventory of Supporting Information, not in the figure files themselves.

2. SUPPLEMENTARY INFORMATION: Supplementary Information is material that is essential background to the study but which is not practical to include in the printed version of the paper (for example, video files, large data sets and calculations). Each item must be detailed in the attached Inventory of Supplementary Information. Tables containing large data sets should be in Excel format, with the table number and title included within the body of the table. All textual information and any additional Supplementary Figures (which should be presented with the legends directly below each figure) should be provided as a single, combined PDF. Please note that we cannot accept resupplies of Supplementary Information after the paper has been formally accepted unless there has been a critical scientific error.

Additional Supplementary Figures and other items are not required to be referred to in your manuscript text (though they can be), but should be numbered as Supplementary Figure 1, not SI1, etc.

Methods & Notes:
Please include references for the Methods in the same list as those for the main text, following on sequentially after the main text references. Any citations in the Supplementary Information will need inclusion in a separate SI reference list.

Please include a data availability statement as a separate section after Methods but before references, under the heading "Data Availability”. This section should inform readers about the availability of the data used to support the conclusions of your study. This information includes accession codes to public repositories (data banks for protein, DNA or RNA sequences, microarray, proteomics data etc…), references to source data published alongside the paper, unique identifiers such as URLs to data repository entries, or data set DOIs, and any other statement about data availability. All data that support the findings of the study must be made available. If DOIs are provided, we also strongly encourage including these in the Reference list (authors, title, publisher (repository name), identifier, year). For more guidance on how to write this section please see:

http://www.nature.com/authors/policies/data/data-availability-statements-data-citations.pdf

Nature Research policies (https://www.nature.com/authors/policies/availability.html#data) include a strong preference for research data to be archived in public repositories and in some cases this is mandatory. If you need help complying with this policy, or need help depositing and curating your research data (including raw and processed data, text, video, audio and images) you should consider:

Contacting Springer Nature's Research Data Helpdesk (https://www.springernature.com/gp/authors/research-data-policy/helpdesk/12327114) for advice.
Finding a suitable data repository (https://www.springernature.com/gp/authors/research-data-policy/repositories/12327124) for your data.
Uploading your data to Springer Nature's Research Data Support service (https://springernaturedata.typeform.com/to/UeGGKT). Please note there are fees (https://www.springernature.com/gp/authors/research-data-policy/pricing/15499842) for using Springer Nature's Research Data Support service.

Finally, we require authors to include a statement of their individual contributions to the paper, such as experimental work, project planning, data analysis, etc., immediately after the acknowledgements. The statement should be short, and refer to authors by their initials. For details please see the Authorship section of our joint Editorial policies at
http://www.nature.com/authors/editorial_policies/authorship.html

We will not send your revised paper for further review if, in the editors' judgement, the referees' comments on the present version have been addressed. If the revised paper is in Nature Ecology & Evolution format, in accessible style and of appropriate length, we shall accept it for publication immediately.

Please resubmit electronically

* the final version of the text (not including the figures) in either Word or Latex.

* publication-quality figures. For more details, please refer to our Figure Guidelines, which is available here: https://www.nature.com/documents/NRJs-guide-to-preparing-final-artwork.pdf .

* any Extended Data and Supplementary Information, as per instructed, with
the associated Inventory document.

* copies of our reporting and editorial policy checklists even if they have not changed since the previous round of revision.

* a point-by-point response to any issues raised by our reviewers and to any editorial suggestions.

* any suggestions for cover illustrations, which should be provided at high resolution as electronic files. Please note that such pictures should be selected more for their aesthetic appeal than for their scientific content. I am sure you will understand that we cannot make any promise as to whether any of your suggestions might be selected for the cover of Nature Ecology & Evolution.

Please use the following link to access your home page:

[REDACTED]

*This url links to your confidential homepage and associated information about manuscripts you may have submitted or be reviewing for us. If you wish to forward this e-mail to co-authors, please delete this link to your homepage first.

Please also send the following forms as a hand-signed PDF by email to ecoevo@nature.com.

*Please sign and return the Licence to Publish form

Or, if the corresponding author is a Crown government employee (including Great Britain and Northern Ireland, Canada and Australia), please sign
and return the Licence to Publish form for Crown government employees , or the Licence to Publish form for US government employees

For more information on our licence policy, please consult http://npg.nature.com/authors.

AUTHORSHIP

CONSORTIA -- For papers containing one or more consortia, all members of the consortium who contributed to the paper must be listed in the paper (i.e., print/online PDF). If necessary, individual authors can be listed in both the main author list and as a member of a consortium listed at the end of the paper. When submitting your revised manuscript via the online submission system, the consortium name should be entered as an author, together with the contact details of a nominated consortium representative. See https://www.nature.com/authors/policies/authorship.html for our authorship policy and https://www.nature.com/documents/nr-consortia-formatting.pdf for further consortia formatting guidelines, which should be adhered to prior to acceptance.

ORCID

Nature Ecology & Evolution is committed to improving transparency in authorship. As part of our efforts in this direction, we are now requesting that all authors identified as ‘corresponding author’ create and link their Open Researcher and Contributor Identifier (ORCID) with their account on the Manuscript Tracking System (MTS) prior to acceptance. ORCID helps the scientific community achieve unambiguous attribution of all scholarly contributions. For more information please visit http://www.springernature.com/orcid

For all corresponding authors listed on the manuscript, please follow the instructions in the link below to link your ORCID to your account on our MTS before submitting the final version of the manuscript. If you do not yet have an ORCID you will be able to create one in minutes.
https://www.springernature.com/gp/researchers/orcid/orcid-for-nature-research

IMPORTANT: All authors identified as ‘corresponding author’ on the manuscript must follow these

instructions. Non-corresponding authors do not have to link their ORCIDs but are encouraged to do so. Please note that it will not be possible to add/modify ORCIDs at proof. Thus, if they wish to have their ORCID added to the paper they must also follow the above procedure prior to acceptance.

To support ORCID's aims, we only allow a single ORCID identifier to be attached to one account. If you have any issues attaching an ORCID identifier to your MTS account, please contact the Platform Support Helpdesk.

We hope that you will support this initiative and supply the required information. Should you have any query or comments, please do not hesitate to contact me.

Nature Research journals encourage authors to share their step-by-step experimental protocols on a protocol sharing platform of their choice. Nature Research's Protocol Exchange is a free-to-use and open resource for protocols; protocols deposited in Protocol Exchange are citable and can be linked from the published article. More details can found at www.nature.com/protocolexchange/about.

We hope to hear from you within two weeks; please let us know if the revision process is likely to take longer.

Yours sincerely,

[REDACTED]

****
Reviewer Expertise:

Reviewer #1:
Reviewer #2:
Reviewer #3:

Reviewer Comments:

Reviewer #2 (Remarks to the Author):

I am happy with the revisions that have been made to the paper in the light of my and other reviewer's previous comments.

Reviewer #3 (Remarks to the Author):

Thankyou for the opportunity to follow-up in re-reading Florin et al. 'Palaeoprecipitation data from Madjedbebe, northern Australia: a novel proxy from ancient pandanus' (NATECOLEVOL-200610740A).

For your reassurance, I have read the author's response to all three reviewers, the revised manuscript

as well as new and/or updated supplementary documents. As requested by editorial staff I have ensured statistical analyses were concentrated upon.

I feel this is a comprehensive revision, with additions and adjustment encompassing text and figures. This manuscript benefits in particular from refinements in the modern supportive-data study design, the reconfiguration of the now Figure 3 and 5 (with rephrasing of passages associated with these figures) and the consideration of soil d13C as both environmental and anthropogenic. The provision of ecological background pertaining to Pandanus, now makes for a well-rounded presentation of the endocarp as a proxy source.

Please note the spelling of 'Katherine' as 'Catherine' in the SI. Likely an autocorrect factor.

I have no further issue with this submission and recommend publication.

Congratulations on a nice piece of research and I hope you receive beneficial interest and discussions once in print.

********************END********************

Final Decision Letter:

3rd December 2020

Dear Anna,

We are pleased to inform you that your Article entitled "Pandanus nutshell generates a palaeoprecipitation record for human occupation at Madjedbebe, northern Australia", has now been accepted for publication in Nature Ecology & Evolution.

Before your manuscript is typeset, we will edit the text to ensure it is intelligible to our wide readership and conforms to house style. We look particularly carefully at the titles of all papers to ensure that they are relatively brief and understandable.

The subeditor may send you the edited text for your approval. Once your manuscript is typeset you will receive a link to your electronic proof via email within 20 working days, with a request to make any corrections within 48 hours. If you have queries at any point during the production process then please contact the production team at rjsproduction@springernature.com. Once your paper has been scheduled for online publication, the Nature press office will be in touch to confirm the details.

Acceptance of your manuscript is conditional on all authors' agreement with our publication policies (see www.nature.com/authors/policies/index.html). In particular your manuscript must not be published elsewhere and there must be no announcement of the work to any media outlet until the publication date (the day on which it is uploaded onto our web site).

The Author's Accepted Manuscript (the accepted version of the manuscript as submitted by the author) may only be posted 6 months after the paper is published, consistent with our self-archiving embargo. Please note that the Author’s Accepted Manuscript may not be released under a Creative Commons license. For Nature Research Terms of Reuse of archived manuscripts please see: http://www.nature.com/authors/policies/license.html#terms
If you have posted a preprint on any preprint server, please ensure that the preprint details are updated with a publication reference, including the DOI and a URL to the published version of the article on the journal website.

An online order form for reprints of your paper is available at https://www.nature.com/reprints/author-reprints.html. All co-authors, authors' institutions and authors' funding agencies can order reprints using the form appropriate to their geographical region.

We welcome the submission of potential cover material (including a short caption of around 40 words) related to your manuscript; suggestions should be sent to Nature Ecology & Evolution as electronic files (the image should be 300 dpi at 210 x 297 mm in either TIFF or JPEG format). Please note that such pictures should be selected more for their aesthetic appeal than for their scientific content, and that colour images work better than black and white or grayscale images. Please do not try to design a cover with the Nature Ecology & Evolution logo etc., and please do not submit composites of images related to your work. I am sure you will understand that we cannot make any promise as to whether any of your suggestions might be selected for the cover of the journal.

You can now use a single sign-on for all your accounts, view the status of all your manuscript submissions and reviews, access usage statistics for your published articles and download a record of your refereeing activity for the Nature journals.

To assist our authors in disseminating their research to the broader community, our SharedIt initiative provides you with a unique shareable link that will allow anyone (with or without a subscription) to read the published article. Recipients of the link with a subscription will also be able to download and print the PDF.

You can generate the link yourself when you receive your article DOI by entering it here: http://authors.springernature.com/share<a>.

[REDACTED]

P.S. Click on the following link if you would like to recommend Nature Ecology & Evolution to your librarian http://www.nature.com/subscriptions/recommend.html#forms

** Visit the Springer Nature Editorial and Publishing website at <a href="http://editorial-jobs.springernature.com?utm_source=ejP_NEcoE_email&utm_medium=ejP_NEcoE_email&utm_campa

ign=ejp_NEcoE">www.springernature.com/editorial-and-publishing-jobs for more information about our career opportunities. If you have any questions please click here.**
